# Androgen Receptor Pathway Inhibitor Therapy for Advanced Prostate Cancer: Secondary Analysis of a Randomized Clinical Trial

**DOI:** 10.1001/jamanetworkopen.2024.54253

**Published:** 2025-01-13

**Authors:** Diogo Assed Bastos, Andrey Soares, Fabio Augusto Barros Schutz, Eduardo Cronemberger, Murilo de Almeida Luz, Suelen Patricia Dos Santos Martins, David Queiroz Borges Muniz, Flavio Mavignier Cárcano, Oren Smaletz, Fábio Affonso Peixoto, Andrea Juliana Gomes, Felipe Melo Cruz, Fábio André Franke, Daniel Herchenhorn, Rosemarie Gidekel, Gustavo Werutsky, Taiane Francieli Rebelatto, Rafaela Gomes de Jesus, Vinicius Carrera Souza, André Poisl Fay, Fernando Cotait Maluf

**Affiliations:** 1 Latin American Cooperative Oncology Group (LACOG), Porto Alegre, Brazil; 2 Department of Medical Oncology, Hospital Sírio-Libanês, São Paulo, Brazil; 3 Department of Medical Oncology, Hospital Israelita Albert Einstein, São Paulo, Brazil; 4 Centro Paulista de Oncologia–Oncoclinicas, São Paulo, Brazil; 5 Department of Medical Oncology, Beneficência Portuguesa de São Paulo, São Paulo, Brazil; 6 Centro Regional Integrado de Oncologia, Fortaleza, Brazil; 7 Department of Medical Oncology, Hospital Erasto Gaertner, Curitiba, Brazil; 8 Centro de Estudos e Pesquisa de Hematologia–Centro Universitário Faculdade de Medicina do ABC (CEPHO-FMABC), Santo André, Brazil; 9 Instituto do Câncer do Estado de São Paulo (ICESP), São Paulo, Brazil; 10 Hospital de Câncer de Barretos, Barretos, Brazil; 11 Instituto COI de Educação, Pesquisa e Gestão em Saúde, Rio de Janeiro, Brazil; 12 Liga Norte Riograndense Contra o Câncer, Natal, Brazil; 13 IBCC Oncologia–Centro Universitário São Camilo, São Paulo, Brazil; 14 Oncosite-Centro de Pesquisa Clínica, Ijuí, Brazil; 15 Oncologia D’OR/Instituto D’OR de Ensino e Pesquisa, Rio de Janeiro, Brazil; 16 Now with Janssen Pharmaceuticals, Latin America, Buenos Aires, Argentina; 17 Oncologia D’OR, Salvador, Brazil; 18 Pontifícia Universidade Católica do Rio Grande do Sul (PUCRS) School of Medicine, Porto Alegre, Brazil

## Abstract

**Importance:**

The open-label randomized phase 2 LACOG0415 trial evaluated 3 treatment strategies for patients with advanced castration-sensitive prostate cancer (CSPC): androgen deprivation therapy (ADT) plus abiraterone acetate and prednisone (AAP), apalutamide (APA) alone, or APA plus AAP.

**Objective:**

To investigate the association of ADT plus AAP, APA alone, or APA plus AAP with health-related quality of life (HRQOL) in patients with advanced CSPC in the LACOG0415 trial.

**Design, Setting, and Participants:**

The LACOG0415 randomized clinical trial comprised 128 patients with advanced CSPC who were randomized (1:1:1) to 1 of 3 treatment arms from October 16, 2017, to April 23, 2019. Statistical analysis was conducted from March to September 2022.

**Interventions:**

Patients were randomized (1:1:1) to 1 of 3 treatment arms: ADT plus AAP, APA alone, or APA plus AAP.

**Main Outcomes and Measures:**

Health-related quality of life was evaluated using the Functional Assessment of Cancer Therapy–Prostate (FACT-P) questionnaire, including its subscales, completed at baseline and every 4 weeks until week 25. FACT-P scores range from 0 to 156, and higher scores indicate better HRQOL. Mean changes in score from baseline to week 25 were adjusted by baseline score and were calculated to evaluate whether there was a difference according to the treatment arm using a mixed-effect model for repeated measures. Time to deterioration was estimated by Kaplan-Meier curves and compared by stratified log-rank test. Analysis was performed on an intention-to-treat basis.

**Results:**

A total of 128 patients with advanced CSPC were randomized to receive ADT plus AAP (n = 42; median age, 69.8 years [IQR, 58.9-71.6 years]), APA alone (n = 42; median age, 69.5 years [IQR, 59.8-72.6 years]), or APA plus AAP (n = 44; median age, 71.0 years [IQR, 63.0-72.3 years]). Metastatic disease was present in 95 patients (74.2%), high-risk biochemical recurrence disease in 22 (17.2%), and locally advanced disease in 11 (8.6%). There was no significant difference in baseline mean (SD) FACT-P total scores and subscales among the 3 treatment arms (FACT-P total score: ADT plus AAP arm, 118.5 [24.3]; APA alone arm, 116.1 [23.9]; AAP plus APA arm, 114.9 [18.1]; *P* = .69). Health-related quality of life was maintained during treatment period, and there were no statistically significant differences at 25 weeks in mean (SD) FACT-P total scores or subscales between treatment arms (FACT-P total score: ADT plus AAP arm, 122.3 [20.4]; APA alone arm, 119.5 [16.4]; AAP plus APA arm, 119.9 [20.3]). The APA alone and AAP plus APA arms were not associated with meaningful improvements in HRQOL compared with the ADT plus AAP arm, except in time to deterioration of the emotional well-being score, which was more favorable in the APA alone arm (reference arm: ADT plus AAP arm; APA alone arm: hazard ratio, 0.37 [0.15-0.85]; *P* = .02; ADT plus AAP arm: hazard ratio, 0.56 [0.26-1.19]; *P* = .13). Limitations include short follow-up period and the absence of other questionnaires to capture differences between therapies.

**Conclusions and Relevance:**

In this prespecified secondary analysis of a randomized clinical trial of ADT plus AAP, APA alone, or APA plus AAP for patients with advanced CSPC, HRQOL was not statistically different between treatments with APA alone or APA plus AAP as compared with ADT plus AAP. Larger studies with longer follow-up and more specific questionnaires are needed to further evaluate HRQOL with these treatment strategies.

**Trial Registration:**

ClinicalTrials.gov Identifier: NCT02867020

## Key Points

**Question:**

What is the association of apalutamide (APA) alone or APA plus abiraterone acetate plus prednisone (AAP) with health-related quality of life (HRQOL) in patients with advanced castration-sensitive prostate cancer?

**Findings:**

In this prespecified secondary analysis of a randomized phase 2 trial involving 128 patients, no statistically significant differences in HRQOL were observed among those receiving androgen deprivation therapy (ADT) plus AAP, APA alone, or APA plus AAP, except in time to deterioration of the emotional well-being score, which was more favorable in the APA alone group than the ADT plus AAP group.

**Meaning:**

This study suggests that HRQOL remained stable in all 3 groups during treatment, without meaningful improvements in HRQOL in the APA alone and AAP plus APA groups compared with the ADT plus AAP group.

## Teaser

This prespecified secondary analysis of a randomized clinical trial investigates the association of apalutamide alone or apalutamide plus abiraterone acetate and prednisone with health-related quality of life in patients with advanced castration-sensitive prostate cancer.

## Introduction

Over the past years a marked improvement in oncologic outcomes has been achieved with more-intensive treatments for patients with advanced castration-sensitive prostate cancer (CSPC), including a substantial increase in median overall survival, which now approaches 60 months.^1^ These landmark results have been obtained with androgen deprivation therapy (ADT) intensification with docetaxel, next-generation androgen receptor pathway inhibitors (ARPIs), or a combination of chemotherapy and ARPIs.^2,3,4,5,6,7,8^ With longer survival, despite better control of cancer-related symptoms, patients may experience an increase in both acute and chronic treatment-related toxic effects, which may affect well-being and health-related quality of life (HRQOL).

Toxic effects associated with ADT are well recognized and affect several aspects of HRQOL by causing fatigue, hot flushes, weight gain, sarcopenia, loss of libido, and decreased bone mineral density, among others.^9,10^ This effect on HRQOL may be even more evident with ARPIs, which are associated with longer treatment exposure and may cause additional adverse effects such as memory loss, risk of falls and fractures, hypertension, and increased risk of cardiovascular events.^3,4,5^ Nevertheless, HRQOL analysis of these contemporary studies comparing ADT vs ADT plus ARPIs demonstrated that the addition of either abiraterone acetate plus prednisone (AAP), enzalutamide, or apalutamide (APA) led to a delay in HRQOL deterioration.^11,12,13,14^ This is achieved through a delay in cancer-related symptoms, ensuring a similar HRQOL compared with ADT alone despite an increase in treatment-related toxic effects.

As an attempt to maintain efficacy while minimizing ADT-related adverse effects, we conducted a randomized phase 2 clinical trial evaluating alternatives for advanced CSPC (LACOG0415 trial). This study included 3 treatment arms: ADT plus AAP, APA alone, and AAP plus APA, with a secondary objective of assessing patient-reported HRQOL. Results of the primary objective (prostate-specific antigen [PSA] <0.2 ng/mL at week 25 [to convert to micrograms per liter, multiply by 1.0]) have been previously reported.^15^ In this prespecified secondary analysis, we report the results from HRQOL analysis using the Functional Assessment of Cancer Therapy–Prostate (FACT-P) questionnaire.

## Methods

### Study Design

The LACOG0415 trial is a phase 2, open-label, noncomparative randomized clinical trial evaluating the efficacy of ADT plus AAP, APA alone, and APA plus AAP, at standard doses, among patients with advanced CSPC from October 16, 2017, to April 23, 2019. The primary end point was the proportion of patients who achieved a PSA level of 0.2 ng/mL or lower at week 25. Evaluation of HRQOL using the FACT-P questionnaire was a secondary end point. A detailed description of the trial design and eligibility criteria has been previously described.^15^ The trial was designed and led by the genitourinary steering committee from the Latin American Cooperative Oncology Group (LACOG) and conducted in 14 Brazilian sites. The study protocol was approved by the institutional review board of all participating sites and was conducted in accordance with Good Clinical Practice guidelines and the Declaration of Helsinki^16^ (trial protocol in Supplement 1). Written informed consent was obtained from all participants by investigators before any study procedure. This trial was registered in ClinicalTrials.gov (NCT02867020). All study reporting results adhered to the Consolidated Standards of Reporting Trials (CONSORT) reporting guideline.

### Patients

Patients with advanced CSPC were defined in this study as (1) patients with locally advanced prostate cancer with positive lymph nodes who were not candidates for radical surgery or radiotherapy and who presented with a PSA level of 2 ng/mL or higher; (2) patients with high-risk biochemical recurrence, defined as a PSA level of 4 ng/mL or higher and PSA doubling time less than 10 months, or a PSA level of higher than 20 ng/mL; or (3) patients with metastatic CSPC and a PSA level of 2 ng/mL or higher. All included patients had testosterone levels of 230 ng/dL or higher (to convert to nanomoles per liter, multiply by 0.0347) at baseline. Patients were excluded if they had received prior ADT except in the context of local therapy with an ADT-free interval greater than 12 months prior to study entry. The full eligibility criteria are available in eAppendix 1 in Supplement 2.

### Randomization and Study Treatment

Eligible patients were randomized (1:1:1) to receive ADT plus abiraterone acetate, 1000 mg, plus prednisone, 5 mg, twice daily (ADT plus AAP arm); APA, 240 mg daily (APA arm); and abiraterone acetate, 1000 mg, plus prednisone, 5 mg, twice daily, plus APA, 240 mg daily (AAP plus APA arm). Randomization was centrally performed by a randomization system.^17^ Randomization was balanced by using randomly permuted blocks. Patients were stratified by performance status (Eastern Cooperative Oncology Group 0-1 vs 2) and metastatic disease (yes vs no). Investigators and patients were not masked to treatment assignments. The full randomization procedure is available in eAppendix 2 in Supplement 2. Patients were treated until week 25, disease progression, unacceptable toxic effects, or consent withdrawal. Patients without disease progression were allowed to continue treatment beyond week 25 (extension phase) at investigator discretion. Patients were followed up for overall survival for 2 years.

### Health-Related Quality of Life

Health-related quality of life assessments were performed using the patient self-administered FACT-P questionnaire at baseline and every 4 weeks (±3 days) until week 25. FACT-P is a validated tool with 39 questions (maximum, 4 points each) to assess 4 domains of quality of life: physical well-being, functional well-being, emotional well-being (EWB), and social well-being (comprising the Functional Assessment of Cancer Therapy–General [FACT-G]), in addition to a 12-question prostate cancer subscale score that includes 4 pain-related questions and items related to prostate cancer and/or its treatment.^18^ FACT-P scores range from 0 to 156, and higher scores indicate better HRQOL.

### Mean Change From Baseline

A mixed-effects model for repeated measures (MMRM), which assumes that missing data are missing at random, was used to estimate longitudinal changes from baseline score. To assess the robustness of the MMRM results, a sensitivity analysis to address the possibility of the data missing not at random was performed using the pattern-mixture model with control-based pattern imputation (controlling for baseline score, treatment assignment, and treatment week).

### Time to Deterioration

Time to first HRQOL deterioration was assessed from randomization to first HRQOL deterioration. Differences greater than 10 points in FACT-P total score, 7 points in FACT-G total score, and 3 points in physical well-being, functional well-being, EWB, social well-being, and prostate cancer subscale scores were considered clinically significant. Time to deterioration was estimated by the Kaplan-Meier method and compared by stratified log-rank test.

### Statistical Analysis

Statistical analysis was performed from March to September 2022 on an intention-to-treat basis. The sample size was calculated using the Fleming 1-stage method based on the primary end point: proportion of patients with a PSA level of 0.2 ng/mL or lower at week 25. The study would have 80% power with 5% significance level to reject a rate of 45% or less of PSA levels of 0.2 ng/mL or lower at week 25, with an expected 65% rate of PSA levels of 0.2 ng/mL or lower at week 25 for each treatment arm. Detailed statistical analysis of LACOG0415 study has been previously reported.^15^ FACT-P questionnaires were considered for analysis if at least 1 question was answered at any assessment time point. Analysis of HRQOL change from baseline and deterioration included only patients with baseline scores and at least 1 postbaseline score. All statistical analyses were conducted using SAS, version 9.4 (SAS Institute Inc). Statistical tests were 2-sided and *P* < .05 was considered statistically significant without multiplicity adjustment. Adjustments for multiple comparisons were not made because these were exploratory analyses.

## Results

### Baseline Data

At 14 sites, 190 patients were screened and 128 were randomized (eFigure 3 in Supplement 2). A total of 42 patients (median age, 69.8 years [IQR, 58.9-71.6 years]) were assigned to the ADT plus AAP arm, 42 patients (median age, 69.5 years [IQR, 59.8-72.6 years]) to the APA alone arm, and 44 patients (median age, 71.0 years [IQR, 63.0-72.3 years]) to the APA plus AAP arm (eTable 1 in Supplement 2). Baseline demographics and disease characteristics were well balanced among the arms, including burden of disease at study entry—metastatic disease was present in 95 patients (74.2%): 29 of 42 (69.0%) in the ADT plus AAP arm, 32 of 42 (76.2%) in the APA alone arm, and 34 of 44 (77.3%) in the APA plus AAP arm. High-risk biochemical recurrence disease was present in 22 patients (17.2%) and locally advanced disease was present in 11 patients (8.6%). The FACT-P questionnaire was completed by 122 patients (95.3%) at baseline and by 102 (79.7%) at week 25 for the overall sample. The FACT-P questionnaire at baseline was completed by 40 of 42 patients (95.2%) in ADT plus AAP arm, 41 of 42 (97.6%) in the APA alone arm, and 41 of 44 (93.2%) in the APA plus AAP arm; at 25 weeks these frequencies were 32 of 42 (76.2%) in the ADT plus AAP arm, 35 of 42 (83.3%) in the APA only arm, and 35 of 44 (79.5%) in the APA plus AAP arm. The mean baseline FACT-P total scores and subscale scores were similar among study arms (Table 1; eTable 2 in Supplement 2).

**Table 1.** Health-Related Quality-of-Life Scores

HRQOL score	ADT plus AAP	APA alone	AAP plus APA
Baseline HRQOL score, mean (SD)			
Physical well-being	23.9 (5.1)	23.4 (5.0)	23.9 (4.0)
Functional well-being	19.6 (5.9)	19.2 (6.1)	18.8 (6.1)
Social well-being	21.4 (6.1)	21.4 (6.0)	20.6 (5.8)
Emotional well-being	19.7 (3.8)	18.6 (4.9)	19.2 (4.0)
Prostate cancer subscale	34.0 (8.7)	33.7 (7.1)	33.0 (6.1)
FACT-G total score	84.3 (17.2)	82.6 (17.9)	82.4 (13.7)
FACT-P total score	118.5 (24.3)	116.1 (23.9)	114.9 (18.1)
Week 25 HRQOL score, mean (SD)			
Physical well-being	24.5 (4.2)	23.6 (4.9)	23.2 (5.0)
Functional well-being	19.7 (5.7)	19.5 (4.4)	20.4 (5.1)
Social well-being	20.8 (5.2)	20.9 (4.9)	20.9 (4.0)
Emotional well-being	20.7 (4.3)	19.9 (2.5)	20.7 (3.5)
Prostate cancer subscale	36.2 (6.1)	35.3 (5.8)	34.4 (6.9)
FACT-G total score	86.1 (15.8)	84.1 (12.4)	85.4 (14.6)
FACT-P total score	122.3 (20.4)	119.5 (16.4)	119.9 (20.3)

Abbreviations: AAP, abiraterone acetate plus prednisone; ADT, androgen deprivation therapy; APA, apalutamide; FACT-G, Functional Assessment of Cancer Therapy–General; FACT-P, Functional Assessment of Cancer Therapy–Prostate; HRQOL, health-related quality of life.

### Efficacy and Safety

A PSA of 0.2 ng/mL or lower at week 25 was observed in 31 of 41 patients (75.6% [95% CI, 59.7%-87.6%]) in the ADT plus AAP arm, 24 of 40 patients (60.0% [95% CI, 43.3%-75.1%]) in the APA alone arm, and 31 of 39 patients (79.5% [95% CI, 63.%-90.7%]) in APA plus AAP arm.^15^ The 2-year overall survival was 92.5% (95% CI, 84.3%-100%) in the ADT plus AAP arm, 87.9% (95% CI, 77.9%-97.8%) in the APA alone arm, and 92.7% (95% CI, 84.8%-100%) in the APA plus AAP arm, without statistical significance by log-rank test (*P* = .59).^18^

Treatment-related adverse events (TRAEs) of any grade were observed in 30 of 42 patients (71.4%) in the ADT plus AAP arm, 34 of 42 patients (81.0%) in the APA alone arm, and 36 of 44 patients (81.8%) in the APA plus AAP arm (eTable 3 in Supplement 2). Grade 3 and 4 TRAEs were observed in 8 of 42 patients (19.0%) in the ADT plus AAP arm, in 7 of 42 patients (16.7%) in the APA alone arm, and in 10 of 44 patients (22.7%) in the APA plus AAP arm. Lower rates of hot flushes and hypertension were reported in the APA alone arm. Patients in the APA alone arm experienced a higher rate of gynecomastia (23 of 42 [54.8%]) and breast pain (6 of 42 [14.3]%) compared with the other 2 groups. No new safety signal was detected in the study.^15^

### Health-Related Quality of Life

#### FACT-P and FACT-G Questionnaires

**Figure 1.**
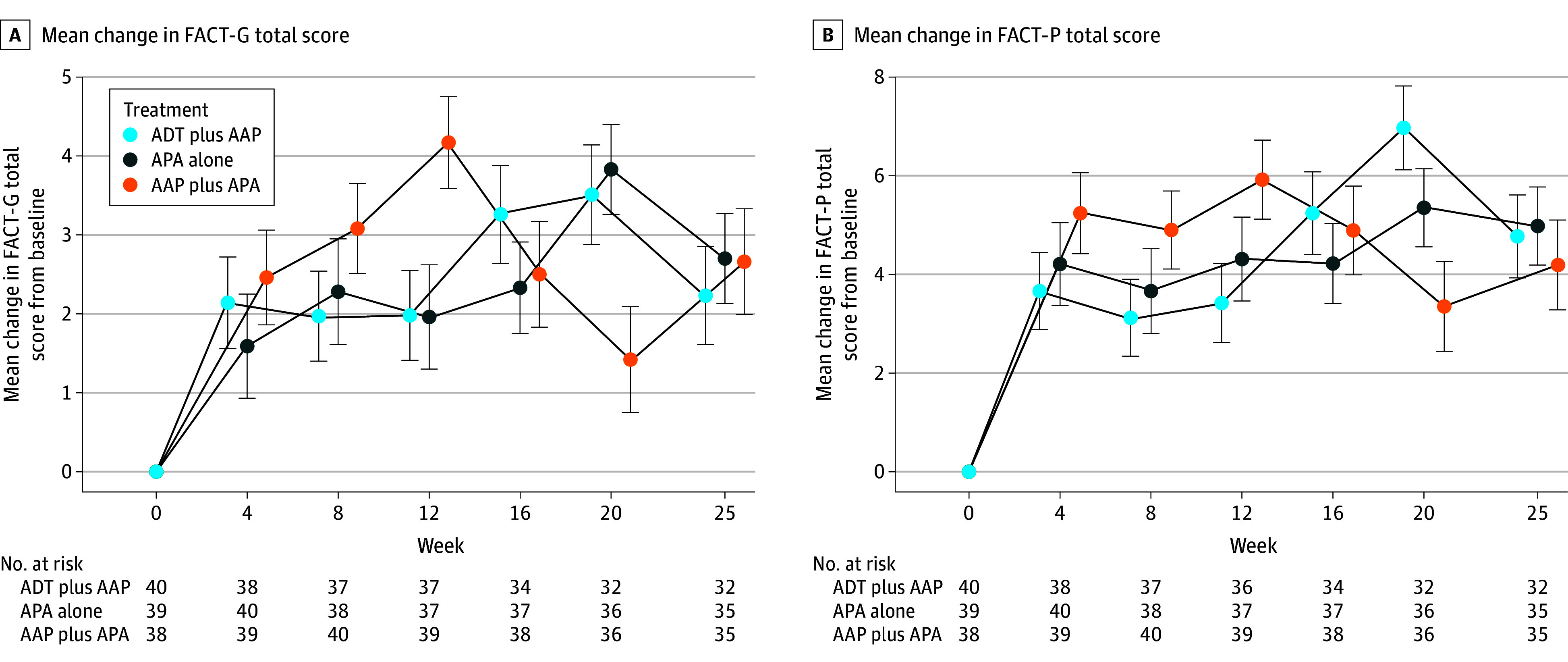
Mean Change in Functional Assessment of Cancer Therapy–General (FACT-G) Total and Functional Assessment of Cancer Therapy–Prostate (FACT-P) Scores From Baseline to Week 25 A, Mean change in FACT-G total score from baseline to week 25; theoretical score range, 0 to 108. A, Mean change in FACT-P total score from baseline to week 25; theoretical score range, 0 to 156. Error bars indicate 95% CIs. AAP indicates abiraterone acetate plus prednisone; ADT, androgen deprivation therapy; and APA, apalutamide.

Overall, HRQOL remained stable during the study period (eFigure 1 in Supplement 2). Mean FACT-P and FACT-G scores improved from baseline to week 25 within each arm (Figure 1). In the MMRM analysis when the 3 study arms were compared, a slight numeric difference favoring the APA alone arm was noted in the mean change in FACT-P and FACT-G scores from baseline to week 25; however, this change was not statistically significant (eTable 2 in Supplement 2). In the sensitivity analysis, there was no statistically significant difference between the arms (eTable 4 in Supplement 2).

#### FACT-P Subscales

**Figure 2.**
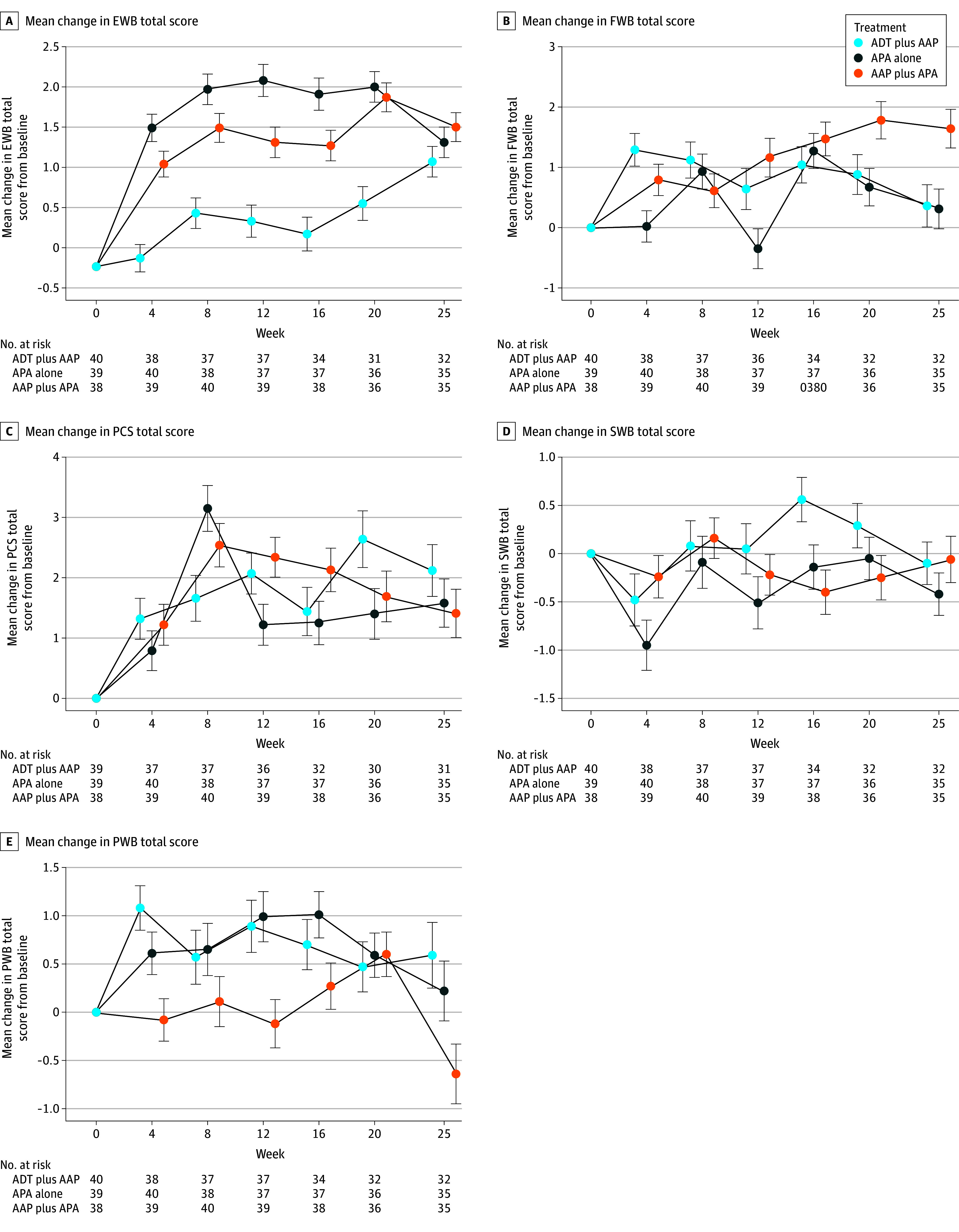
Mean Change in Functional Assessment of Cancer Therapy–Prostate (FACT-P) Subscales From Baseline to Week 25 A, Mean change in emotional well-being (EWB) subscale scores; theoretical score range, 0 to 24. B, Mean change in functional well-being (FWB) subscale scores; theoretical score range, 0 to 28. C, Mean change in prostate cancer subscale (PCS) scores; theoretical score range, 0 to 48. D, Mean change in social well-being (SWB) subscale scores; theoretical score range, 0 to 28. E, Mean change in physical well-being (PWB) subscale scores; theoretical score range, 0 to 28. Error bars indicate 95% CIs. AAP indicates abiraterone acetate plus prednisone; ADT, androgen deprivation therapy; and APA, apalutamide.

In the MMRM analysis the mean EWB, functional well-being, and prostate cancer subscale domain scores improved from baseline to week 25 within each arm. There was a slight within-group deterioration in social well-being scores from baseline to week 25 in all arms. Although physical well-being scores improved in the ADT plus AAP and APA alone arms, they deteriorated in the AAP plus APA arm (Figure 2; eFigure 2 in Supplement 2). When the 3 study arms were compared, there were no statistically significant differences in FACT-P subscale scores from baseline to week 25 (eTable 2 in Supplement 2). In the sensitivity analysis, there was no statistically significant difference between the arms (eTable 3 in Supplement 2).

#### Time to FACT-P and FACT-P Subscale Score Deterioration

**Figure 3.**
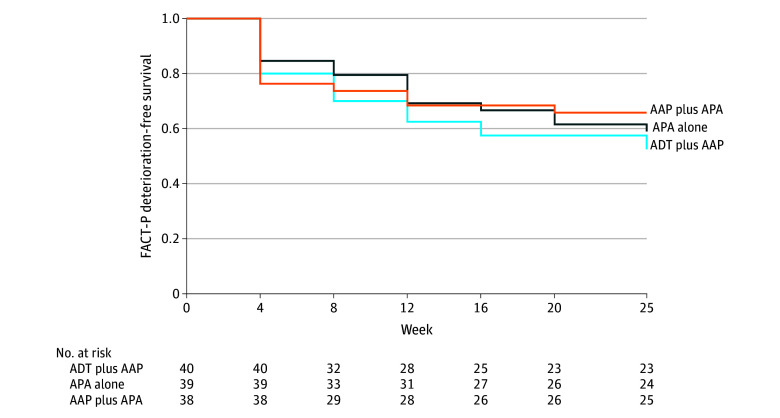
Time to Functional Assessment of Cancer Therapy–Prostate (FACT-P) Deterioration The FACT-P questionnaire was administered at baseline and every 4 weeks (±3 days) until week 25. AAP indicates abiraterone acetate plus prednisone; ADT, androgen deprivation therapy; and APA, apalutamide.

Time to FACT-P total score deterioration was not statistically significantly different when the 3 arms were compared (ADT plus AAP vs APA alone: hazard ratio [HR], 0.77 [95% CI, 0.40-1.46]; *P* = .41; ADT plus AAP vs AAP plus APA: HR, 0.72 [95% CI, 0.37-1.37]; *P* = .31) (Figure 3; Table 2). Time to deterioration of the EWB score was more favorable in the APA alone arm than the ADT plus AAP arm (reference arm: ADT plus AAP arm; APA alone: HR, 0.37 [95% CI, 0.15-0.85]; *P* = .02; AAP plus APA: HR, 0.56 [95% CI, 0.26-1.19]; *P* = .13) (Table 2).

**Table 2.** Time to First HRQOL Deterioration With ADT Plus AAP as Reference

Questionnaire	APA alone		AAP plus APA	
	HR (95% CI)^a^	*P* value	HR (95% CI)^a^	*P* value
Physical well-being	1.00 (0.50-2.00)	.99	1.21 (0.62-2.33)	.58
Functional well-being	1.32 (0.70-2.50)	.39	1.19 (0.61-2.30)	.60
Emotional well-being	0.37 (0.15-0.85)	.02	0.56 (0.26-1.19)	.13
Social well-being	0.97 (0.54-1.72)	.91	0.95 (0.53-1.69)	.85
Prostate cancer subscale	0.95 (0.50-1.79)	.87	1.11 (0.59-2.08)	.73
FACT-G total score	0.75 (0.40-1.41)	.37	0.68 (0.36-1.28)	.23
FACT-P total score	0.77 (0.40-1.46)	.41	0.72 (0.37-1.37)	.31

Abbreviations: AAP, abiraterone acetate plus prednisone; ADT, androgen deprivation therapy; APA, apalutamide; FACT-G, Functional Assessment of Cancer Therapy–General; FACT-P, Functional Assessment of Cancer Therapy–Prostate; HR, hazard ratio; HRQOL, health-related quality of life.

^a^ Reference: ADT plus AAP.

## Discussion

Androgen deprivation therapy remains the backbone therapy for advanced prostate cancer requiring systemic treatment, and ADT intensification with ARPIs and/or docetaxel are being increasingly used in this setting.^2,3,4,5,7,8,11^ Although this strategy is often becoming the standard of care, there are many clinical situations where a different approach may be chosen, such as for elderly patients with advanced CSPC, patients with low-volume and/or metachronous disease or with significant comorbidities, and patients who refuse continuous ADT.

Patient-reported outcomes assessed using multiple HRQOL questionnaires have been increasingly recognized and included as key secondary end points in multiple recent phase 3 clinical trials in advanced prostate cancer.^14^ For patients with castration-sensitive disease, HRQOL is particularly relevant due to symptom burden, a prolonged course of disease with potentially life-long treatments that induce testosterone suppression and its well-known consequences, and multiple treatment options with varying adverse event profiles.^19,20,21^

Several trials have assessed HRQOL in CSPC treated with ARPIs, with or without ADT. Although studies have demonstrated improved HRQOL with the addition of ARPIs to ADT,^22,23^ results varied across trials using different ARPIs (apalutamide, enzalutamide) and assessment tools (FACT-P, BPI-SF [Brief Pain Inventory–Short Form], EORTC QLQ-C30 [European Organisation for Research and Treatment of Cancer Core Quality of Life Questionnaire], EORTC QLQ-PR25 [European Organisation for Research and Treatment of Cancer Quality of Life Questionnaire for Patients With Prostate Cancer], and EQ-5D-5L [EuroQol 5 Dimensions-5 Level]). Direct HRQOL comparisons are challenging due to variations in study populations and outcome measures.^22,23^

Even though several studies have assessed HRQOL in CSPC, data on alternative treatments are scarce. As previously discussed, some strategies may minimize ADT-associated symptoms, including sexual dysfunction, and eventually improve HRQOL. A phase 2 study with single-agent enzalutamide included 67 patients with indication of systemic therapy for CSPC and HRQOL was evaluated (EORTC QLQ-C30 and QLQ-PR25 questionnaires).^24,25^ Although global quality of life was maintained during treatment with enzalutamide, an increase in fatigue and treatment-related symptom scales and moderate deteriorations of sexual activity domain were observed vs the baseline scores.^24^ Because this was a single-arm study, it was not possible to compare the HRQOL of enzalutamide monotherapy with that of ADT. Recently, 2 studies evaluated ARPI monotherapy as a potential treatment strategy for CSPC, which led to approval of enzalutamide use for high-risk biochemical recurrence.^26,27^ A phase 3 randomized clinical trial (EMBARK trial) with patients with high-risk biochemical recurrence to ADT alone assessed enzalutamide alone or ADT plus enzalutamide.^26^ Both the ADT plus enzalutamide and enzalutamide monotherapy arms improved metastasis-free survival compared with ADT alone. There has been significant interest in evaluating the adverse effect profile and quality of life of patients treated with ARPI monotherapy in the EMBARK trial, because this strategy is associated with increased levels of testosterone and might mitigate ADT-associated symptoms such as sarcopenia and sexual dysfunction. In the EMBARK study, the baseline quality of life scores indicated high HRQOL, and all treatment arms were associated with relatively stable quality of life throughout the study. Nevertheless, in terms of sexual activity, all treatment arms were associated with moderate deterioration in this aspect, with a slightly longer time to sexual deterioration in the enzalutamide monotherapy arm. Most patients described low sexual activity scores at baseline, which may represent an important limitation to this analysis. Moreover, the toxicity profile was different between enzalutamide and ADT, with higher rates of hot flushes in the ADT arm and higher rates of gynecomastia and cognitive impairment in the enzalutamide arm.

Other important data regarding quality of life among patients treated with ARPI monotherapy come from the EORTC-GUGG 1532 study comparing darolutamide alone vs ADT in 61 patients with advanced CSPC.^27^ Overall, no meaningful changes in quality of life were demonstrated in that study, possibly due to small sample size and limited power for these analyses. A nonsignificant change favoring darolutamide monotherapy was seen in the hormone therapy symptom scale and the sexual functioning domain of the EORTC QLQ-PR25 questionnaire.

The LACOG0415 study evaluated strategies with either APA alone or AAP plus APA for patients with advanced CSPC requiring systemic therapy, with HRQOL assessed as a key secondary end point. In this study, the FACT-P questionnaire was used to assess HRQOL, completed by patients enrolled in the trial every 4 weeks up to week 25. After week 25, patients who were benefiting from the study therapies continued in the extension phase of the trial, but no further HRQOL assessment was planned. The LACOG0415 trial demonstrated high rates of PSA decrease within all 3 study arms, including the APA and APA plus AAP arms. One interesting result in the previous analysis was the change in the total testosterone level during treatment, with a significant increase in the total testosterone level in the APA alone arm and reaching castration levels in the ADT plus AAP and APA plus AAP arms (median total testosterone: ADT plus AAP arm, 9.0 ng/dL; APA plus AAP arm, 30.4 ng/dL; and APA alone arm, 1022 ng/dL).^15^ Other relevant reported findings were the TRAEs data, with lower rates of hot flushes and hypertension in the APA alone arm. On the other hand, patients in the APA alone arm experienced a higher rate of gynecomastia (54.8%) and breast pain (14.3%), known TRAEs of antiandrogen monotherapy, compared with the other 2 groups.^15,25^ Despite these results, including testosterone kinetics and adverse events within study arms, the HRQOL assessed through the FACT-P questionnaire did not demonstrate a clinically meaningful and significant difference among the 3 arms. The only statistically significant difference was in time to deterioration of the EWB score between the APA alone and ADT plus AAP arms. However, in the AAP plus APA arm, the small sample size may have prevented detection of statistical significance. All treatments were very well tolerated. The low rate of adverse events, the high PSA decrease rate, and the short follow-up of the HRQOL questionnaires may explain the stability during the trial and the absence of differences among arms.

Despite having been validated for HRQOL assessment among patients with prostate cancer in different scenarios, the FACT-P tool may not capture individual changes in some aspects of patient’s lives, especially with short-term assessments and among cohorts with low symptom burden. Important symptoms related to prostate cancer therapies are not fully captured in the FACT-P, lacking detailed questions to assess loss of libido, sarcopenia, and even sexual function. This may limit the ability to demonstrate meaningful improvements in specific symptoms and to discriminate HRQOL between ADT and ARPI monotherapy. Ideally, the FACT-P questionnaire should be complemented with other validated tools to increase sensitivity to detect clinically meaningful changes in patients’ HRQOL, as well as, with longer follow-up, to capture some long-term effects of ADT.^14,18,28^ The increase of testosterone in the APA arm was not associated with a benefit in HRQOL as evaluated by the FACT-P questionnaire.

### Limitations

Our study had some limitations, including the relatively small number of patients treated with open-label treatments, the inclusion of a heterogeneous group of asymptomatic or mildly symptomatic patients with advanced CSPC requiring systemic therapy (ie, locally advanced disease, high-risk biochemical relapse, and metastatic disease), the lack of long-term assessments of HRQOL, and the use of a single HRQOL questionnaire (FACT-P) rather than including other scales (eg, IIEF-5 [International Index of Erectile Function-5] and IIEF-15 [International Index of Erectile Function-15] to capture improvement in sexual function). Therefore, these results should be considered hypothesis generating.

## Conclusions

In this secondary analysis of a randomized clinical trial, HRQOL remained stable during treatment with ADT plus AAP, APA alone, and AAP plus APA for patients with advanced CSPC. There was no statistically significant difference between APA alone and AAP plus APA in terms of HRQOL compared with ADT plus AAP. The difference detected in time to deterioration of the EWB score between APA alone and ADT plus AAP should be assessed in future research. Larger studies with longer follow-up and more specific questionnaires are needed to further evaluate HRQOL with these treatment strategies.

## Notes

Supplement 1.
Trial Protocol and Statistical Analysis Plan

Supplement 2. **eFigure 1.** Mean FACT-P (A) and FACT-G (B) Total Scores at Baseline and During Treatment in the 3 Arms **eFigure 2.** Mean FACT-P Subscale Scores at Baseline and During Treatment in the 3 Arms **eTable 1.** Baseline Characteristics **eTable 2.** Health-Related Quality of Life (HRQoL) Scores **eTable 3.** Treatment-Related Adverse Events **eTable 4.** Adjusted Mean Changes in HRQoL Scores From Baseline to Week 25 (MMRM and Sensitivity Analysis) **eAppendix 1.** Eligibility Criteria **eFigure 3.** Trial Flow Diagram **eAppendix 2.** Randomization Procedures

Supplement 3.
Data Sharing Statement
