# Supplementary material for: Androgen Receptor Pathway Inhibitor Therapy for Advanced Prostate Cancer: Secondary Analysis of a Randomized Clinical Trial
Source: JAMA Netw Open. 2025 Jan 13;8(1):e2454253. doi: 10.1001/jamanetworkopen.2024.54253 (PMC11731179; doi:10.1001/jamanetworkopen.2024.54253)
Supplement: Supplement 1. — Trial Protocol and Statistical Analysis Plan [file jamanetwopen-e2454253-s001.pdf]

LACOG
LATIN AMERICAN
COOPERATIVE
ONCOLOGY GROUP

Phase II randomized study of Abiraterone acetate plus ADT versus APALUTAMIDE versus Abiraterone and APALUTAMIDE in patients with advanced prostate cancer with non-castrate testosterone levels.

Phase II randomized study of Abiraterone acetate plus ADT versus APALUTAMIDE versus Abiraterone and APALUTAMIDE in patients with advanced prostate cancer with non-castrate testosterone levels.

LACOG 0415

Investigational drug: Abiraterone and APALUTAMIDE

Coordinating Group: LACOG Genitourinary Cancer Group

Study Principal Investigator: Dr. Fernando Cotait Maluf

Protocol Version	Data of LACOG approval	Amendments references	
		No.	
Outline	26 Mar 2015	---	
1.0	03 Mar 2016	---	
2.0	09 Nov 2018	1	
3.0	30 Oct 2020	2	

PROTOCOL AMENDMENT, VERSION 3

Study Scientific Committee

Investigator Oren Smaletz, Hospital Albert Einstein, São Paulo, Brazil

**Investigator**	Fábio Schutz, Beneficência Portuguesa, São Paulo, Brazil
**Investigator**	Vinicius Carreira, Clínica AMO, Salvador, Brazil
**Investigator**	André Fay, PUCRS, Porto Alegre, Brazil
**Investigator**	Daniel Herchenhorn, INCA, Rio de Janeiro, Brazil
**Janssen**	Rosemarie Gidekel, LATAM MAF Oncology director

Contacts

**Principal Investigator:**	Dr. Fernando Cotait Maluf<br>Centro Oncológico Antônio Ermírio de Moraes – Beneficência Portuguesa de São Paulo<br>Rua Martiniano de Carvalho 965<br>Zip Code: 01321-001<br>Phone: +55 11 3505 6612<br>Fax: +55 11 3505 6638<br>Email: maluffc@uol.com.br
**Medical consultant**	Gustavo Werutsky<br>Phone: +55 51 33845334<br>Fax: +55 51 3384 1855<br>Email: gustavo.werutsky@lacog.org.br
**LACOG Project Manager:**	Laura Voelcker<br>Phone: +55 51 33845334<br>Fax: +55 51 33841855<br>Email: laura.voelcker@lacog.org.br
**LACOG Data Manager**	Daniela Muller Kurban Phone: +55 51 33845334<br>Fax: +55 51 33841855<br>Email: daniela.kurban@lacog.org.br
**LACOG Statistician:**	Rafaela Gomes de Jesus<br>Phone: +55 51 33845334<br>Fax: +55 51 33841855<br>Email: rafaela.gomes@lacog.org.br
**Janssen's Study Responsible Physician:**	Rosemarie Gidekel<br>Email: rgidekel@its.jnj.com

Protocol LACOG 0415 – Signature page

Sponsor:

Carlos Henrique Escosteguy Barrios	Date

Executive Director - Latin American Cooperative Oncology Group
Av. Ipiranga, 6681 – Prédio 99A – Sala 806
CEP 90619-900 Porto Alegre-RS
Phone: +55 51 3384 5334
Fax: +55 51 3384 1855
Email: lacog@lacog.org.br
Website: http://www.lacog.org.br

Study Principal Investigator:

Fernando Cotait Maluf	Date

Dr. Fernando Cotait Maluf
Centro Oncológico Antônio Ermírio de Moraes – Beneficência Portuguesa de São Paulo
Rua Martiniano de Carvalho 965
Zip Code: 01321-001
Phone: +55 11 3505 6612
Fax: +55 11 3505 6638
Email: maluffc@uol.com.br

Site Investigator:

Investigator's name	Date

Index

Study Scientific Committee **1**
Contacts **2**
Protocol LACOG 0415 – Signature page **3**
Sample Size **18**
1 Background **23**
2 Study rationale/purpose **25**
3 Objectives **26**
3.1 Primary objective 26
3.2 Secondary objectives 26
4 Study design **27**
5 Population **27**
5.1 Inclusion criteria 27
5.2 Exclusion criteria 28
5.3 Prohibitions and Restrictions 30
6 Study drug information **31**
6.1 Investigational and control drugs 31
6.2 Treatment Allocation 32
6.3 APALUTAMIDE 32
6.4 Abiraterone acetate 33
6.5 ADT 33
6.6 Drug Accountability 34
7 Dosage and administration **34**
7.1 Planned dose 34
7.2 Dosing modifications and Management of Toxicity 35
7.2.1 Abiraterone acetate 35
7.2.2 APALUTAMIDE 36
7.3 Treatment Compliance 37
7.4 Concomitant Treatment 37
7.4.1 Prestudy and Concomitant Therapy 38
7.4.2 Permitted Supportive Care and Interventions During Study 38

7.4.3 Special Concomitant Therapy ... 38
7.4.4 Prohibited Medications ... 40
7.5 Study drug discontinuation ... 41
7.6 Premature patient withdrawal ... 43
7.6.1 End of treatment ... 43
7.6.2 Study evaluation completion ... 43
8 Visit schedule and assessments ... 44
8.1 Information to be collected on screening failures ... 48
8.2 Patient demographics/other baseline characteristics ... 48
8.3 Treatments ... 48
8.4 Efficacy ... 48
8.5 Safety ... 48
8.5.1 Physical examination ... 48
8.5.2 Vital signs ... 48
8.5.3 Performance status ... 49
8.5.4 Laboratory evaluations ... 49
8.5.5 Radiological examinations ... 49
8.5.6 Cardiac assessments ... 49
8.6 Patient-reported outcomes ... 49
9 Study completion ... 49
10 Safety monitoring_ ... 50
10.1 Adverse events ... 50
10.1.1 Adverse Events Reporting ... 50
10.1.2 Adverse Event Definitions and Classifications ... 50
10.1.3 Attribution Definitions ... 52
10.2 Special Reporting Situations ... 52
10.3 Procedures ... 53
10.3.1 All Adverse Events ... 53
10.3.2 Serious Adverse Events ... 54
10.3.3 Pregnancy ... 55
10.3.4 Contacting Sponsor Regarding Safety ... 55

10.4.1 Procedures....56
10.4.2 Contacting Sponsor Regarding Product Quality....56
11 Data review and data management....56
11.1 Site monitoring....56
11.2 Data collection....57
11.3 Database management and quality control....58
12 Statistical methods and data analysis....58
12.1 Sample Size....58
12.2 Populations for analysis....59
12.3 Efficacy analyses....60
12.3.1 Analysis of primary endpoint....60
12.3.2 Analysis of secondary endpoints....60
12.3.3 Patient-reported outcomes....61
12.4 Safety analyses....61
12.4.1 Adverse events (AE)....61
12.4.2 Laboratory abnormalities....62
12.4.3 Other safety data....62
12.4.4 Vital Signs....62
12.5 Resource utilization....62
12.6 Treatment compliance....62
13 Translational Research....62
14 Study-specific materials....66
15 Ethical aspects....66
15.1 Study-Specific Design Considerations....66
15.2 Regulatory Ethics Compliance....66
15.2.1 Investigator Responsibilities....66
15.2.2 Independent Ethics Committee or Institutional Review Board....67
15.2.3 Informed Consent....68
15.2.4 Privacy of Personal Data....69
16 Administrative requirements....69
16.1 Protocol Amendments....69
16.2 Regulatory Documentation....70

16.2.1 Regulatory Approval/Notification 70
16.2.2 Required Prestudy Documentation 70
16.2.3 Record Retention 71
16.3 Study Completion/Termination 71
16.3.1 Study Completion 71
16.3.2 Study Termination 71
16.3.3 On-Site Audits 72
16.4 Use of Information and Publication 72
17 Publication policy 72
18 References 73

OVERALL RATIONALE FOR REVISED PROTOCOL 03

In addition to modifying existing language for better clarity, the protocol revision clarifies the analyses population for each endpoint and adds the following endpoints overall survival and time to next treatment.

PROTOCOL AMENDMENT, VERSION 3:<br>SUMMARY OF CHANGES		
Section Number & Title	Description of Change	Brief Rationale
Protocol summary, Endpoints, Secondary objectives	Inclusion of two secondary objectives:<br>-2-year overall survival rate<br>-Time to next treatment	Overall survival and time to next treatment are important endpoints to be evaluated.
Protocol summary, Endpoints, Secondary objectives	Excluded secondary endpoint comparisons	These assessments are no longer planned.
Protocol summary, Endpoints, Secondary objectives	Updated sentence “radiographic progression free-survival at week 25 among the three arms” to “radiographic progression rate at week 25 among the three arms”	Changed to radiographic progression rate analysis since image evaluation is planned to be perform at baseline and at week 25.
Protocol summary, Statistical methods and data analysis, sample size	Deleted first phrase of paragraphs 4. The following paragraph was added: *“Intention to treat (ITT) population and modified ITT (mITT) population analyses were performed for the endpoints. For the primary endpoint an additional sensitivity analysis was performed and missing PSA data at week 25 were labeled as failures.”*	A sensitivity analyses was included to guarantee the robustness of the findings based on primary analyses,

Protocol summary, Statistical methods and data analysis, Populations for analysis	Deleted of paragraphs 1 and 2. Sentences inserted to clarify the analyses population for each endpoint.	To clarify the analyses population for each endpoint.
Protocol summary, Statistical methods and data analysis, Analysis of primary endpoint	Sentence inserted to clarify the analyses population for the primary endpoint.	To clarify the analyses population for the primary endpoint
Protocol summary, Statistical methods and data analysis, Analysis of secondary endpoint	"bone density" is deleted.	The deleted texts were inadvertent included in the protocol.
Protocol summary, Statistical methods and data analysis, Analysis of secondary endpoint	Deleted of paragraph 3. Sentence inserted to clarify the analyses of Radiographic progression rate at week 25.	Updated to align with the definition of updated secondary endpoint: Radiographic progression rate at week 25
Objectives, secondary objectives	Updated sentence "radiographic progression free-survival at week 25 among the three arms" to "radiographic progression rate at week 25 among the three arms"	Updated to align with the definition of updated secondary endpoint: Radiographic progression rate at week 25
Objectives, secondary objectives	Inclusion of two secondary objectives:<br>-2-year overall survival rate<br>-Time to next treatment	Overall survival and time to next treatment are important endpoints to be evaluated.
Objectives, secondary objectives	Excluded secondary endpoint comparisons	These assessments are no longer planned.

Safety, radiological examination	"bone density" is deleted.	The deleted texts were inadvertent included in the protocol.
Data review and data management	Phrases regarding remote monitoring were added.	To clarify and add information.
Statistical methods and data analysis, sample size	Deleted first phrase of paragraphs 4. The following paragraph was added: *"Intention to treat (ITT) population and modified ITT (mITT) population analyses were performed for the endpoints. For the primary endpoint an additional sensitivity analysis was performed and missing PSA data at week 25 were labeled as failures."*	A sensitivity analyses was included to guarantee the robustness of the findings based on primary analyses,
Statistical methods and data analysis, Populations for analysis	Deleted of paragraphs 1,2, and 3. Sentences inserted to clarify the analyses population for each endpoint.	To clarify the analyses population for each endpoint.
Statistical methods and data analysis, Analysis of secondary endpoint	Deleted of paragraph 3. Sentence inserted to clarify the analyses of Radiographic progression rate at week 25.	Updated to align with the definition of updated secondary endpoint: Radiographic progression rate at week 25
Safety analysis, adverse events (AEs)	Sentence inserted: "Treatment-related adverse events are AEs that occur or worsen on or after first dose of study drug through 28 days	Adds additional details on Safety analysis

	after the last dose of study drug and were considered related to study drug. Multiple occurrences of the same event were counted once at the maximum severity.”	
Translational research	Translational research chapter was updated.	Adds additional translational analyses to better comprehend the final study results and the safety profile.

Protocol Summary

**Study title**	Phase II randomized study of Abiraterone acetate plus ADT versus APALUTAMIDE versus Abiraterone and APALUTAMIDE in patients with advanced prostate cancer with non-castrate testosterone levels.
**Purpose/rational**	Based on the current guidelines, ADT alone or combined with is antiandrogens are considered the appropriate active therapy for the patient population planned for this study. Recent data showed that chemotherapy also benefit patients in this setting. Even though, there is a clear unmet medical need for alternative treatment option in metastatic hormone sensitive prostate cancer (mHSPC). Treatments that can delay disease progression, and are associated with less comorbidities would be of significant clinical benefit in this patient population. Our study is designed to assess the efficacy and safety of abiraterone plus APALUTAMIDE (a second-generation antiandrogen) or APALUTAMIDE alone without castration side effects and the other arm a combination of ADT and abiraterone; this last arm is based on LATTITUDE trial, which showed a survival benefit of adding abiraterone to castration in this setting of patients. Abiraterone had already showed clinical benefit in CRPC patients without prior chemotherapy.
**Objectives**	We aim to assess the activity, safety and patients reported outcome of ADT plus abiraterone, abiraterone plus APALUTAMIDE (a second-generation antiandrogen) or APALUTAMIDE alone in hormone naïve locally advanced or metastatic prostate cancer which ADT was indicated.

**Endpoints**	**Primary objective**<br>The primary objective is to evalute the proportion of patients who achieves an undetectable PSA level, defined as ≤ 0.2 ng/mL at week 25 week in each of three arms.<br><br>**Secondary objectives**<br>- To determine ~~and compare~~ PSA progression rate at week 25 (PCWG3 criteria)<br><br>- To determine ~~and compare~~ PSA response of 50 and 80% at week 25<br><br>- To determine maximum PSA declines and overall PSA change from baseline up to week 25 and up to week 53;<br><br>- To determine the radiographic progression rate at week 25 among the three arms;<br><br>- To determine ~~and compare~~ hormonal levels during treatment;<br>- To determine ~~and compare~~ the safety profile;<br><br>- To determine and compare the time to pain progression assessed by BPI-SF and opioid use;<br><br>- To determine and compare the quality of life assessed by FACT-P;<br><br>- To determine time to prostate cancer castration resistance;<br><br>- Metastatasis free survival (on non-metastatic patients at inclusion).<br><br>- Overall survival at 2 years.<br><br>- Time to next treatment.
**Study Design**	This is a phase II, open label, randomized trial evaluating the efficacy of abiraterone acetate 1000mg PO QD plus prednisone 5mg PO BID and Androgen Deprivation Therapy (ADT) versus APALUTAMIDE 240mg PO versus the combination of abiraterone acetate 1000mg PO QD plus prednisone 5mg PO BID and APALUTAMIDE (without ADT), both at the standard doses, in patients with advanced or metastatic prostate cancer with non-castrate testosterone levels. Efficacy will be measured primarily by assessing percentage of patients who achieves an undetectable PSA level defined as ≤ 0.2 ng/mL at week 25. A second efficacy analysis will be done at week 53. The total study period is 2 years

	including patient treatment and outcome data collection. Patients will be treated until objective or clinical disease progression or occurrence of an unacceptable toxicity. Patients are allowed to continue study treatment beyond the 25-week assessment (extension phase) at the discretion of the investigator.
**Eligibility criteria**	**Inclusion criteria**<br>1. Histologically confirmed prostate adenocarcinoma;<br>2. Patients with indication to start treatment with ADT in one of the following settings:<br>a. Biochemical relapse after definitive treatment (surgery and/or radiotherapy): PSA ≥ 4 ng/ml and doubling time less than 10 months, or PSA ≥ 20 ng/ml;<br>b. Newly diagnosed Prostate Cancer: locally advanced – T_{any} N+ M0 (not candidate to definitive treatment with surgery or radiotherapy) or metastatic – T_{any} N_{any} M+ and PSA ≥ 2ng/mL;<br>3. Patient is asymptomatic to moderately symptomatic regarding bone symptoms, i.e., no need for palliative radiation or radionuclide therapy;<br>4. Complete staging process (performed as per routine), meaning, thorax, abdomen and pelvis TC and bone scan, performed before consent and that do not exceed 8 weeks from the date of randomization;<br>5. Non-castration level of testosterone ≥ 230ng/dL (≥ 8 nmol/L);<br>6. ECOG performance status of 0 to 2;<br>7. Adequate hematologic, hepatic and renal function:<br>a. hemoglobin > 10 g/dL, neutrophils > 1.5×10^9 / L, platelets> 100×10^9 / L;<br>b. total bilirubin < 1.5x upper limit of normal (ULN); alanine (ALT) and aspartate (AST) aminotransferase < 2.5 x ULN;<br>c. serum creatitine < 1.5x ULN; potassium > 3.5 mM;<br>8. Written informed consent obtained prior to any study procedure;<br>9. Men age 18 years and older;<br>10. Agrees to use a condom and another effective method of birth control if he is having sex with a woman of childbearing potential or agrees to use a condom if he is having sex with a woman who is pregnant.
	**Exclusion criteria**<br>1. Prostate adenocarcinoma with neuroendocrine differentiation or small cell histology;<br>2. Use of hormonal therapy or chemotherapy prior to randomization. Except in case of hormone therapy for localised disease that have been completed for at least 12 months previously. It can have been given as adjuvant or neoadjuvant therapy;<br>3. Prior radiation therapy for a primary tumour within the 3 months before enrollment or for the treatment of metastases;<br>4. Known or suspected brain or skull metastases or leptomeningeal

	metastatic disease;<br>5. Any concurrent severe and/or uncontrolled medical conditions which could compromise participation in the study;<br>6. Administration of an investigational therapeutic or invasive surgical procedure within 28 days of Cycle 1 Day 1 or currently enrolled in an investigational study;<br>7. Active or symptomatic viral hepatitis or chronic liver disease; ascites or bleeding disorders secondary to hepatic dysfunction;<br>8. Current or prior treatment with anti-epileptic medications for the treatment of seizures;<br>9. Impaired cardiac function, including any of the following:<br>a. Uncontrolled hypertension (systolic blood pressure ≥160 mmHg or diastolic BP ≥95 mmHg);<br>b. Clinically significant heart disease as evidenced by myocardial infarction, or arterial thrombotic events or history of cardiac failure in the past 6 months, severe or unstable angina, or New York Heart Association (NYHA) Class II-IV heart disease;<br>c. Existing atrial fibrillation with or without pharmacotherapy. Other cardiac arrhythmia requiring pharmacotherapy;<br>10. History of seizure or condition that may predispose to seizure (including, but not limited to prior stroke, transient ischemic attack or loss of consciousness ≤1 year prior to randomization; brain arteriovenous malformation; or intracranial masses such as schwannomas and meningiomas that are causing edema or mass effect);<br>11. Specific underlying conditions for oral agents. For example: impairment of gastrointestinal (GI) function or GI disease that may significantly alter the absorption of abiraterone or APALUTAMIDE (e.g., ulcerative diseases, uncontrolled nausea, vomiting, diarrhea, malabsorption syndrome, or small bowel resection)<br>12. General excluded medications (e.g., relevant to cytochrome P450 interactions)<br>a. Use of prescription drugs within 14 days prior to dosing or over-the-counter (OTC) medication within 7 days prior to dosing;<br>b. Consumption of grapefruit product or St John's wort within 7 days prior to dosing;<br>c. G-CSF, GM-CSF, erythropoietin, etc;<br>d. Coumadin;<br>e. Drugs which may cause QT prolongation;<br>f. Known sensitivity to drugs or metabolites from similar classes;<br>g. Known or suspected contraindications or hypersensitivity to APALUTAMIDE, bicalutamide or GnRH agonists or any of the components of the formulations;13. Any condition or situation which, in the opinion of the investigator, would put the subject at risk, may confound study results, or interfere with the subject's participation in this study;

	14. Surgical castration prior to study entry;<br>15. Had a prior malignancy. Adequately treated basal cell or squamous cell carcinoma of skin or superficial bladder cancer that has not spread behind the connective tissue layer (i.e., pTis, pTa, and pT1) is allowed, as well as any other cancer for which treatment has been completed 5 years before randomization and from which the subject has been disease-free.
**Investigational and control drugs**	Abiraterone acetate, prednisone, ADT (Goserelin) and APALUTAMIDE.
**Dose, regimen, treatment cycle**	This is a three-arm study consisted of:<br>**Arm 1**: Abiraterone acetate + Prednisone + ADT (Goserelin).<br>• Abiraterone administered at a single 1000 mg daily oral dose (4 x 250-mg tablets)<br>• Prednisone administered at a 5 mg twice daily oral dose<br>• Goserelin administered as subcutaneous injections of 10.8mg every 3 months<br><br>**Arm 2:** APALUTAMIDE monotherapy<br>• APALUTAMIDE administered at a single 240 mg daily oral dose (4 x 60 mg tablets)<br><br>**Arm 3:** Abiraterone acetate + Prednisone + APALUTAMIDE<br>• Abiraterone administered at a single 1000 mg daily oral dose (4 x 250 mg tablets)<br>• Prednisone administered at a 5 mg twice daily oral dose<br>• APALUTAMIDE administered at a single 240 mg daily oral dose (4 x 60 mg tablets)<br><br>Per protocol study treatment is planned until week-25. Patients will be treated until objective or clinical disease progression or occurrence of an unacceptable toxicity. Patients are allowed to continue study treatment beyond the week 25 (extension phase) at the discretion of the investigator.<br><br>Patients will be discontinued from the planned study treatment due to progression (radiographic per RECIST 1.1 and/or symptomatic +/- biochemical according to PCWG3 criteria), adverse event or patient withdrawal.
**Supply, preparation and administration**	Subjects will be randomly assigned to the each active arm in a 1:1:1 ratio after the investigator has verified that all eligibility criteria have been met. The randomization will be balanced by using randomly permuted blocks. Randomization will take place across all study sites using a centralized Interactive Web Response System (IWRS). Subjects will be stratified by performance status (ECOG 0-1 vs 2) and metastatic disease (yes vs. no).

	At study inclusion (right after informed consent has been obtained), the eCRF will assign a unique subject identification number to each subject. The subject's identification number will be used on all study-related documents including electronic case report forms (eCRFs). A treatment number will also be assigned to each subject.<br><br>This treatment number is the link between a subject's eCRF and treatment group assignment. Subject identification numbers will not be reused. Subjects withdrawn from the study will not be replaced. All subjects must commence treatment within 72 hours (3 calendar days) date, in which an official statement is sent to the research site from LACOG (via e-mail).
**Visit schedule and assessments**	Please refer to page section 8 "Visit schedule and assessments table on pages 37, 38 and 39.
**Efficacy assessment(s)**	All patients will have their lesions followed by computed tomography and bone scan, according to RECIST criteria 1.1 [26] PSA test will be collected at screening within before 7 days from randomization, cycle 2 and monthly until PSA confirmation visit.
**Special safety assessment(s)**	An Adverse Event (AE) is defined by the FDA and by NCI in NCI Guidelines for Investigators: Adverse Event Reporting Requirements for DCTD (CTEP and CIP) and DCP INDs and IDEs, as any untoward medical occurrence associated with the use of a drug in humans, whether or not considered drug related. Therefore an AE can be ANY unfavorable and unintended sign (including an abnormal laboratory finding), symptom or disease temporally associated with the use of a medicinal (investigational) product whether or not considered related to the medicinal (investigational) product (attribution of unrelated, unlikely, possible, probable, or definite). An AE may consist of the following: 1) A new event which was not pre-existing at initial study drug administration. 2) A pre-existing event which recurs with increased intensity or increased frequency subsequent to study drug administration. 3) An event which is present at the time of study drug administration which is exacerbated following initial study drug administration. A persistent AE is one that extends continuously, without resolution between treatment cycles/courses. When an AE meets criteria for expedited reporting as an SAE, it must be reported only once unless the grade becomes more severe in the same or subsequent cycle/course. A recurrent AE is one that occurs and resolves during a cycle/course of therapy and then reoccurs in a later cycle/course. Once reported, a recurring AE requires expedited reporting if the grade increases from the grade originally reported or if hospitalization is associated with the recurrence. All adverse events will be categorized using NCI's CTCAE v4.

	A Serious Adverse Event (SAE) is defined by FDA and NCI as any adverse drug event (experience) occurring at any dose that in the opinion of either the investigator or sponsor results in any of the following outcomes: 1) Death 2) Life-threatening adverse drug experience 3) Inpatient hospitalization or prolongation of existing hospitalization (for > 24 hours) 4) Persistent or significant incapacity or substantial disruption of the ability to conduct normal life functions 5) Congenital anomaly/birth defect 6) Important Medical Event (IME) that may not result in death, be life threatening, or require hospitalization may be considered a serious adverse drug experience when, based upon medical judgment, it may jeopardize the patient or subject and may require medical or surgical intervention to prevent one of the outcomes listed in this definition Although the precise reporting requirements vary, these definitions apply in general to investigational agents, commercial agents, or a combination of investigational and commercial agents. It is important to remember that all SAEs are adverse events, but not all adverse events are SAEs, and need to be expeditiously reported only if they meet the guidelines for expedited reporting in Section 16 of the protocol. The definition of an SAE does include myelosuppression with a drug known to cause that adverse event if it is clearly the major factor leading to a death.
**Patient reported outcomes**	Patient-reported Outcomes (adapted BPI-SF and FACT-P questionnaires) will be collected.
**Study completion**	The patients who are benefiting from the study treatment, regardless of the treatment arm they have been assigned to, at week 25 are allowed to continue receiving this medication in an extension phase. These patients will be followed at 2 different times: 12 and 24 months after study treatment initiation. Biochemical and radiological progression and survival status data will be collected from the medical chart (Cycle 1 Day 1).
**Statistical methods and data analysis**	All statistical analyses specified in this protocol will be conducted using SAS version 9.4, and a significance level of 5%.<br><br>**Sample Size**<br>For the primary endpoint (PSA below 0.2ng/mL at week 25), and using Fleming one-stage method, a sample size of 38 participants per arm would allow 80% power to reject a PSA undetectable rate (defined as ≤ 0.2 ng/mL) of 45% or less at a 5% significance level, with an expected PSA response rate for each of the three arms of about 65%.[25,27] Allowing a 10% dropout, we planned to enroll 42 participants per arm. In total the study will enroll 126 patients.<br><br>Eligible patients will be stratified in randomization according to performance status (ECOG 0-1 vs 2) and metastatic disease (yes vs. no).

Probably the study will have a power lower than 80% in the most of secondary analyses. For that reason, a power calculation will be performed for each secondary association, especially for those with p-value < 0.05.

~~‘~~No interim analysis for futility is planned.

New amendment text (refers strikethrough sentence): “Intention to treat (ITT) population and modified ITT (mITT) population analyses were performed for the endpoints. For the primary endpoint an additonal sensitivity analysis was performed and missing PSA data at week 25 were labeled as failures.”

Populations for analysis

~~ITT Population: The ITT population includes all randomized subjects classified according to their assigned treatment group, regardless of the actual treatment received. Subject disposition and efficacy analyses will be performed on data from the ITT population.~~

~~Safety Population: The safety population includes all subjects who received at least 1 dose of study drug.~~

~~Patient-reported Outcomes Population [PRO]: The PRO population includes randomized subjects who have completed at least the baseline assessment of BPI-SF and FACT-P questionnaires.~~

New protocol amendment text (refers strikethrough sentence):

“ITT Population: *The ITT population includes all randomized subjects classified according to their assigned treatment group, regardless of the actual treatment received.*

- *Baseline patient characteristics*
- *Maximum PSA declines and overall PSA change from baseline up to week 25*
- *Hormonal levels during treatment*
- 2-year overall survival rate
- *Time to next treatment*

Modified ITT Population*: The modified ITT population includes only randomized subjects with evaluable PSA at week 25.*

- *Primary endpoint: proportion of patients who achieve an undetectable PSA level, defined as ≤ 0.2 ng/mL at week 25 in each of three arms.*

	• *PSA decline ≥ 50 and ≥ 80% at week 25*<br>• *PSA progression rate at week 25 (PCWG3 criteria)*<br><br>***Sensitivity analysis:*** *It was performed a sensitivity analysis for the primary endpoint. All randomized patients were included in this analysis. Patients with missing PSA data at week 25 were labeled as failures.*<br>• *Primary endpoint: proportion of patients who achieve an undetectable PSA level, defined as ≤ 0.2 ng/mL at week 25 in each of three arms.*<br><br>*To evaluate the radiographic progression rate at week 25, only patients who had the tumor assessment images evaluable at week 25 were considered.*<br>***Safety Population:*** *The safety population includes all subjects who received at least 1 dose of study drug.*<br>***Patient-reported Outcomes Population [PRO]****: The PRO population includes randomized subjects who have completed the baseline assessment and at least one postbaseline assessment of BPI-SF and FACT-P questionnaires."*<br><br>**Efficacy analyses**<br>Demographics and baseline disease characteristics will be analyzed using descriptive statistics. Subject's age, height, weight and other quantitative baseline characteristics will be summarized by number of patients with available information in each characteristic, mean, standard deviation, median, minimum, maximum and quartiles, when appropriate. Categorical variables (age group, gender, race, histology and others) will be summarized using frequency tabulations (count and percent).<br><br>**Analysis of primary endpoint**<br>The proportion of patiens in each arm who achieves a PSA level ≤ 0.2 ng/mL at week 25 will be evaluated as the number of patients with an undetectable PSA level at week 25 divided by the number of patients randomized for each group, according to ECOG status and disease volume.<br>The analyses of the primary endpoint will be evaluated per modified ITT population and in a sensitivity analysis population.

Analysis of secondary endpoints

Secondary objectives related to hormonal levels, ~~bone density~~, safety profile and quality among the three arms will be summarized descriptively.

The proportion of patients with a PSA response at week 25, calculated as the number of patients with PSA response (≥ 50% and ≥ 80% PSA decline from baseline) at week 25 divided by the number of randomized patients in each group, will be presented as the percentage of patients responding with the corresponding 95% CI based in the exact binomial distribution. PSA progression rate will be evaluated according to PCWG3 criteria at week 25 and 53 from time to randomization. And the maximum PSA declines and overall PSA change from baseline up to week 25 and up to week 53 will be summarized descriptively among the three arms.

~~Radiographic progression-free survival (rPFS) based on Prostate Cancer Working Group 3 and RECIST 1.1 will be assessed from the randomization to week 25.~~

New protocol amendment (refers to strikethrough text): *Radiographic progression rate at week 25 - according to RECIST 1.1 criteria. Only patients who had the tumor assessment images evaluable at week 25 and with overall response able to assess were evaluated.*

Hormonal levels testosterone during treatment ~~and bone density~~ will be described ~~and compared~~ between arms and correlated with primary endpoint.

Time-to-event endpoint will be estimated by Kaplan-meier method ~~and compared~~ by stratified log-rank test or Cox regression method. Dichotomic data will be analysed using Fischer's exact test or Chi-squared test. If necessary, other methods for categorical data may also be applied as appropriate.

Safety analyses

Subjects who receive at least 1 dose of study drug will be analyzed for safety. The safety parameters to be evaluated are the incidence, intensity, and type of adverse events, clinically significant changes in the subject's physical examination findings, vital signs measurements, and clinical laboratory results. Exposure to study drug and reasons for discontinuation of study treatment will be tabulated.

Laboratory abnormalities

Clinical laboratory test results will be collected from Screening and through 28 days after last dose of study drug. Laboratory data will

	be summarized by type of laboratory test. Parameters with predefined NCI-CTCAE toxicity grades will be summarized. Change from baseline to the worst AE grade experienced by the subject during the study will be provided as shift tables.<br><br>**Other safety data**<br>Other safety data will be summarized descriptively.<br><br>**Vital Signs**<br>Descriptive statistics of blood pressure (systolic and diastolic) values and changes from baseline will be summarized at each scheduled time point. Descriptive statistics of other vital signs at baseline will also be summarized. The number and percentage of subjects with values beyond clinically important limits will be summarized.<br><br>**Resource utilization**<br>Medical resource utilization will be descriptively summarized by treatment group. Results of these analyses will be reported separately and will not be a part of the clinical study report.<br><br>**Treatment compliance**<br>Each arm will be summarized according to the planned dose. For those patients unable to tolerate the protocol-specified dosing schedule, dose adjustments will be analysed, if necessary. Also, the number of patients analysed in each arm will be checked, once patients withdraw prematurely are possible (the reason for withdraw will be summarized too).
**Translational research**	This study will collect biological material from patients and create a biorepository for translational research projects. The biorepository will comply with the current regulations in Brazil. Detailed information about biomaterial collection, handling and shipment will be sent to participating sites in a separate document called “Manual of Biological Material Collection”.

1 Background

Patients with advanced prostate cancer are generally treated with surgical or chemical castration. Despite high response rates with this strategy, testosterone suppression is associated with libido loss, sexual dysfunction, hot flushes, osteoporosis, muscle weakness and weight gain.[1] Moreover, patients with metastatic prostate cancer are living longer as a result of several new life prolonging treatments and therefore the long-term side-effects of castration are becoming more common and an increasingly challenging aspect of patient management.[2] Additionally, a high proportion of patients with metastatic disease are asymptomatic, most notably when androgen deprivation therapy is initiated for rising prostate specific antigen (PSA) after radical therapy. Therefore, there is a need to investigate other hormonal strategies that can be as effective as testoterone supression and at the same time not associated with side-effects associated with castration.[3,4]

Abiraterone acetate, a prodrug of abiraterone, is a selective inhibitor of androgen biosynthesis that potently blocks cytochrome P450 c17 (CYP17), a critical enzyme in testosterone synthesis, thereby blocking androgen synthesis by the adrenal glands and testes and within the prostate tumor.[5] In phase 1–2 trials including patients with castration prostate cancer, treatment with abiraterone acetate, either as a single agent or in combination with low-dose glucocorticoids such as prednisone, resulted in significant antitumor activity among both patients with progressing castration-resistant prostate cancer who had not received chemotherapy and those who had received chemotherapy.[6-12] Abiraterone acetate was evaluated in patients with castration resistant prostate cancer according to two randomized clinical trials post and pre-docetaxel. In the first Phase III study a total of 1195 patients who had previously received docetaxel were randomized to receive 5 mg of prednisone twice daily with either 1000 mg of abiraterone acetate (797 patients) or placebo (398 patients). After a median follow-up of 12.8 months, overall survival was longer in the abiraterone acetate–prednisone group than in the placebo–prednisone group (14.8 months vs. 10.9 months; hazard ratio, 0.65; 95% confidence interval, 0.54 to 0.77; P<0.001). All secondary end points, including time to PSA progression (10.2 vs. 6.6 months; P<0.001), progression-free survival (5.6 months vs. 3.6 months; P<0.001), and PSA response rate (29% vs. 6%, P<0.001), favored the treatment group. The tolerance was excellent and side-effects were in general mild and transitory.[13,14] In the second Phase III study a total of 1088 patients who had not previously received chemotherapy were randomized to receive abiraterone acetate (1000 mg) plus prednisone (5 mg twice daily) or placebo plus prednisone. The median radiographic progression-free survival was 16.5 months with abiraterone-prednisone and 8.3 months with prednisone alone (hazard ratio for abiraterone-prednisone vs. prednisone alone, 0.53; 95% confidence interval [CI], 0.45 to 0.62; P<0.001). Abiraterone-prednisone showed superiority over prednisone alone with respect to time to initiation of cytotoxic chemotherapy, opiate use for cancer-related pain, prostate-specific antigen progression, and decline in performance status. Grade 3 or 4 mineralocorticoid-related adverse events and abnormalities on liver-function testing were more common with abiraterone-prednisone.[15]

Apalutamide is a second-generation antiandrogen that emerged from a structure/activity relationship–guided medicinal chemistry program to design more potent antiandrogens with no significant agonistic activity in the setting of AR overexpression.[16] The drug binds to the ligand-binding domain of AR with five-fold greater affinity than bicalutamide, and unlike bicalutamide, it does not induce robust AR nuclear translocation or DNA binding. In preclinical model systems, ARN-509 induced partial or complete regression in both castration-sensitive and -resistant human prostate cancer xenograft models and showed maximal antitumor efficacy in these models at a three-fold lower dose and approximately nine-fold lower plasma level than enzalutamide, suggestive of a higher therapeutic index.[17] A Phase I study including 30 patients with castration resistant disease reported the dose of 240mg qd suitable for Phase II trials. Prostate-specific antigen declines at 12 weeks (≥ 50% reduction from baseline) were observed in 46.7% of patients.[16] A Phase II trial including 21 patients with castration resistant prostate cancer who had failed prior abiraterone treatment has shown a response rate of 24%. These results indicate that there is non-cross resistance between ARN-509 and abiraterone.[18]

Co-targeting the androgen receptor and paracrine androgen biosynthesis in castration resistant prostate cancer may be more effective than either alone. A Phase II study evaluated the activity of abiraterone and enzalutamide, second-generation antiandrogen, at the conventional doses in 60 patients and reported a PSA decline ≥ 50% and ≥ 90% in 76 and 45% of patients, respectively. Grade 3 adverse events included: ALT rise (5), hypertension (5), ALP rise (4), arthralgia (3), bone pain (2). No Grade 4 adverse events were reported.[19]

Like APALUTAMIDE, enzalutamide works through competitive AR inhibition that is purely antagonistic. A phase II trial assessed enzalutamide monotherapy in men either presenting with metastatic prostate cancer or relapsing after radical treatment to the prostate and who had not received previous systemic treatment. From 67 men enrolled into the study, 62 patients (92.5%, 95% CI 86.2-98.8) had a decline in PSA of 80% or greater at week 25. This finding provided a rationale for further investigation of clinical response and outcomes with androgen inhibitor or other drugs such as abiraterone in non-castrate men with prostate cancer.[20]

Except for a Phase I trial evaluating the safety profile and the maximum tolerated dose with abiraterone,[2116] none trial have evaluated the activity, safety profile, and hormone levels of abiraterone, APALUTAMIDE 240mg PO qd, and the combination of both agents in metastatic prostate cancer with non-castrate testosterone levels. In a phase 1b trial with heavily treated CRPC patients, no new safety signals were found in 29 patients receiving abiraterone plus APALUTAMIDE.[21]

More recently, two randomized trials have shown a survival benefit of adding docetaxel to androgen suppression in hormone naïve prostate cancer. In CHAARTED trial, a total of 790 patients with metastatic, hormone-sensitive prostate cancer were randomized to receive either ADT plus docetaxel (at a dose of 75 mg per square meter of body-surface area every 3 weeks for six cycles) plus castration or castration alone. After a median follow-up of 28.9 months, the median overall survival was 13.6 months longer with

castration plus docetaxel (combination therapy) than with castration alone (57.6 months vs. 44.0 months; hazard ratio for death in the combination group, 0.61; 95% confidence interval [CI], 0.47 to 0.80; P<0.001). The benefit in survival was more evident in patients with high volume disease. The median time to biochemical, symptomatic, or radiographic progression was 20.2 months in the combination group, as compared with 11.7 months in the castration-alone group (hazard ratio, 0.61; 95% CI, 0.51 to 0.72; P<0.001). The rate of a prostate-specific antigen level of less than 0.2 ng per milliliter at 12 months was 27.7% in the combination group versus 16.8% in the ADT-alone group (P<0.001). In the combination group, the rate of grade 3 or 4 febrile neutropenia was 6.2%, the rate of grade 3 or 4 infection with neutropenia was 2.3%, and the rate of grade 3 sensory neuropathy and of grade 3 motor neuropathy was 0.5%.[22] In STAMPEDE trial 2,962 patients with high-risk locally advanced or metastatic hormone-sensitive prostate cancer were randomized in a 2:1:1:1 ratio to receive either ADT alone, ADT plus docetaxel (at a dose of 75 mg per square meter of body-surface area every 3 weeks for six cycles), ADT plus zoledronic acid, or ADT plus docetaxel plus zoledronic acid. After a median follow-up of 42 months, there were 405 deaths on the control arm. The hazard ratio was 0.76 (95% CI 0.63, 0.91; p = 0.003) for docetaxel plus castration. Median survival was increased by 10 months from 67 months on castration alone arm to 77 months on docetaxel plus castration. Grade 3-5 toxicity was reported for 31% in the castration alone arm, 50% for docetaxel plus castration arm, 32% for zoledronic acid plus castration arm, and 52% for docetaxel plus castration plus zoledronic acid arm.[23] As toxicity in both trials were higher in the chemotherapy/hormonal arms despite the survival benefit and not uncommonly patients due to age and/or comorbidities are no suitable candidates for chemotherapy, there is an urgent need to study and validate regimens such as new hormonal agentes that may add benefit to castration with an acceptable safety profile.

PSA kinetics seems to be a surrogate marker for outcome in patients with advanced prostate cancer. As an example, a retrospective study including 332 eligible patients with histologically confirmed and hormonally-naïve prostate cancer has shown that reaching the undetectable PSA level (<0.2ng) was the independent risk factors for predicting good prognosis (sensitivity 65.7%, specificity 80.6%).[24] A sub-analysis of the SWOG 9346 (INT-0162) phase III trial showed that PSA value of <= 4 and <= 0.2 after androgen deprivation is a strong independent predictor of survival in newly metastatic prostate cancer.[25]

2 Study rationale/purpose

Based on the current guidelines, ADT alone or combined with is antiandrogens are considered the appropriate active therapy for the patient population planned for this study. Recent data showed that chemotherapy also benefit patients in this setting. Even though, there is a clear unmet medical need for alternative treatment option in metastatic hormone sensitive prostate cancer (mHSPC). Treatments that can delay disease progression, and are associated with less comorbidities would be of significant clinical benefit in this patient population. Our study is designed to assess the efficacy and safety

of abiraterone plus APALUTAMIDE (a second-generation antiandrogen) or APALUTAMIDE alone without castration side effects and the other arm a combination of ADT and abiraterone; this last arm is based on the results of LATITUDE trial, that demonstrated a survival benefit of adding abiraterone to castration in this setting of patients. Abiraterone had already showed clinical benefit in CRPC patients without prior chemotherapy.

3 Objectives

We aim to assess the activity, safety and patients reported outcome of ADT plus abiraterone, abiraterone plus APALUTAMIDE (a second-generation antiandrogen) or APALUTAMIDE alone in hormone naïve locally advanced or metastatic prostate cancer which ADT was indicated.

3.1 Primary objective

The primary objective is to evalute the proportion of patients who achieves an undetectable PSA level, defined as ≤ 0.2 ng/mL at week 25 in each of three arms.

3.2 Secondary objectives

- To determine ~~and compare~~ PSA progression rate at week 25 (PCWG3 criteria);

- To determine ~~and compare~~ PSA response of 50 and 80% at week 25;

- To determine maximum PSA declines and overall PSA change from baseline up to week 25 and up to week 53;

- To determine the radiographic progression rate at week 25 among the three arms;

- To determine ~~and compare~~ hormonal levels during treatment;

- To determine ~~and compare~~ the safety profile;

- To determine and compare the time to pain progression assessed by BPI-SF and opioid use;

- To determine and compare the quality of life assessed by FACT-P;

- To determinetime to prostate cancer castration resistance;

- Metastasis free survival (on non-metastatic patients at inclusion).
- 2-year overall survival rate

- Time to next treatment.

4 Study design

This is a phase II, open label, randomized trial evaluating the efficacy of abiraterone acetate 1000mg PO QD plus prednisone 5mg PO BID and Androgen Deprivation Therapy (ADT) versus APALUTAMIDE 240mg PO versus the combination of abiraterone acetate 1000mg PO QD plus prednisone 5mg PO BID and APALUTAMIDE(without ADT), both at the standard doses, in patients with advanced or metastatic prostate cancer with non-castrate testosterone levels. Efficacy will be measured primarily by assessing percentage of patients who achieves an undetectable PSA level defined as ≤ 0.2 ng/mL at week 25. A second efficacy analysis will be done at week 53. The total study period is 2 years including patient treatment and outcome data collection. Patients will be treated until objective or clinical disease progression or occurrence of an unacceptable toxicity. Patients are allowed to continue study treatment beyond the 25-week assessment (extension phase) at the discretion of the investigator.

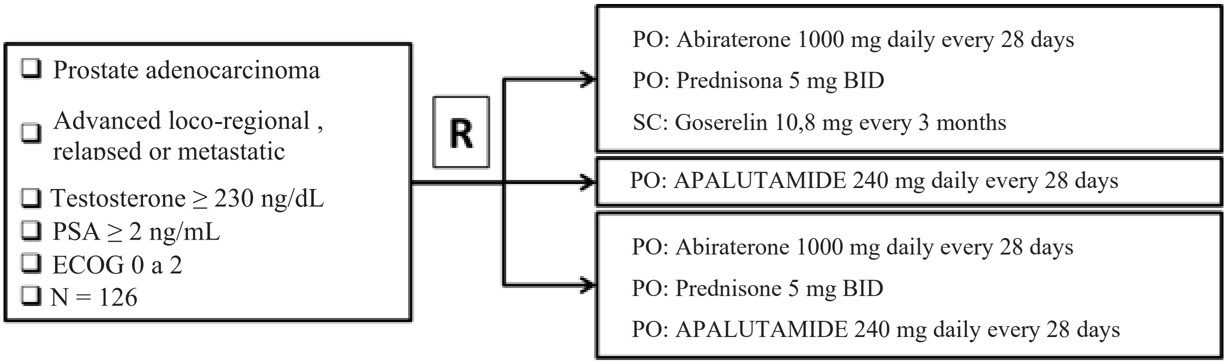

5 Population

5.1 Inclusion criteria

Each potential subject must fulfill all of the following criteria to be enrolled in the study.

1. Histologically confirmed prostate adenocarcinoma;
2. Patients with indication to start treatment with ADT in one of the following settings:

a. Biochemical relapse after definitive treatment (surgery and/or radiotherapy): PSA ≥ 4 ng/ml and doubling time less than 10 months, or PSA ≥ 20 ng/ml;

b. Newly diagnosed Prostate Cancer: locally advanced – T_{any} N+ M0 (not candidate to definitive treatment with surgery or radiotherapy) or metastatic – T_{any} N_{any} M+ and PSA ≥ 2 ng/mL;

3. Patient is asymptomatic to moderately symptomatic regarding bone symptoms, i.e., no need for palliative radiation or radionuclide therapy;
4. Complete staging process (performed as per routine), meaning, thorax, abdomen and pelvis TC and bone scan, performed before consent and that do not exceed 8 weeks from the date of randomization;
5. Non-castration level of testosterone ≥ 230ng/dL (> 8 nmol/L);
6. ECOG performance status of 0 to 2;
7. Adequate hematologic, hepatic and renal function:
    a. hemoglobin > 10 g/dL, neutrophils > 1.5×10^9 / L, platelets> 100×10^9 / L;
    b. total bilirubin < 1.5x upper limit of normal (ULN); alanine (ALT) and aspartate (AST) aminotransferase < 2.5 x ULN;
    c. serum creatitine < 1.5x ULN; potassium > 3.5 mM;
8. Written informed consent obtained prior to any study procedure;
9. Men age 18 years and older;
10. Agrees to use a condom and another effective method of birth control if he is having sex with a woman of childbearing potential or agrees to use a condom if he is having sex with a woman who is pregnant.

5.2 Exclusion criteria

Any potential subject who meets any of the following criteria will be excluded from participating in the study.

1. Prostate adenocarcinoma with neuroendocrine differentiation or small cell histology;
2. Use of hormonal therapy or chemotherapy prior to randomization. Exception is courses of hormone therapy for localised disease must have been completed at least 12 months previously. It can have been given as adjuvant or neoadjuvant

therapy.

3. Prior radiation therapy for a primary tumour within the 3 months before enrollment or for the treatment of metastases;
4. Known or suspected brain or skull metastases or leptomeningeal metastatic disease;
5. Any concurrent severe and/or uncontrolled medical conditions which could compromise participation in the study;
6. Administration of an investigational therapeutic or invasive surgical procedure within 28 days of Cycle 1 Day 1 or currently enrolled in an investigational study;
7. Active or symptomatic viral hepatitis or chronic liver disease; ascites or bleeding disorders secondary to hepatic dysfunction;
8. Current or prior treatment with anti-epileptic medications for the treatment of seizures;
9. Impaired cardiac function, including any of the following:
    a. Uncontrolled hypertension (systolic blood pressure ≥160 mmHg or diastolic BP ≥95 mmHg);
    b. Clinically significant heart disease as evidenced by myocardial infarction, or arterial thrombotic events or history of cardiac failure in the past 6 months, severe or unstable angina, or New York Heart Association (NYHA) Class II-IV heart disease;
    c. Existing atrial fibrillation with or without pharmacotherapy. Other cardiac arrhythmia requiring pharmacotherapy;
10. History of seizure or condition that may predispose to seizure (including, but not limited to prior stroke, transient ischemic attack or loss of consciousness ≤1 year prior to randomization; brain arteriovenous malformation; or intracranial masses such as schwannomas and meningiomas that are causing edema or mass effect);
11. Specific underlying conditions for oral agents. For example: impairment of gastrointestinal (GI) function or GI disease that may significantly alter the absorption of abiraterone or APALUTAMIDE (e.g., ulcerative diseases, uncontrolled nausea, vomiting, diarrhea, malabsorption syndrome, or small bowel resection)

12. General excluded medications (e.g., relevant to cytochrome P450 interactions)
    a. Use of prescription drugs within 14 days prior to dosing or over-the-counter (OTC) medication within 7 days prior to dosing;
    b. Consumption of grapefruit product or St John's wort within 7 days prior to dosing;
    c. G-CSF, GM-CSF, erythropoietin, etc;
    d. Coumadin;
    e. Drugs which may cause QT prolongation;
    f. Known sensitivity to drugs or metabolites from similar classes;
    g. Known or suspected contraindications or hypersensitivity to APALUTAMIDE, bicalutamide or GnRH agonists or any of the components of the formulations;
13. Any condition or situation which, in the opinion of the investigator, would put the subject at risk, may confound study results, or interfere with the subject's participation in this study;
14. Surgical castration prior to study entry;
15. Had a prior malignancy. Adequately treated basal cell or squamous cell carcinoma of skin or superficial bladder cancer that has not spread behind the connective tissue layer (i.e., pTis, pTa, and pT1) is allowed, as well as any other cancer for which treatment has been completed 5 years before randomization and from which the subject has been disease-free.

5.3 Prohibitions and Restrictions

1. If the subject is engaged in sexual activity with a partner, a condom is required.

If the subject is engaged in sexual activity with a woman of childbearing potential, a condom is required along with another effective contraceptive method consistent with local regulations regarding the use of birth control methods for subjects participating in clinical studies and their partners, including:

- established use of oral, injected or implanted hormonal methods of contraception;
- placement of an intrauterine device (IUD) or intrauterine (IUS) system;
- barrier methods: condom with spermicidal foam/gel/film/cream/suppository or

occlusive cap (diaphragm or cervical/vault caps) with spermicidal foam/gel/film/cream/suppository;

- vasectomy.

2. Two highly effective forms of contraception are required during the Treatment Phase and for 3 months after the last dose of study drug;
3. Orchiectomy is prohibited to enter the study and during the Treatment Phase.

6 Study drug information

6.1 Investigational and control drugs

This is a three-arm study consisted of:

- **Arm 1**: Abiraterone acetate + Prednisone + ADT (Goserelin).
  - Abiraterone administered at a single 1000 mg daily oral dose (4 x 250-mg tablets)
  - Prednisone administered at a 5 mg twice daily oral dose
  - Goserelin administered as subcutaneous injections of 10.8mg every 3 months

- **Arm 2:** APALUTAMIDE monotherapy
  - APALUTAMIDE administered at a single 240 mg daily oral dose (4 x 60 mg tablets)

- **Arm 3:** Abiraterone acetate + Prednisone + APALUTAMIDE
  - Abiraterone administered at a single 1000 mg daily oral dose (4 x 250 mg tablets)
  - Prednisone administered at a 5 mg twice daily oral dose
  - APALUTAMIDE administered at a single 240 mg daily oral dose (4 x 60 mg tablets)

Per protocol study treatment is planned until week-25. Patients will be treated until objective or clinical disease progression or occurrence of an unacceptable toxicity. Patients are allowed to continue study treatment beyond the week 25 (extension phase) at the discretion of the investigator.

Patients will be discontinued from the planned study treatment due to progression (radiographic per RECIST 1.1 and/or symptomatic +/- biochemical according to PCWG3 criteria), adverse event or patient withdrawal.

6.2 Treatment Allocation

Subjects will be randomly assigned to the each active arm in a 1:1:1 ratio after the investigator has verified that all eligibility criteria have been met. The randomization will be balanced by using randomly permuted blocks. Randomization will take place across all study sites using a centralized Interactive Web Response System (IWRS). Subjects will be stratified by performance status (ECOG 0-1 vs 2) and metastatic disease (yes vs. no).

At study inclusion (right after informed consent has been obtained), the eCRFwill assign a unique subject identification number to each subject. The subject’s identification number will be used on all study-related documents.

Subject identification numbers will not be reused. Subjects withdrawn from the study will not be replaced. All subjects must commence treatment within 72 hours (3 calendar days) of randomization date, in which an official statement is sent to the research site from LACOG (via e-mail).

6.3 APALUTAMIDE

- Physical Description of Study Drug:

The APALUTAMIDE tablet supplied for this study contains 60-mg of APALUTAMIDE. It will be manufactured and provided under the responsibility of the sponsor. Refer to the Investigator's Brochure for a list of excipients.

- Packaging:

APALUTAMIDE 60-mg tablets will packaged in 120-count, 160 cc high-density polyethylene (HDPE) bottles with child-resistant closures.

- Labeling:

Study drug labels will contain information to meet the applicable regulatory requirements.

- Preparation, Handling and Storage:

The study drug must be stored in a secure area and administered only to subjects entered into the clinical study in accordance with the conditions specified in this protocol. Apalutamide must be stored at temperatures from 15ºC – 30ºC.

Refer to the pharmacy manual for additional guidance on study drug preparation, handling and storage.

6.4 Abiraterone acetate

- Physical Description of Study Drug:

Abiraterone acetate 250 mg tablets are oval, white to off-white and contain abiraterone acetate and compendial (USP/NF/EP) grade lactose monohydrate, microcrystalline cellulose, croscarmellose sodium, povidone, sodium lauryl sulfate, magnesium stearate, colloidal silicon dioxide, and purified water, in descending order of concentration (the water is removed during tabletting). Prednisone capsules will also be provided.

- Packaging:

The study drugs will be packaged and supplied by the sponsor. Abiraterone acetate is packaged in high-density polyethylene bottles with child-resistant closures. There are 120 tablets per bottle. Prednisone is packaged in blister packs. There are 30 capsules per pack.

- Labeling:

Study drug labels will contain information to meet the applicable regulatory requirements.

- Preparation, Handling, and Storage:

The study drugs must be stored in a secure area and administered only to subjects entered into the clinical study in accordance with the conditions specified in this protocol. Study drugs should be stored at room temperature. Additional information is provided in the abiraterone acetate Investigator's Brochure. Subjects should be advised to keep all medications out of reach and sight of children.

Abiraterone acetate is contraindicated in women who are or may potentially be pregnant. There are no human data on the use of abiraterone acetate in pregnancy. Maternal use of CYP17 inhibitor is expected to produce changes in hormonal levels that may affect the development of the fetus. Women who are pregnant or may be pregnant should not handle abiraterone acetate without protection, eg gloves.

In an oral developmental toxicity study in the rat, abiraterone acetate affected pregnancy.

It is not known whether abiraterone or its metabolites are present in semen. A condom is required if the subject is engaged in sexual activity with a pregnant woman. If the subject is engaged in sex with a woman of childbearing potential, a condom is required along with another effective contraceptive method.

6.5 ADT

All subjects will receive and remain on a stable regimen of ADT (GnRHa) as follows: goserelin 10.8 mg every 3 months. Dosing (dose and frequency of administration) will be consistent with the prescribing information.

6.6 Drug Accountability

The investigator is responsible for ensuring that all study drug received at the site is inventoried and accounted for throughout the study. The dispensing of study drug to the subject, and the return of study drug from the subject, must be documented on the drug accountability form. Subjects must be instructed to return all original containers, whether empty or containing study drug. These requirements also apply to prednisone capsules supplied by the sponsor.

All study drugs must be handled in strict accordance with the protocol and the container label, and must be stored at the study site in a limited-access area or in a locked cabinet under appropriate environmental conditions. Unused study drug, and study drug returned by the subject, must be available for verification by the sponsor's site monitor during on-site monitoring visits. The return to the sponsor of unused study drug, or used returned study drug for destruction, will be documented on the drug return form. When the site is an authorized destruction unit and study drug supplies are destroyed on site, this must also be documented on the drug return form.

All study drugs should be dispensed under the supervision of the investigator or a qualified member of the investigational staff, or by a hospital/clinic pharmacist. Study drug will be supplied only to subjects participating in the study. Returned study drug must not be dispensed again, even to the same subject. Whenever a subject brings his or her study drug to the site for pill count, this is not seen as a return of supplies. Study drug may not be relabeled or reassigned for use by other subjects. The investigator agrees neither to dispense the study drug from, nor store it at, any site other than the study sites agreed upon with the sponsor.

7 Dosage and administration

7.1 Planned dose

a) **Abiraterone acetate**

Abiraterone acetate 1,000 mg (four 250 mg tablets) should be taken orally once daily, in combination with oral dose prednisone 5mg twice daily continuously. For the purpose of scheduling the study assessments and treatment compliance a treatment cycle is defined as 28 days. Abiraterone acetate must be taken on an empty stomach. No food should be consumed for at least 2 hours before the dose of abiraterone acetate is taken and for at least 1 hour after the dose of abiraterone acetate is taken. Tablets should be swallowed whole with water. Prednisone dose need not be taken at the same time as the abiraterone acetate dose. If an abiraterone acetate dose is missed (meaning, 12 hours have passed of

the usual time of intake), it should be omitted and will not be made up. Similarly if the prednisone dose is missed, it should be omitted and not made up.

b) **APALUTAMIDE**

APALUTAMIDE 240-mg orally once daily (4 x 60-mg tablets) will be administered on a continual basis. For the purpose of scheduling the study assessments and treatment compliance a treatment cycle is defined as 28 days. APALUTAMIDE can be taken with or without food. If a dose of APALUTAMIDE is missed (meaning, 12 hours have passed of the usual time of intake) it should be omitted and will not be made up or taken with the next dose the following day. Please refer to the pharmacy manual for further details. Study drug administration must be captured in the source documents and the eCRF.

c) **ADT**

Arm 1 subjects will receive and remain on a stable regimen of ADT (LHRH agonists). Dosing of goserelin (dose and frequency of administration) will be consistent with the prescribing information and should only be adjusted if clinically indicated to achieve and maintain subcastrate concentrations of testosterone (50 ng/dL or 1.7 nM).

7.2 Dosing modifications and Management of Toxicity

For patients who are unable to tolerate the protocol-specified dosing schedule, dose adjustments are permitted in order to keep the patient on study drug. The following guidelines should be followed:

These changes must be recorded on the field “Study Medication” of the eCRF.

7.2.1 Abiraterone acetate

In clinical studies in subjects with mCRPC, the most common adverse events related to abiraterone acetate have included fatigue most likely attributable to the underlying disease; and hypertension, hypokalemia, fluid retention/edema, and due to mineralocorticoid excess caused by compensatory ACTH drive. In this study, low-dose prednisone is expected to mitigate these effects through abrogation of the ACTH drive.

Following prolonged therapy with corticosteroids, subjects may develop Cushings syndrome characterized by central adiposity, thin skin, easy bruising, and proximal myopathy. Withdrawal of the corticosteroid may result in symptoms that include fever, myalgia, fatigue, arthralgia, and malaise. This may occur even without evidence of adrenal insufficiency.

In the event where dose-reduction is used for adverse event management, 2 dose reductions are allowed. At each dose reduction, 1 tablet of abiraterone acetate will be removed, eg, 4→3 tablets, and 3→2 tablets. Any return to protocol dose level (4 tablets) after dose reduction must follow documentation of adverse event resolution and a discussion with the sponsor.

a. **Guidelines for Abnormal Liver Function Test**

For subjects who develop liver function test abnormalities (ALT and/or AST greater than 5 X ULN but not exceeding 20 X ULN or total bilirubin greater than 3 X ULN but not

exceeding 10X ULN), study drug must be interrupted. If increases in ALT and/or AST of Grade 2 (increase in ALT and/or AST to >2.5 to 5X ULN) or Grade 3 (increase in ALT and/or AST to > 5X ULN) occur, the frequency of liver function test monitoring must be increased to at least once a week. Treatment may be restarted at a reduced dose of 750 mg (3 tablets) once daily following return of liver function tests to the subject's baseline or to AST and ALT less than or equal to 2.5 X ULN and total bilirubin less than or equal to 1.5 X ULN.

For subjects with ALT and/or AST greater than 20 X ULN or total bilirubin greater than 10 X ULN, study drug must be interrupted. The decision to restart treatment at a reduced dose will be made in consultation with the sponsor medical monitor on an individual basis.

For subjects who resume treatment, serum transaminases and bilirubin should be monitored at a minimum of every 2 weeks for 3 months and monthly thereafter.

If liver function test abnormality recurs at the dose of 750 mg (3 tablets) once daily, re-treatment may be restarted at a reduced dose of 500 mg (2 tablets) once daily following return of liver function tests to the subject's baseline or to AST and ALT less than or equal to 2.5X ULN and total bilirubin less than or equal to 1.5X ULN.

If liver function test abnormality recurs at the reduced dose of 500 mg (2 tablets) once daily, study drug must be discontinued.

b. **Guidelines for Hypertension, Hypokalemia and Fluid Retention/Edema Due to Mineralocorticoid Excess**

The study drug should be used with caution in subjects with a history of cardiovascular disease. The study drug may cause hypertension, hypokalemia, and fluid retention as a consequence of increased mineralocorticoid levels resulting from CYP17 inhibition. Caution should be exercised when treating subjects whose underlying medical conditions might be compromised by increases in blood pressure, hypokalemia, or fluid retention.

In patients with pre-existing hypokalemia or those who develop hypokalemia on study treatment, consider maintaining the subject’s potassium level at 4.0 mM or higher according to local clinical practice.

For subjects who develop drug-related Grade 3 or higher toxicities, including hypertension, hypokalemia, edema, and other non-mineralocorticoid toxicities, treatment must be withheld and appropriate medical management should be instituted. Treatment with abiraterone acetate must not be reinitiated until symptoms of the toxicity have resolved to Grade 1 or baseline.

7.2.2 APALUTAMIDE

Dose Modifications for Toxicity Attributed to APALUTAMIDE:

Toxicity	Number of ARN509 tablets

Grade 1 or 2	No change or hold until return to baseline
≥Grade 3	Hold until Grade 1 or baseline, resume at full dose
Recurrence ≥Grade 3	Hold until Grade 1 or baseline; 2 dose reductions are allowed for recurrent treatment-related toxicity (180 mg [3 tablets]) and 120 mg [2 tablets]). Discontinue if toxicity persists after 2 dose reductions.
First occurrence of seizure of any grade or Grade 4 neurotoxicity	Discontinue

7.3 Treatment Compliance

Accurate records of all drug shipments as well as tablets dispensed and returned will be maintained. This inventory must be available for inspection by designated sponsor or regulatory authority representatives at any time. Drug supplies are to be used only in accordance with this protocol and under the supervision of the investigator. Study drugs administration and dosing compliance will be assessed at each study visit, starting with Cycle 2. A count of all study drugs provided by the sponsor will be conducted during the Treatment Phase.
If dosing compliance is not 100% in the absence of toxicity, then subjects should be re-instructed regarding proper dosing procedures and may continue with the study. Subsequent dosing compliance procedure will be conducted at each study visit. If the number of study drugs doses taken by the subject is ≤75% in the absence of toxicity or disease progression, then subjects should be re-instructed regarding proper dosing procedures. Subjects who have study drugs dosing compliance of ≤75% for 2 consecutive cycles should be discontinued from the study treatment.
In the occurrence of eventual delays, interruptions, lapses or discontinuations, the following cycles must be maintained in the planned dates according to the beginning of study medication intake and must not be adjusted.

The study site must maintain accurate records demonstrating dates and amount of study drug received, to whom dispensed (subject by subject accounting), and accounts of any study drug accidentally or deliberately destroyed. At the end of the study, reconciliation must be made between the amount of study drug supplied, dispensed, and subsequently destroyed or returned to sponsor or its representative.

7.4 Concomitant Treatment

7.4.1 Prestudy and Concomitant Therapy

All pre-study therapies administered up to 28 days before first dose of study drug must be recorded at screening. All concomitant therapies must be recorded on the subject's eCRF throughout the study beginning when the first dose of study drug is administered.

Concurrent enrollment in another investigational drug or device study during this study is prohibited. The sponsor must be notified in advance (or as soon as possible thereafter) of any instances in which prohibited therapies are administered. All therapies (prescriptions or over-the-counter medications, including vitamins and herbal supplements; non-pharmacologic therapies such as electrical stimulation, acupuncture, special diets, exercise regimens) different from study drug must be recorded in the concomitant therapy section of the eCRF.

7.4.2 Permitted Supportive Care and Interventions During Study

- Use of opioid analgesics as needed for cancer-related pain
- Bisphosphonates and denosumab for management of bone-related metastasis according to their market authorized approved label
- Surgical interventions and procedures such as transurethral resection of the prostate (TURP) and placement of ureteral stents for the management of complications due to local progression
- Conventional multivitamins, selenium and soy supplements
- Epelerenone can be used to manage mineralocorticoid-related toxicities
- Additional systemic glucocorticoid administration such as "stress dose" glucocorticoid is permitted when clinically indicated for a life threatening medical condition, and in such cases, the use of steroids will be documented as concomitant drug
- Transfusions and hematopoietic growth factors per institutional practice guidelines
- If the permissibility of a specific drug/treatment is in question, then please contact the study sponsor

7.4.3 Special Concomitant Therapy

The following concomitant therapies warrant special attention: Caution is advised when abiraterone acetate is administered with medicinal products activated by or metabolized by

CYP2D6, particularly with medicinal products that have a narrow therapeutic index. Dose reduction of medicinal products with a narrow therapeutic index that are metabolized by CYP2D6 should be considered. Examples of medicinal products metabolized by CYP2D6 include metoprolol, propranolol, desipramine, venlafaxine, haloperidol, risperidone, propafenone, flecainide, codeine, oxycodone and tramadol (the latter 3 products requiring CYP2D6 to form their active analgesic metabolites). Based on in vitro data, abiraterone acetate is an inhibitor of the hepatic drug-metabolizing enzyme, CYP2C8. Examples of medicinal products metabolized by CYP2C8 include paclitaxel and repaglinide. In a CYP2C8 drug-drug interaction trial in healthy subjects, the AUC of pioglitazone was increased by 46% and the AUCs for M III and M IV, the active metabolites of pioglitazone, each decreased by 10% when pioglitazone was given together with a single dose of 1000 mg abiraterone acetate. Although these results indicate that no clinically meaningful increases in exposure are expected when ZYTIGA is combined with drugs that are predominantly eliminated by CYP2C8, patients should be monitored for signs of toxicity related to a CYP2C8 substrate with a narrow therapeutic index (e.g. paclitaxel) if used concomitantly with ZYTIGA. Based on in vitro data, abiraterone is a substrate of CYP3A4. In a clinical pharmacokinetic interaction study of healthy subjects pretreated with a strong CYP3A4 inducer, rifampicin, 600 mg daily for 6 days followed by a single dose of abiraterone acetate 1,000 mg, the mean plasma AUC_∞ of abiraterone was decreased by 55%. Strong inducers of CYP3A4 (e.g., phenytoin, carbamazepine, rifampicin, rifabutin, rifapentine, phenobarbital, St John's wort [Hypericum perforatum]) during treatment are to be avoided, unless there is no therapeutic alternative. In a separate clinical pharmacokinetic interaction study of healthy subjects, co administration of ketoconazole, a strong inhibitor of CYP3A4, had no clinically meaningful effect on the pharmacokinetics of abiraterone. For the most current information regarding potential drug-drug interactions with abiraterone acetate, refer to the latest version of the reference safety information (Investigator's Brochure) for abiraterone acetate.

The investigator should instruct the patient to notify the study site about any new medications he takes after the start of the study drug. All medications (other than study drug) and significant non-drug therapies (including physical therapy and blood transfusions) administered after the patient starts treatment with study drug must be listed on the Concomitant Treatment session in the eCRF.

7.4.4 Prohibited Medications

- Investigational agents other than abiraterone acetate and APALUTAMIDE
- Other antineoplastic agents
- 5-α-reductase inhibitors
- Chemotherapy
- Immunotherapy
- Anti-androgens (e.g., bicalutamide, nilutamide, flutamide, cyproterone acetate) except in situations as outlined for management of tumor flare anticipated with the initiation of continuous LHRH agonist
- Systemic ketoconazole (or other azole drugs such as fluconazole or itraconazole)
- Diethylstilbestrol (DES) or similar
- Other preparations such as saw palmetto thought to have endocrine effects on prostate cancer
- Radiopharmaceuticals such as strontium (89Sr) or samarium (153Sm) or similar analogues such as radium-223 (223Ra)
- Spironolactone
- Digoxin, digitoxin, and other digitalis drugs
- Fludrocortisone acetate (Florinef)
- As a class effect, AR antagonists have been associated with seizures due to an off-target mechanism of action (gamma amino butyric acid chloride channel [GABAA] inhibition). Drugs known to lower the seizure threshold or cause seizures are prohibited and a representative list is included below:
    - Atypical antipsychotics (e.g. clozapine, olanzapine, risperidone, ziprasidone)
    - Bupropion
    - Lithium
    - Meperedine (pethidine)

• Phenothiazine antipsychotics (eg, chlorpromazine, mesoridazine, thioridazine)

• Tricyclic antidepressants (eg, amitriptyline, desipramine, doxepin, imipramine, maprotiline, mirtazapine

• Aminophylline/theophylline

7.5 Study drug discontinuation

If a subject's study drug must be discontinued before disease progression this will not result in automatic withdrawal of the subject from the study. All attempts should be made to capture radiographic progression even in subjects who have evidence of clinical progression. However, a subject's study treatment must be discontinued for:

• Radiological progression according to RECIST 1.1;

• Clinical progression defined as:

− Deterioration in ECOG PS grade to grade 3 or higher (related to prostate cancer progression)

− Need to initiate any of the following because of tumor progression (even in the absence of radiographic evidence of disease)

o Subsequent anti-cancer therapy for metastatic prostate cancer;

o Radiation therapy for metastatic prostate cancer lesion(s);

o Surgical interventions for complications due to metastatic prostate cancer progression. (Note: Surgical interventions for complications of local-regional progression are not considered clinical progression).

o Need for chronic opioid analgesics: For subjects entering the study without receiving opioids, chronic opioid use is defined as administration of opioid analgesics lasting for ≥3 weeks for oral or ≥7 days for non-oral formulations. For subjects entering the study already receiving opioids, chronic opioid use is defined as a ≥30% increase in total daily dose of the opioid analgesics lasting for ≥3 weeks for oral or ≥7 days for non-oral formulations. NOTE: Administration of as needed (eg, not fixed or scheduled dosage) use of opioid analgesics or extended opioid use for treatment other than the subject’s prostate cancer does not require discontinuation from study treatment (eg, codeine/acetaminophen combinations, hydrocodone/acetaminophen

combinations, hydrocodone/ibuprofen combinations, oxycodone/acetaminophen combinations, oxycodone/aspirin combinations, tramadol)

• More than 2 dose level reductions for Grade 3 or higher treatment-related AEs

• Seizure of any grade or Grade 4 neurotoxicity

• Dosing noncompliance

• Withdrawal of consent for continued treatment

• The investigator believes that for safety reasons (eg, AE) it is in the best interest of the subject to discontinue study treatment.

All attempts to obtain imaging studies at the time of treatment discontinuation should be made to assess for radiographic progression.

Study drug will be continued for subjects who have increasing PSA values in the absence of radiographic or clinical progression. Although serial PSA measurements will be performed in this study, progression (according to PCWG3 criteria) or change in PSA values should not be used as the lone indicator for disease progression or treatment discontinuation.

If a subject discontinues study drug, but does not withdraw consent, scheduled assessment should continue according to the Follow-up Phase in the Time and Events Schedule. All patients with more than 28 days without any of study drugs must be withdrawn from the study.

All patients must have evaluations for 28 days after the last dose of study treatment.

Patients lost to follow up should be recorded as such on the CRF.

Patients who discontinue study drug before completing the study should be scheduled for a visit as soon as possible, at which time all of the assessments listed for the final visit will be performed. At a minimum, all patients who discontinue study treatment, including those who refuse to return for a final visit, will be contacted for safety evaluations during the 28 days following the last dose of study drug.

Patients who discontinue study drug should be considered withdrawn from the study after the final visit assessments are performed or when it is clear that the patient will not return for these assessments.

7.6 Premature patient withdrawal

7.6.1 End of treatment

Patients **may** voluntarily withdraw from the study or be dropped from it at the discretion of the investigator at any time. Patients may be withdrawn from the study if any of the following occur: progressive disease (radiographic or clinical deterioration with or without PSA progression), toxicity not fully managed by study dug dose reduction, more than 28 days without any of the study drugs, patients refusing in continuing his participation or at the discretion of the investigator.

If such withdrawal occurs, or if the patient fails to return for visits, the investigator must determine the primary reason for a patient's premature withdrawal from the study and record this information on eCRF. Patients may be withdrawn from the study prematurely for one of the following reasons:

- Adverse event(s)
- Abnormal laboratory value(s)
- Abnormal test procedure result(s)
- Protocol violation
- Subject withdrew consent
- Lost to follow-up
- Administrative problems
- Death
- New cancer therapy
- Disease progression
- Treatment duration completed as per protocol

All cancer medications/therapies given to a patient ≤ 4 weeks after the last dose of study treatment must be recorded in the eCRF.

7.6.2 Study evaluation completion

As a general rule, if a patient discontinues study drug and later is prematurely withdrawn from the study, the reasons for study evaluation completion may include the following:

- Protocol violation
- Subject withdrew consent
- Lost to follow-up
- Administrative problems
- Death
- New cancer therapy
- Disease progression

For patients who are lost to follow-up, the investigator should show "due diligence" by documenting in the source documents steps taken to contact the patient, e.g., dates of telephone calls, registered letters, etc.

8 Visit schedule and assessments

All patients will be followed according to the flow chart depicted below.

Randomized study phase															Extension study phase	
Cycle number	Comments	Screening[5]	C1		C2		C3		C4	C5	C6	C7	PSA Confirmation	Safety visit	Patient chart data collection[4] Year 1	Patient chart data collection[4] Year 2
Week			**1**	**3**	**5**	**7**	**9**	**11**	**13**	**17**	**21**	**25**	**29**	30 days after the last dose of treatment[7]		
Day			1	1	1	1	1	1	1	1	1	1	1	1	1	1
Window		-28 to 0	- 3/+2 days days	± 3 days	-3/+2 days days	± 3 days	-3/+2 days days	± 3 days	-3/+2 days days	-3/+2 days days	-3/+2 days days	-3/+2 days days	± 7 days	± 3 days	± 7 days	± 7 days
**Screening, subjects evaluations and safety**																
Informed consent	To be performed before any study-specific procedures	X														
Medical history / clinical evaluation	Relevant medical history to be collected by investigator during screening.	X	X		X		X		X	X	X	X		X		
Vital signs	Blood pressure, heart rate, respiratory rate and body temperature at screening; afterwards only blood pressure.	X	X		X		X		X	X	X	X		X		
Physical examination	Height recorded only	X	X		X		X		X	X	X	X		X		

	at screening. Weight recorded at all applicable visits.															
Performance status (ECOG)		X	X		X		X		X	X	X	X		X		
12 lead ECG		X										X				
Echocardiogram or MUGA		X										X				
Concomitant medication assessment		X	X		X		X		X	X	X	X		X		
Study drug administration	To be given no later than 72 hours after randomization. *Goserelin 10.8mg to be administrated every 3 months.		X*		X		X		X*	X	X					
Study drug accountability			X		X		X		X	X	X	X				
Adverse events assessment		X	X	X[6]	X	X[6]	X	X[6]	X	X	X	X		X		
Survival Status	Biochemical and radiological progression and survival status														X	X
**Laboratory**																
Hematology	Hemoglobin, WBC, neutrophil count and platelet count.	X					X			X		X		X		
Biochemistry	LDH, potassium, creatinine, total bilirubin, AST,	X			X		X		X	X	X	X		X		

	ALT, albumin and fasting glucose.															
Hepatic evaluation	Total bilirubin, AST, ALT, albumin			X[6]		X[6]		X[6]								
PSA	To be collected within 7 days before randomization	X			X		X		X	X	X	X	X			
Testoterone levels	To be collected within 7 days before randomization	X			X		X		X	X	X	X	X			
**Efficacy**																
CT scans	Computed tomography of thorax, abdomen and pelvis. Response evaluation will be based on RECIST 1.1 criteria											X				
Bone scan														X		
Patient reported outcomes (PRO)	Adapted BPI-SF (question 3) and FACT-P		X		X		X		X	X	X	X				
**Biomaterial research[1]**																
FFPE			X													
Blood			X									X[2]			X[2]	X[2]

Legend:
1. TC scan assessments of chest, abdomen and pelvis and bone scan prior to study entry (local assessment performed as per routine) must be within a 8 weeks window to the date of randomization.
2. Biomaterial research: detailed information about biomaterial collection, handling and shipment will be sent to participating sites in a separate document called “Manual of Biological Material Collection”.
3. Blood for translational research will be collected at week 25 unless the patient has clinical Radiological progression before. In the occurrence of disease progression prior to week 25, blood collection for translational research must be taken at this point.
4. Data collection from the Follow Up period: Year 1 and Year 2 must be considered starting from Cycle 1 Day 1;

5. Screening period is defined as up to 28 days starting from the date informed consent was signed.
6. Hepatic evaluations and AE assessment scheduled fo weeks 3, 7 and 11 must be performed only for patients randomized to arms 1 and 3, meaning: participants that are receiving abiraterone acetate.
7. The safety visit must be performed 30 days after the actual last dose of treatment taken by the patient (considering the possibility of the extension phase).

8.1 Information to be collected on screening failures

All screening failure patients will have their demographic data and reasons for not been eligible in this study collected. All exams performed during screening must also be included in the eCRF.

8.2 Patient demographics/other baseline characteristics

All patients will have their demographic data collected, including, but not limited to: date of birth, gender, marital status and self-reported race.

8.3 Treatments

All study therapy must be recorded on the eCRF, including tablets accountability. Any medical, surgical or other treatments for any medical condition must be collected in the eCRF.

Compliance will be assessed by the investigator and/or study personnel at each visit using pill counts and information provided by the caregiver. This information should be captured in the source document at each visit.

Patients are allowed to continue study treatment beyond the 25-week assessment at the discretion of the investigator until objective or clinical disease progression, or occurrence of an unacceptable safety or tolerability issue.

Patients will be discontinued from the study treatment due to progression (radiographic per RECIST 1.1 and/or symptomatic +/- biochemical according to PCWG3 criteria), adverse event or patient withdrawal.

8.4 Efficacy

All patients will have their lesions followed by computed tomography and bone scan, according to RECIST 1.1 criteria.[26] PSA test will be collected at screening within before 7 days from randomization, cycle 2 and monthly until PSA confirmation visit.

8.5 Safety

All patients will have the adverse events followed and registered by the investigator, according to CTCAE v4 criteria. Data will be collected from screening to cycle 7 monthly, and on Safety visit.

8.5.1 Physical examination

Physical examination will be performed at screening and monthly until C7 and on Safety visit to collect weight. Height will be collected at screening period only.

8.5.2 Vital signs

Vital signs will be verified at screening and monthly until C7 and on Safety visit to collect blood pressure. Heart rate, respiratory rate and body temperature will be collected at screening period only.

8.5.3 Performance status

Performance status will be measure at every applicable visit using ECOG system.

8.5.4 Laboratory evaluations

Hematology (hemoglobin, white blood cell count, neutrophil count and platelet count) will be performed at screening, cycle 3, cycle 5, cycle 7 and safety visits.

Biochemistry (LDH, potassium, creatinine, total bilirubin, AST, ALT, albumin and fasting glucose) will be performed at screening, cycles 2 to 7 and safety visit.

Additional hepatic evaluations (albumin, total bilirubin, AST and ALT) must be performed on weeks 3, 7 and 11 for patients randomized to arms 1 and 3, meaning: participants that are receiving abiraterone acetate.

PSA and testosterone levels will be performed at screening visit (within 7 days before randomization), on cycles 2 to 7. They will also be collected at a PSA confirmation visit to take place on week 29 (± 7 days) in two case-scenarios.

8.5.5 Radiological examinations

Patients will undergo computed tomography of thorax, abdomen and pelvis at week 25. Bone scan will be performed at safety visit. Results from year 1 and 2 will be collected from medical chart and compared to patient's prior exams that will be used at screening. Response evaluation will be beased on RECIST 1.1 criteria.[26]

Additional CT scans may be requested if patient presents clinical signs or symptoms of disease progression.

8.5.6 Cardiac assessments

All patients will undergo a 12-lead electrocardiogram and MUGA (multiple gated acquisition) scan or echocardiogram at baseline and at week 25.

8.6 Patient-reported outcomes

Patient-reported Outcomes (adapted BPI-SF and FACT-P questionnaires) will be collected monthly from enrollment to week 25.

9 Study completion

The patients who are benefiting from the study treatment, regardless of the treatment arm they have been assigned to, at week 25 are allowed to continue receiving this medication in an extension phase. These patients will be followed at 2 different times: 12 and 24 months after study treatment initiation. Biochemical and radiological progression and survival status data will be collected from the medical chart (Cycle 1 Day 1).

10 Safety monitoring

10.1 Adverse events

10.1.1 Adverse Events Reporting

Timely, accurate, and complete reporting and analysis of safety information from clinical studies are crucial for the protection of subjects, investigators, and the sponsor, and are mandated by regulatory agencies worldwide. The sponsor has established Standard Operating Procedures in conformity with regulatory requirements worldwide to ensure appropriate reporting of safety information; all clinical studies conducted by the sponsor or its affiliates will be conducted in accordance with those procedures.

Method of Detecting Adverse Events and Serious Adverse Events

Care will be taken not to introduce bias when detecting adverse events or serious adverse events. Open-ended and nonleading verbal questioning of the subject is the preferred method to inquire about adverse event occurrence. For some studies, subjects are not always able to provide valid verbal responses to open-ended questions. In these circumstances, laboratory tests will be performed in order to identify some of these events.

Solicited Adverse Events

Solicited adverse events are predefined local and systemic events for which the subject is specifically questioned.

Unsolicited Adverse Events

Unsolicited adverse events are all adverse events for which the subject is specifically not questioned.

10.1.2 Adverse Event Definitions and Classifications

Adverse Event

An adverse event is any untoward medical occurrence in a clinical study subject administered a medicinal (investigational or non-investigational) product. An adverse event does not necessarily have a causal relationship with the treatment. An adverse event can therefore be any unfavorable and unintended sign (including an abnormal finding), symptom,

or disease temporally associated with the use of a medicinal (investigational or non-investigational) product, whether or not related to that medicinal (investigational or non-investigational) product. (Definition per International Conference on Harmonization [ICH])

This includes any occurrence that is new in onset or aggravated in severity or frequency from the baseline condition, or abnormal results of diagnostic procedures, including laboratory test abnormalities.

Note: The sponsor collects adverse events starting with the signing of the ICF.

Serious Adverse Event

A serious adverse event based on ICH and EU Guidelines on Pharmacovigilance for Medicinal Products for Human Use is any untoward medical occurrence that at any dose:

- Results in death
- Is life-threatening (The subject was at risk of death at the time of the event. It does not refer to an event that hypothetically might have caused death if it were more severe.)
- Requires inpatient hospitalization or prolongation of existing hospitalization
- Results in persistent or significant disability/incapacity
- Is a congenital anomaly/birth defect
- Is a suspected transmission of any infectious agent via a medicinal product
- Is Medically Important*

*Medical and scientific judgment should be exercised in deciding whether expedited reporting is also appropriate in other situations, such as important medical events that may not be immediately life threatening or result in death or hospitalization but may jeopardize the subject or may require intervention to prevent one of the other outcomes listed in the definition above. These should usually be considered serious.

If a serious and unexpected adverse event occurs for which there is evidence suggesting a causal relationship between the study drug and the event (eg, death from anaphylaxis), the event must be reported as a serious and unexpected suspected adverse reaction even if it is a component of the study endpoint (eg, all-cause mortality).

Unlisted (Unexpected) Adverse Event/Reference Safety Information

An adverse event is considered unlisted if the nature or severity is not consistent with the applicable product reference safety information. For apalutamide, the expectedness of an adverse event will be determined by whether or not it is listed in the Investigator's Brochure. For abiraterone acetate, androgen deprivation therapy and prednisone, with a marketing authorization, the expectedness of an adverse event will be determined by whether or not it is listed in the package insert.

Adverse Event Associated With the Use of the Drug

An adverse event is considered associated with the use of the drug if the attribution is not related, doubtful, possible, probable, or very likely.

10.1.3 Attribution Definitions

● **Not Related**

An adverse event that is not related to the use of the drug.

● **Doubtful**

An adverse event for which an alternative explanation is more likely, eg, concomitant drug(s), concomitant disease(s), or the relationship in time suggests that a causal relationship is unlikely.

● **Possible**

An adverse event that might be due to the use of the drug. An alternative explanation, eg, concomitant drug(s), concomitant disease(s), is inconclusive. The relationship in time is reasonable; therefore, the causal relationship cannot be excluded.

● **Probable**

An adverse event that might be due to the use of the drug. The relationship in time is suggestive (eg, confirmed by dechallenge). An alternative explanation is less likely, eg, concomitant drug(s), concomitant disease(s).

● **Very Likely**

An adverse event that is listed as a possible adverse reaction and cannot be reasonably explained by an alternative explanation, eg, concomitant drug(s), concomitant disease(s). The relationship in time is very suggestive (eg, it is confirmed by dechallenge and rechallenge).

10.2 Special Reporting Situations

Safety events of interest on a sponsor study drug that may require expedited reporting or safety evaluation include, but are not limited to:

● Overdose of a sponsor study drug

● Suspected abuse/misuse of a sponsor study drug

● Accidental or occupational exposure to a sponsor study drug

● Any failure of expected pharmacologic action (ie, lack of effect) of a sponsor study drug.

● Unexpected therapeutic or clinical benefit from use of a sponsor study drug.

● Medication error involving a sponsor product (with or without subject/patient exposure to the sponsor study drug, eg, name confusion)

● Exposure to a sponsor study drug from breastfeeding

Special reporting situations should be recorded in the CRF. Any special reporting situation that meets the criteria of a serious adverse event should be recorded on the serious adverse event page of the CRF.

10.3 Procedures

10.3.1 All Adverse Events

All adverse events and special reporting situations, whether serious or non-serious, will be reported from the time a signed and dated ICF is obtained until completion of the subject's last study-related procedure, which may include contact for follow-up of safety. Serious adverse events, including those spontaneously reported to the investigator within 30 days after the last dose of study drug, must be reported using the Serious Adverse Event Form. The sponsor will evaluate any safety information that is spontaneously reported by an investigator beyond the time frame specified in the protocol.

All adverse events, regardless of seriousness, severity, or presumed relationship to study drug, must be recorded using medical terminology in the source document and the CRF. Whenever possible, diagnoses should be given when signs and symptoms are due to a common etiology (eg, cough, runny nose, sneezing, sore throat, and head congestion should be reported as "upper respiratory infection"). Investigators must record in the CRF their opinion concerning the relationship of the adverse event to study therapy. All measures required for adverse event management must be recorded in the source document and reported according to sponsor instructions.

The sponsor assumes responsibility for appropriate reporting of adverse events to the regulatory authorities. The sponsor will also report to the investigator (and the head of the investigational institute where required) all suspected unexpected serious adverse reactions (SUSARs). The investigator (or sponsor where required) must report SUSARs to the appropriate Independent Ethics Committee/Institutional Review Board (IEC/IRB) that approved the protocol unless otherwise required and documented by the IEC/IRB.

For all studies with an outpatient phase, including open-label studies, the subject must be provided with a "wallet (study) card" and instructed to carry this card with them for the duration of the study indicating the following:

● Study number

● Statement, in the local language(s), that the subject is participating in a clinical study

● Investigator's name and 24-hour contact telephone number

● Local sponsor's name and 24-hour contact telephone number (for medical staff only)

● Site number

● Subject number

● Any other information that is required to do an emergency breaking of the blind

10.3.2 Serious Adverse Events

All serious adverse events occurring during the study must be reported to the appropriate sponsor contact person by study-site personnel within 24 hours of their knowledge of the event.

Information regarding serious adverse events will be transmitted to the sponsor using the Serious Adverse Event Form, which must be completed and signed by a physician from the study site, and transmitted to the sponsor within 24 hours. The initial and follow-up reports of a serious adverse event should be made by facsimile (fax) or email.

All serious adverse events that have not resolved by the end of the study, or that have not resolved upon discontinuation of the subject's participation in the study, must be followed until any of the following occurs:

● The event resolves

● The event stabilizes

● The event returns to baseline, if a baseline value/status is available

● The event can be attributed to agents other than the study drug or to factors unrelated to study conduct

● It becomes unlikely that any additional information can be obtained (subject or health care practitioner refusal to provide additional information, lost to follow-up after demonstration of due diligence with follow-up efforts)

● Suspected transmission of an infectious agent by a medicinal product will be reported as a serious adverse event. Any event requiring hospitalization (or prolongation of hospitalization) that occurs during the course of a subject's participation in a study must be reported as a serious adverse event, except hospitalizations for the following:

● Hospitalizations not intended to treat an acute illness or adverse event (eg, social reasons such as pending placement in long-term care facility)

● Surgery or procedure planned before entry into the study (must be documented in the CRF). Note: Hospitalizations that were planned before the signing of the ICF, and where the underlying condition for which the hospitalization was planned has not worsened, will not be considered serious adverse events. Any adverse event that results in a prolongation of the originally planned hospitalization is to be reported as a new serious adverse event.

● For convenience the investigator may choose to hospitalize the subject for the duration of the treatment period.

● Disease progression should not be recorded as an adverse event or serious adverse event term; instead, signs and symptoms of clinical sequelae resulting from disease progression/lack of efficacy will be reported if they fulfill the serious adverse event definition (refer to Section 10.1.2, Adverse Event Definitions and Classifications)

10.3.3 Pregnancy

All initial reports of pregnancy in partners of study subjects must be reported to the sponsor by the study-site personnel within 24 hours of their knowledge of the event using the appropriate pregnancy notification form. Abnormal pregnancy outcomes (eg, spontaneous abortion, fetal death, stillbirth, congenital anomalies, ectopic pregnancy) are considered serious adverse events and must be reported using the Serious Adverse Event Form.

If a subject's partner becomes pregnant during the study, a determination regarding study drug discontinuation must be made by the investigator in consultation with the sponsor. Because the study drug may have an effect on sperm, and because the effect of the study drug on sperm is unknown, pregnancies in partners of male subjects included in the study will be reported as noted above.

Follow-up information regarding the outcome of the pregnancy and any postnatal sequelae in the infant will be required.

10.3.4 Contacting Sponsor Regarding Safety

The names (and corresponding telephone numbers) of the individuals who should be contacted regarding safety issues or questions regarding the study are listed in the Contact Information page(s), which will be provided as a separate document.

10.4 Product Quality Complaint Handling

A product quality complaint (PQC) is defined as any suspicion of a product defect related to manufacturing, labeling, or packaging, ie, any dissatisfaction relative to the identity, quality, durability, or reliability of a product, including its labeling or package integrity. A PQC may have an impact on the safety and efficacy of the product. Timely, accurate, and complete reporting and analysis of PQC information from studies are crucial for the protection of subjects, investigators, and the sponsor, and are mandated by regulatory agencies worldwide. The sponsor has established procedures in conformity with regulatory requirements worldwide to ensure appropriate reporting of PQC information; all studies conducted by the sponsor or its affiliates will be conducted in accordance with those procedures.

10.4.1 Procedures

All initial PQCs must be reported to the sponsor by the study-site personnel within 24 hours after being made aware of the event.

If the defect is combined with a serious adverse event, the study-site personnel must report the PQC to the sponsor according to the serious adverse event reporting timelines. A sample of the suspected product should be maintained for further investigation if requested by the sponsor.

10.4.2 Contacting Sponsor Regarding Product Quality

The names (and corresponding telephone numbers) of the individuals who should be contacted regarding product quality issues are listed in the Contact Information page(s), which will be provided as a separate document.

11 Data review and data management

11.1 Site monitoring

The sponsor will perform on-site monitoring visits as frequently as necessary. The monitor will record dates of the visits in a study-site visit log that will be kept at the study site. The first post-initiation visit will be made as soon as possible after enrollment has begun. At these visits, the monitor will compare data entered into the eCRFs with the hospital or clinic records (source documents); a sample may be reviewed. The nature and location of all source documents will be identified to ensure that all sources of original data required to complete the eCRF are known to the sponsor and study-site personnel and are accessible for verification by the sponsor study-site contact. If electronic records are maintained at the study site, the method of verification must be discussed with the study-site personnel.

Direct access to source documentation (medical records) must be allowed for the purpose of verifying that the data recorded in the eCRF are consistent with the original source data. Findings from this review of eCRFs and source documents will be discussed with the study-site personnel. The sponsor expects that, during monitoring visits, the relevant study-site personnel will be available, the source documentation will be accessible, and a suitable environment will be provided for review of study-related documents. The monitor will meet with the investigator on a regular basis during the study to provide feedback on the study conduct.

In addition to on-site monitoring visits, remote contacts or remote monitoring may occur, in the impossibility of on-site monitoring. During remote monitoring, the monitor will review the data by sharing it by videoconferences, electronic systems or electronic platforms and remote verification of the data in the eCRF. It is expected that during these remote contacts, study-site personnel will be available to provide an update on the progress of the study at the site and during remote monitoring for the provision of the data to be reviewed.

.

11.2 Data collection

Subject Identification, Enrollment, and Screening Logs
The investigator agrees to complete a subject identification and enrollment log to permit easy identification of each subject during and after the study. This document will be reviewed by the sponsor study-site contact for completeness.
The subject identification and enrollment log will be treated as confidential and will be filed by the investigator in the study file. To ensure subject confidentiality, no copy will be made. All reports and communications relating to the study will identify subjects by subject identification and date of birth. In cases where the subject is not randomized into the study, the date seen and date of birth will be used.
The investigator must also complete a subject screening log, which reports on all subjects who were seen to determine eligibility for inclusion in the study.

Source Documentation
At a minimum, source documentation must be available for the following to confirm data collected in the eCRF: subject identification, eligibility, and study identification; study discussion and date of signed informed consent; dates of visits; results of safety and efficacy parameters as required by the protocol; record of all adverse events and follow-up of adverse events; concomitant medication; drug receipt/dispensing/return records; study drug administration information; and date of study completion and reason for early discontinuation of study drug or withdrawal from the study, if applicable.
In addition, the author of an entry in the source documents should be identifiable.
At a minimum, the type and level of detail of source data available for a subject should be consistent with that commonly recorded at the study site as a basis for standard medical care. Specific details required as source data for the study will be reviewed with the investigator before the study and will be described in the monitoring guidelines (or other equivalent document).

Case Report Form Completion
Case report forms are provided for each subject in electronic format.
Electronic Data Capture (eDC) will be used for this study. The study data will be transcribed by study-site personnel from the source documents onto an eCRF, and transmitted in a secure manner to the sponsor within the timeframe agreed upon between the sponsor and the study site.
Worksheets may be used for the capture of some data to facilitate completion of the eCRF. Any such worksheets will become part of the subject's source documentation. All data relating to the study must be recorded in eCRFs prepared by the sponsor. Data must be entered into eCRFs in English. Study site personnel must complete the eCRF as soon as possible after a subject visit, and the forms should be available for review at the next scheduled monitoring visit.
All eCRF entries, corrections, and alterations must be made by the investigator or other authorized study-site personnel. If necessary, queries will be generated in the eDC tool. The investigator or study-site personnel must adjust the eCRF (if applicable) and complete the query.

If corrections to an eCRF are needed after the initial entry into the eCRF, this can be done in 3 different ways:
• Study site personnel can make corrections in the eDC tool at their own initiative or as a response to an auto query (generated by the eDC tool).
• Site monitor can generate a query for resolution by the study-site personnel.
• Clinical data manager can generate a query for resolution by the study-site personnel.

11.3 Database management and quality control

All data will be managed and analysed by LACOG staff and investigators.
Steps to be taken to ensure the accuracy and reliability of data include the selection of qualified investigators and appropriate study sites, review of protocol procedures with the investigator and study-site personnel before the study, and periodic monitoring visits by the sponsor, and direct transmission of clinical laboratory data from a central laboratory into the sponsor's data base. Written instructions will be provided for collection, handling, storage, and shipment of samples.
Guidelines for eCRF completion will be provided and reviewed with study-site personnel before the start of the study. The sponsor or its designee will review eCRFs for accuracy and completeness during on-site monitoring visits and after transmission to the sponsor; any discrepancies will be resolved with the investigator or designee, as appropriate. After upload of the data into the study database they will be verified for accuracy and consistency with the data sources.

12 Statistical methods and data analysis

All statistical analyses specified in this protocol will be conducted using SAS version 9.4, and a significance level of 5%.

12.1 Sample Size

For the primary endpoint (PSA below 0.2ng/mL at week 25), and using Fleming one-stage method, a sample size of 38 participants per arm would allow 80% power to reject a PSA undetectable rate (defined as ≤ 0.2 ng/mL) of 45% or less at a 5% significance level, with an expected PSA response rate for each of the three arms of about 65%.[25,27] Allowing a 10% dropout, we planned to enroll 42 participants per arm. In total the study will enroll 126 patients.

Eligible patients will be stratified in randomization according to performance status (ECOG 0-1 vs 2) and metastatic disease (yes vs. no).

Probably the study will have a power lower than 80% in the most of secondary analyses. For that reason, a power calculation will be performed for each secondary association, especially for those with p-value < 0.05.
~~All the efficacy analyses will take place using the intention to treat (ITT) population.~~ No

interim analysis for futility is planned.

New amendment text (refers strikethrough sentence): "Intention to treat (ITT) population and modified ITT (mITT) population analyses were performed for the endpoints. For the primary endpoint an aditonal sensitivity analysis was performed and missing PSA data at week 25 were labeled as failures."

12.2 Populations for analysis

~~ITT Population: The ITT population includes all randomized subjects classified according to their assigned treatment group, regardless of the actual treatment received. Subject disposition and efficacy analyses will be performed on data from the ITT population.~~

~~Safety Population: The safety population includes all subjects who received at leas 1 dose of study drug.~~

~~Patient-reported Outcomes Population [PRO]: The PRO population includes randomized subjects who have completed at least the baseline assessment of BPI-SF and FACT-P questionnaires.~~

New protocol amendment text (refers strikethrough sentence):

"ITT Population: *The ITT population includes all randomized subjects classified according to their assigned treatment group, regardless of the actual treatment received.*

- *Baseline patient characteristics*
- *Maximum PSA declines and overall PSA change from baseline up to week 25*
- *Hormonal levels during treatment*

Modified ITT Population*: The modified ITT population includes only randomized subjects with evaluable PSA at week 25.*

- *Primary endpoint: proportion of patients who achieve an undetectable PSA level, defined as ≤ 0.2 ng/mL at week 25 in each of three arms.*
- *PSA decline ≥ 50 and ≥ 80% at week 25*
- *PSA progression rate at week 25 (PCWG3 criteria)*

Sensitivity analysis: *It was performed a sensitivity analysis for the primary endpoint analysis. All randomized patients were included in this analysis. Patients with missing PSA data at week 25 were labeled as failures.*

- *Primary endpoint: proportion of patients who achieve an undetectable PSA level, defined as ≤ 0.2 ng/mL at week 25 in each of three arms.*

To evaluate the radiographic progression rate at week 25, only patients who had the tumor assessment images evaluable at week 25 were considered.

***Safety Population:** The safety population includes all subjects who received at least 1 dose of study drug.*

***Patient-reported Outcomes Population [PRO]**: The PRO population includes randomized subjects who have completed the baseline assessment and at least one postbaseline assessment of BPI-SF and FACT-P questionnaires."*

12.3 Efficacy analyses

Demographics and baseline disease characteristics will be analyzed using descriptive statistics. Subject's age, height, weight and other quantitative baseline characteristics will be summarized by number of patients with available information in each characteristic, mean, standard deviation, median, minimum, maximum and quartiles, when appropriate. Categorical variables (age group, gender, race, histology and others) will be summarized using frequency tabulations (count and percent).

12.3.1 Analysis of primary endpoint

The proportion of patiens in each arm who achieves a PSA level ≤ 0.2 ng/mL at week 25 will be evaluated as the number of patients with an undetectable PSA level at week 25 divided by the number of patients randomized for each group, according to ECOG status and disease volume.

12.3.2 Analysis of secondary endpoints

Secondary objectives related to hormonal levels~~, bone density,~~ safety profile and quality among the three arms will be summarized descriptively.

The proportion of patients with a PSA response at week 25, calculated as the number of patients with PSA response (≥ 50% and ≥ 80% PSA decline from baseline) at week 25 divided by the number of randomized patients in each group, will be presented as the percentage of patients responding with the corresponding 95% CI based in the exact binomial distribution. PSA progression rate will be evaluated according to PCWG3 criteria at week 25 and 53 from time to randomization. And the maximum PSA declines and overall PSA change from baseline up to week 25 and up to week 53 will be summarized descriptively among the three arms.

~~Radiographic progression-free survival (rPFS) based on Prostate Cancer Working Group 3 and RECIST 1.1 will be assessed from the randomization to week 25.~~

New protocol amendment (refers to strikethrough text): *Radiographic progression rate at week 25 according to RECIST 1.1 criteria. Only patients who had the tumor assessment images evaluable at week 25 and with overall response able to assess were evaluated.*

Hormonal levels testosterone during treatment ~~and bone density~~ will be described ~~and compared between~~ arms and correlated with primary endpoint.

2-year overall survival rate is defined as percentage of people in the study who are still alive 2 years after the date of initiation (first dose) of treatment

Time-to-next treatment is measured from the date of initiation (first dose) of treatment, to the date of initiation of the next line of therapy.

Time-to-event endpoint will be estimated by Kaplan-meier method and compared by stratified log-rank test or Cox regression method.

Dichotomic data will be analysed using Fischer's exact test or Chi-squared test. If necessary, other methods for categorical data may also be applied as appropriate.

12.3.3 Patient-reported outcomes

Descriptive statistics of each PRO scale score from the FACT-P and BFI-SF at baseline and follow-up assessments will be summarized by treatment groups. Only question number 3 will be used from BFI-SF questionnaire; time to degradation in each scale will be analyzed using Kaplan-Meier method and stratified Cox proportional hazard model. Additional analyses may be carried out, if appropriate. Analyses details will be included in the statistical analysis plan.

12.4 Safety analyses

Subjects who receive at least 1 dose of study drug will be analyzed for safety. The safety parameters to be evaluated are the incidence, intensity, and type of adverse events, clinically significant changes in the subject's physical examination findings, vital signs measurements, and clinical laboratory results. Exposure to study drug and reasons for discontinuation of study treatment will be tabulated.

12.4.1 Adverse events (AE)

The verbatim terms used in the eCRF by investigators to identify adverse events (AEs) will be coded using the most current Medical Dictionary for Regulatory Activities (MedDRA) and will be graded according to the latest version of the NCI-CTCAE. Treatment-emergent AEs are AEs that occur or worsen on or after first dose of study drug through 28 days after the last dose of study drug and will be included in the analysis. Adverse events will be summarized by system organ class and preferred term, and will be presented overall and by treatment group. Serious adverse events and deaths will be provided in a listing. All AEs resulting in discontinuation, dose modification, dosing interruption, or treatment delay of study drug will also be listed and tabulated by preferred term.
New additional protocol amendment text: "Treatment-related adverse events are AEs that occur or worsen on or after first dose of study drug through 28 days after the last dose of

study drug and were considered related to study drug. Multiple occurrences of the same event were counted once at the maximum severity.”

12.4.2 Laboratory abnormalities

Clinical laboratory test results will be collected from Screening and through 28 days after last dose of study drug. Laboratory data will be summarized by type of laboratory test. Parameters with predefined NCI-CTCAE toxicity grades will be summarized. Change from baseline to the worst AE grade experienced by the subject during the study will be provided as shift tables.

12.4.3 Other safety data

Other safety data will be summarized descriptively.

12.4.4 Vital Signs

Descriptive statistics of blood pressure (systolic and diastolic) values and changes from baseline will be summarized at each scheduled time point. Descriptive statistics of other vital signs at baseline will also be summarized. The number and percentage of subjects with values beyond clinically important limits will be summarized.

12.5 Resource utilization

Medical resource utilization will be descriptively summarized by treatment group. Results of these analyses will be reported separately and will not be a part of the clinical study report.

12.6 Treatment compliance

Each arm will be summarized according to the planned dose. For those patients unable to tolerate the protocol-specified dosing schedule, dose adjustments will be analysed, if necessary. Also, the number of patients analysed in each arm will be checked, once patients withdraw prematurely are possible (the reason for withdraw will be summarized too).

13 Translational Research

This study will collect biological material from patients and create a biorepository for translational research projects. The objective of this repository is to provide material for the planned exploratory biomarker research, such as analysis of relevant biomarkers that could serve as prognostic, predictive, pharmacodynamic and/or mechanisms of resistance. The biorepository will comply with the current regulations in Brazil. The samples will be handled in a manner that protects each patient’s privacy and confidentiality. Biospecimens will be used for the purposes described in the protocol and informed consent document. Biospecimens will be stored for ten years to allow for research. Patients may withdraw their consent for the use of their banked biospecimens at any time by making a request to the investigator. In this case, any remaining biospecimens will be destroyed, but data already generated from biospecimens will continue to be available to protect the integrity of existing

analyses.
Detailed information about biomaterial collection, handling and shipment will be sent to participating sites in a separate document called “Manual of Biological Material Collection”.

Type of biological material:

1. Tumor samples from FFPE blocks or slides will be requested from the archival site at the Cycle 1 Day 1 visit. These samples, when available, will be collected from all included subjects. Site team must make all efforts to have access to the tumor sample material (either FFPE block or slides). The laboratory manual for biological samples with standard operation procedures for tumor block or slides collection, handling and shipment will be sent by LACOG separately.

2. Blood samples will be collected from all subjects at Cycle 1 Day 1, Cycle 7 Day 1 or at the time of disease progression and on Years 1 and 2. The laboratory manual for biological samples with standard operating procedures for blood collection, handling and shipment will be sent by LACOG separately.

Tumor and blood biomarker analyses are dependent upon the availability of appropriate biomarker assays and may be deferred or not performed, if during or at the end of the study, it becomes clear that the analysis will have no scientific value, or there are not enough samples or not enough responders to allow for adequate biomarker evaluation. In the event the study is terminated early or does not reach a positive primary endpoint, completion of biomarker assessments is based on justification and intended utility of the data.

Translational research projects:

The planned exploratory biomarker research might be able to identify subpopulations of patients that may have differential benefits from the study treatment and may shed light into the mechanisms of resistance and the influence of sequencing of treatment on outcomes. To identify biomarkers, we will collect biospecimens (blood components, tumor material, etc.) to support analyses of cellular components (e.g., protein, DNA, RNA, metabolites) and other circulating molecules.

Exploratory analyses will include but are not limited to the following analyses:

Genomic analyses to evaluate whether genetic variations of the study population may influence response to the treatment might be performed. This research contributes to the understanding of genetic determinants of treatment safety and efficacy. Whole exome sequencing, genome sequencing, SNP analyses, genetic tumor analyses, transcriptome, epigenetic analyses might be performed to verify possible genes candidates that predispose patients to treatment benefit, or to the development of adverse events related

to treatment. Note that in order to understand tumor-specific mutations, it might be necessary to compare tumor genome with the germline genome.

Correlation of HSD3B1 (1245C) polymorphism with PSA level ≤0.2 ng/mL at week 25, with PFS at 2 years, with testosterone levels and dehydroepiandrosterone (DHEA-S) levels during therapy, and with PFS at 2 years on apalutamide monotherapy arm.

Protein biomarkers and RNA/micro-RNA analyses might be performed for analyses of the pharmacodynamics effects of study treatment and to explore candidate predictive molecular signatures associated with response and resistance. MicroRNAs (miRNAs) have been widely studied in prostate cancer and are small noncoding RNAs that regulate the expression of protein-coding genes by modulating both mRNA stability and translation.[29] Alteration of miRNA expression levels can alter cell function and induce cellular transformation leading to cancer. A number of dysregulated miRNAs have been associated with different prostate cancer stages and some miRNAs are consistently dysregulated at early and advanced stages of disease or associated with more aggressive disease.[30] Considering that some miRNAs are androgen controlled, it is anticipated that dysregulated miRNAs patterns in baseline samples may predict response to drugs such as abiraterone acetate and apalutamide. Changes in miRNA expression levels from baseline may allow elucidation of mechanisms leading to resistance. Correlation of molecular biomarkers from baseline tumor tissue and blood samples with PSA level ≤0.2 ng/mL at week 25, with best PSA response during therapy, and with Progression-Free Survival (PFS) at 2 years.

Dysregulation of steroid synthesis has been previously reported in xenograft models and evaluation of steroid profiles may complement the miRNA studies. Herein, we plan to analyze dysregulated steroid transcript levels by gene expression profiling to confirm the predictive profiles found in these previously reported studies. The advent of new agents represented by abiraterone acetate and apalutamide, which target adrenal or intraprostatic androgen biosynthesis and AR signaling, respectively, has retrieved interest in androgen levels during therapy to understand the mecanisms of resistance of prostate cancer after androgen deprivation therapy or homone therapy in hormone naive patients.[31] Comparison of the landscape of emerging molecular alterations and androgen-receptors aberrations in apalutamide monotherapy (high-testosterone regimen) vs. AAP+ADT and AAP+APA (low-testosterone regimens).

Circulating tumor DNA (ctDNA), circulating tumor RNA (ctRNA), and circulating tumor cells (CTC) analyses might be performed using blood samples (including plasma) to explore predictive markers of response to study treatment and to understand potential mechanisms of acquired resistance. Correlatetion of ctDNA levels with PSA level ≤0.2 ng/mL at week 25, with PFS at 2-years, and with PFS at 2 years.

Blood samples might be used for isolation of peripheral blood mononuclear cells (PBMCs). The PBMC samples can be used for immunophenotyping or characterization of subsets of immune cells, including, but not limited to, T cells, NK cells or subpopulations of the immune cell types mentioned previously. Tumor and blood samples from this study may undergo proteomic and/or immunohistochemistry (IHC) analyses of single markers or using multiplex

panels. Therefore, tumor tissue may be analyzed using a variety of platforms that could include, but are not limited to, immunoassays, liquid chromatography/mass spectrometry. This approach could identify novel protein biomarkers that could aid in patient selection for therapy.

~~Biomarker studies on the FFPE blocks or slides of tumor samples may include immunohistochemistry analyses, global miRNA profiling, tumor gene-expression profiling, and somatic mutational analyses.~~

~~MicroRNAs (miRNAs) have been widely studied in prostate cancer and are small noncoding RNAs that regulate the expression of protein-coding genes by modulating both mRNA stability~~
~~and translation.[29] Alteration of miRNA expression levels can alter cell function and induce cellular transformation leading to cancer. A number of dysregulated miRNAs have been associated with different prostate cancer stages and some miRNAs are consistently dysregulated at early and advanced stages of disease or associated with more aggressive disease.[30] Considering that some miRNAs are androgen controlled, it is anticipated that dysregulated miRNAs patterns in baseline samples may predict response to drugs such as abiraterone acetate and apalutamide. Changes in miRNA expression levels from baseline may allow elucidation of mechanisms leading to resistance.~~

~~Dysregulation of steroid synthesis has been previously reported in xenograft models and evaluation of steroid profiles may complement the miRNA studies. Herein, we plan to analyze dysregulated steroid transcript levels by gene expression profiling to confirm the predictive profiles found in these previously reported studies. The advent of new agents represented by abiraterone acetate and apalutamide, which target adrenal or intraprostatic androgen biosynthesis and AR signaling, respectively, has retrieved interest in androgen levels during therapy to understand the mecanisms of resistance of prostate cancer after androgen deprivation therapy or homone therapy in hormone naive patients.[31]~~

~~Data collected from this study will be compared to data obtained from prior studies in advanced/metastatic hormone sensitive prostate cancer to identify miRNA and GEP profiles that correlate with response (or primary resistance) to abiraterone and apalutamide. The biomarker results from this study will then be used to inform future studies of anti-androgen therapies possibly leading to product differentiation by selection of responsive subjects.~~
~~miRNAs have also been identified that correlate with high risk prostate cancer. Since it is difficult to histologically distinguish high risk from benign disease and because PSA velocity and doubling time may be an unreliable measure of disease aggression, we plan to investigate~~
~~whether miRNA profiles may better define high risk prostate cancer in the early advanced/metastatic disease setting. This data may then be utilized in selection of high risk patients in future studies if these previously reported miRNA profiles are confirmed and found to be more sensitive than conventional clinical estimates of high risk disease.~~

14 Study-specific materials

The investigator will be provided with the following supplies:

• Investigator Brochure

• Pharmacy manual

• Biological material collection for Translational Research manual

15 Ethical aspects

15.1 Study-Specific Design Considerations

Potential subjects will be fully informed of the risks and requirements of the study and, during the study, subjects will be given any new information that may affect their decision to continue participation. They will be told that their consent to participate in the study is voluntary and may be withdrawn at any time with no reason given and without penalty or loss of benefits to which they would otherwise be entitled. Only subjects who are fully able to understand the risks, benefits, and potential adverse events of the study, and provide their consent voluntarily will be enrolled.

The blood volume to be collected is approximately 8 to 10.0 mL per study visit. As rPFS is a secondary endpoint, scheduled imaging is incorporated in the protocol. The timing of imaging is designed to capture progression events yet not to have patients be overexposed to radiation.

Subjects will receive prednisone 10mg/day. The required use of prednisone in combination with abiraterone acetate is to help mitigate the symptoms of mineralocorticoid excess caused by CYP17 inhibition, which is the mechanism of action of abiraterone acetate. Data from early Phase 1/2 studies with abiraterone acetate in patients with mCRPC demonstrate that dexamethasone 0.5mg once daily (equivalent to prednisone dose of 3.33 mg once daily) was effective in mitigating the mineralocorticoid effects of abiraterone acetate.3

15.2 Regulatory Ethics Compliance

15.2.1 Investigator Responsibilities

The investigator is responsible for ensuring that the clinical study is performed in accordance with the protocol, current ICH guidelines on Good Clinical Practice (GCP), and applicable regulatory and country-specific requirements.

Good Clinical Practice is an international ethical and scientific quality standard for designing, conducting, recording, and reporting studies that involve the participation of human subjects. Compliance with this standard provides public assurance that the rights, safety, and well-being of study subjects are protected, consistent with the principles that originated in the Declaration of Helsinki, and that the clinical study data are credible.

15.2.2 Independent Ethics Committee or Institutional Review Board

Before the start of the study, the investigator (or sponsor where required) will provide the IEC/IRB with current and complete copies of the following documents:

- Final protocol and, when applicable, amendments
- Sponsor-approved informed consent form (and any other written materials to be provided to the subjects)
- Investigator's Brochure (or equivalent information) and amendments/addenda
- Sponsor-approved subject recruiting materials
- Information on compensation for study-related injuries or payment to subjects for participation in the study, when applicable
- Investigator's curriculum vitae or equivalent information (unless not required, as documented by the IEC/IRB)
- Information regarding funding, name of the sponsor, institutional affiliations, other potential conflicts of interest, and incentives for subjects
- Any other documents that the IEC/IRB requests to fulfill its obligation

This study will be undertaken only after the IEC/IRB has given full approval of the final protocol, any amendments, the informed consent form, applicable recruiting materials, and subject compensation programs, and the sponsor has received a copy of this approval. This approval letter must be dated and must clearly identify the IEC/IRB and the documents being approved.

During the study the investigator (or sponsor where required) will send the following documents and updates to the IEC/IRB for their review and approval, where appropriate:

- Protocol amendments
- Revision(s) to informed consent form and any other written materials to be provided to subjects
- When applicable, new or revised subject recruiting materials approved by the sponsor
- Revisions to compensation for study-related injuries or payment to subjects for participation in the study, when applicable
- New edition(s) of the Investigator's Brochure and amendments/addenda
- Summaries of the status of the study at intervals stipulated in guidelines of the IEC/IRB (at least annually)
- Reports of adverse events that are serious, unlisted/unexpected, and associated with the investigational drug
- New information that may adversely affect the safety of the subjects or the conduct of the study
- Deviations from or changes to the protocol to eliminate immediate hazards to the subjects
- Report of deaths of subjects under the investigator's care

• Notification when a new investigator is responsible for the study at the site

• Development Safety Update Report and Line Listings, where applicable

• Any other requirements of the IEC/IRB

For all protocol amendments (excluding the ones that are purely administrative, with no consequences for subjects, data or study conduct), the amendment and applicable informed consent form revisions must be submitted promptly to the IEC/IRB for review and approval before implementation of the change(s).

At least once a year, the IEC/IRB will be asked to review and reapprove this clinical study. The reapproval should be documented in writing (excluding the ones that are purely administrative, with no consequences for subjects, data, or study conduct).

At the end of the study, the investigator (or sponsor where required) will notify the IEC/IRB about the study completion.

15.2.3 Informed Consent

Each subject must give written consent according to local requirements after the nature of the study has been fully explained. The consent form must be signed before performance of any study-related activity. The consent form that is used must be approved by both the sponsor and by the reviewing IEC/IRB and be in a language that the subject can read and understand. The informed consent should be in accordance with principles that originated in the Declaration of Helsinki, current ICH and GCP guidelines, applicable regulatory requirements, and sponsor policy.

Before enrollment in the study, the investigator or an authorized member of the investigational staff must explain to potential subjects the aims, methods, reasonably anticipated benefits, and potential hazards of the study, and any discomfort participation in the study may entail. Subjects will be informed that their participation is voluntary and that they may withdraw consent to participate at any time. They will be informed that choosing not to participate will not affect the care the subject will receive for the treatment of his disease. Subjects will be told that alternative treatments are available should they refuse to take part and that such refusal will not prejudice future treatment. Finally, they will be told that the investigator will maintain a subject identification register for the purposes of long-term follow up when needed and that their records may be accessed by health authorities and authorized sponsor staff without violating the confidentiality of the subject, to the extent permitted by the applicable law(s) or regulations. By signing the informed consent form the subject is authorizing such access, and agrees to allow his or her study physician to recontact the subject for the purpose of obtaining consent for additional safety evaluations, and subsequent disease-related treatments or to obtain information about his survival status.

The subject will be given sufficient time to read the informed consent form and the opportunity to ask questions. After this explanation and before entry into the study, consent should be appropriately recorded by means of the subject's personally dated signature. After having obtained the consent, a copy of the informed consent form must be given to the subject. If the subject is unable to read or write, then an impartial witness should be present for the entire informed consent process (which includes reading and explaining all written

information) and should personally date and sign the informed consent form after the oral consent of the subject is obtained.

15.2.4 Privacy of Personal Data

The collection and processing of personal data from subjects enrolled in this study will be limited to those data that are necessary to fulfill the objectives of the study.

These data must be collected and processed with adequate precautions to ensure confidentiality and compliance with applicable data privacy protection laws and regulations. Appropriate technical and organizational measures to protect the personal data against unauthorized disclosures or access, accidental or unlawful destruction, or accidental loss or alteration must be put in place. Sponsor personnel whose responsibilities require access to personal data agree to keep the identity of study subjects confidential.

The informed consent obtained from the subject includes explicit consent for the processing of personal data and for the investigator to allow direct access to his or her original medical records for study-related monitoring, audit, IEC/IRB review, and regulatory inspection. This consent also addresses the transfer of the data to other entities and to other countries.

The subject has the right to request through the investigator access to his or her personal data and the right to request rectification of any data that are not correct or complete. Reasonable steps will be taken to respond to such a request, taking into consideration the nature of the request, the conditions of the study, and the applicable laws and regulations.

16 Administrative requirements

16.1 Protocol Amendments

Neither the investigator nor the sponsor will modify this protocol without a formal amendment by the sponsor. All protocol amendments must be issued by the sponsor, and signed and dated by the investigator. Protocol amendments must not be implemented without prior IEC/IRB approval, or when the relevant competent authority has raised any grounds for non-acceptance, except when necessary to eliminate immediate hazards to the subjects, in which case the amendment must be promptly submitted to the IEC/IRB and relevant competent authority.

Documentation of amendment approval by the investigator and IEC/IRB must be provided to the sponsor or its designee. When the change(s) involves only logistic or administrative aspects of the study, the IRB (and IEC where required) only needs to be notified.

During the course of the study, in situations where a departure from the protocol is unavoidable, the investigator or other physician in attendance will contact the appropriate sponsor representative (see Contact Information page(s)). Except in emergency situations, this contact should be made before implementing any departure from the protocol. In all cases, contact with the sponsor must be made as soon as possible to discuss the situation and agree on an appropriate course of action. The data recorded in the eCRF and source documents will reflect any departure from the protocol, and the source documents will describe this departure and the circumstances requiring it.

16.2 Regulatory Documentation

16.2.1 Regulatory Approval/Notification

This protocol and any amendment(s) must be submitted to the appropriate regulatory authorities in each respective country, when applicable. A study may not be initiated until all local regulatory requirements are met.

16.2.2 Required Prestudy Documentation

The following documents must be provided to the sponsor before shipment study drug to the investigational site:

• Protocol and any amendment(s), signed and dated by the principal investigator

• A copy of the dated and signed, written IEC/IRB approval of the protocol, amendments, informed consent form, any recruiting materials, and when applicable, subject compensation programs. This approval must clearly identify the specific protocol by title and number and must be signed by the chairman or authorized designee.

• Name and address of the IEC/IRB, including a current list of the IEC/IRB members and their function, with a statement that it is organized and operates according to GCP and the applicable laws and regulations. If accompanied by a letter of explanation, or equivalent, from the IEC/IRB, then a general statement may be substituted for this list. If an investigator or a member of the investigational staff is a member of the IEC/IRB, then documentation must be obtained to state that this person did not participate in the deliberations or in the vote/opinion of the study.

• Regulatory authority approval or notification, when applicable

• Signed and dated statement of investigator (eg, Form FDA 1572), when applicable

• Documentation of investigator qualifications (eg, curriculum vitae)

• Completed investigator financial disclosure form from the principal investigator, where required

• Signed and dated clinical trial agreement, which includes the financial agreement

• Any other documentation required by local regulations

The following documents must be provided to the sponsor before enrollment of the first subject:

• Completed investigator financial disclosure forms from all clinical subinvestigators

• Documentation of subinvestigator qualifications (eg, curriculum vitae)

• Name and address of any local laboratory conducting tests for the study, and a dated copy of current laboratory normal ranges for these tests, when applicable

• Local laboratory documentation demonstrating competence and test reliability (eg, accreditation/license), when applicable

16.2.3 Record Retention

In compliance with the ICH/GCP guidelines, the investigator/institution will maintain all eCRFs and all source documents that support the data collected from each subject, as well as all study documents, including imaging scans as specified in ICH/GCP Section 8, Essential Documents for the Conduct of a Clinical Trial, and all study documents as specified by the applicable regulatory requirement(s). The investigator/institution will take measures to prevent accidental or premature destruction of these documents.

Essential documents must be retained until at least 2 years after the last approval of a marketing application in an ICH region and until there are no pending or contemplated marketing applications in an ICH region or until at least 2 years have elapsed since the formal discontinuation of clinical development of the investigational product. These documents will be retained for a longer period when required by the applicable regulatory requirements or by an agreement with the sponsor. It is the responsibility of the sponsor to inform the investigator/institution as to when these documents no longer need to be retained. If the responsible investigator retires, relocates, or for other reasons withdraws from the responsibility of keeping the study records, then custody must be transferred to a person who will accept the responsibility. The sponsor must be notified in writing of the name and address of the new custodian. Under no circumstance shall the investigator relocate or dispose of any study documents before having obtained written approval from the sponsor.

If it becomes necessary for the sponsor or the appropriate regulatory authority to review any documentation relating to this study, then the investigator must permit access to such reports.

16.3 Study Completion/Termination

16.3.1 Study Completion

The study is considered completed with the last study assessment for the last subject participating in the study. The final data from the investigational site will be sent to the sponsor (or designee) after completion of the final subject assessment at that site, in the time frame specified in the Clinical Trial Agreement. The subjects who are benefiting from the study treatment, regardless of the treatment arm they have been assigned to, at week 25 are allowed to continue receiving this medication in an extension phase.

16.3.2 Study Termination

The sponsor reserves the right to close the investigational site or terminate the study at any time for any reason at the sole discretion of the sponsor. Investigational sites will be closed upon study completion. An investigational site is considered closed when all required documents and study supplies have been collected and a site closure visit has been performed.

The investigator may initiate site closure at any time, provided there is reasonable cause and sufficient notice is given in advance of the intended termination.

Reasons for the early closure of an investigational site by the sponsor or investigator may include but are not limited to:

• Failure of the investigator to comply with the protocol, the requirements of the IEC/IRB or local health authorities, the sponsor's procedures, or GCP guidelines

• Inadequate recruitment of subjects by the investigator

• Discontinuation of further drug development

16.3.3 On-Site Audits

Representatives of the sponsor's clinical quality assurance department may visit the site at any time during or after completion of the study to conduct an audit of the study in compliance with regulatory guidelines and company policy. These audits will require access to all study records, including source documents, for inspection and comparison with the eCRFs. Subject privacy must, however, be respected. The investigator and staff are responsible for being present and available for consultation during routinely scheduled site audit visits conducted by the sponsor or its designees.

Similar auditing procedures may also be conducted by agents of any regulatory body, either as part of a national GCP compliance program or to review the results of this study in support of a regulatory submission. The investigator should immediately notify the sponsor when they have been contacted by a regulatory agency concerning an upcoming inspection.

16.4 Use of Information and Publication

All information, including but not limited to information regarding abiraterone acetate and apalutamide or the Marketing Authorization Holder (MAH)'s operations (eg, patent application, formulas, manufacturing processes, basic scientific data, prior clinical data, formulation information) supplied by the sponsor to the investigator and not previously published, and any data, including exploratory biomarker data, generated as a result of this study, are considered confidential and remain the sole property of the sponsor. The investigator agrees to maintain this information in confidence and use this information only to accomplish this study, and will not use it for other purposes without the sponsor's prior written consent.

The investigator understands that the information developed in the clinical study will be used by the MAH in connection with the continued development of abiraterone acetate and apalutamide, and thus may be disclosed as required to other clinical investigators or regulatory agencies. To permit the information derived from the clinical studies to be used, the investigator is obligated to provide the sponsor with all data obtained in the study.

17 Publication policy

The results of the study will be reported in a Clinical Study Report generated by the LACOG and investigators and will contain eCRF data from all study sites that participated in the study, and direct transmission of clinical laboratory data. Recruitment performance or specific expertise related to the nature and the key assessment parameters of the study will be used to determine a coordinating investigator. Study subject identifiers will not be used in publication of results. Any work created in connection with performance of the study and contained in the data that can benefit from copyright protection (except any publication by

the investigator as provided for below) shall be the property of the sponsor as author and owner of copyright in such work. Consistent with Good Publication Practices and International Committee of Medical Journal Editors guidelines, the sponsor shall have the right to publish such primary (multicenter) data and information without approval from the investigator. The investigator has the right to publish study site-specific data after the primary data are published. If an investigator wishes to publish information from the study, a copy of the manuscript must be provided to the sponsor for review at least 60 days before submission for publication or presentation. Expedited reviews will be arranged for abstracts, poster presentations, or other materials. In the event that issues arise regarding scientific integrity or regulatory compliance, the sponsor will review these issues with the investigator. The sponsor will not mandate modifications to scientific content and does not have the right to suppress information. For multicenter study designs and substudy approaches, secondary results generally should not be published before the primary endpoints of a study have been published. Similarly, investigators will recognize the integrity of a multicenter study by not submitting for publication data derived from the individual study site until the combined results from the completed study have been submitted for publication, within 12 months of the availability of the final data (tables, listings, graphs), or the sponsor confirms there will be no multicenter study publication. Authorship of publications resulting from this study will be based on the guidelines on authorship, such as those described in the Uniform Requirements for Manuscripts Submitted to Biomedical Journals, which state that the named authors must have made a significant contribution to the design of the study or analysis and interpretation of the data, provided critical review of the paper, and given final approval of the final version.

Registration of Clinical Studies and Disclosure of Results
The sponsor will register or disclose the existence of and the results of clinical studies as required by law.

18 References

1. Hershman DL, Unger JM, Wright JD, et al: Adverse Health Events Following Intermittent and Continuous Androgen Deprivation in Patients With Metastatic Prostate Cancer. JAMA Oncol:1-9, 2015
2. Rozet F, Roumeguere T, Spahn M, et al: Non-metastatic castrate-resistant prostate cancer: a call for improved guidance on clinical management. World J Urol, 2016
3. Crawford ED, Higano CS, Shore ND, et al: Treating Patients with Metastatic Castration Resistant Prostate Cancer: A Comprehensive Review of Available Therapies. J Urol 194:1537-47, 2015
4. Gartrell BA, Coleman R, Efstathiou E, et al: Metastatic Prostate Cancer and the Bone: Significance and Therapeutic Options. Eur Urol 68:850-8, 2015
5. Barrie SE, Potter GA, Goddard PM, et al: Pharmacology of novel steroidal inhibitors of cytochrome P450(17) alpha (17 alpha-hydroxylase/C17-20 lyase). J Steroid Biochem Mol Biol 50:267-73, 1994

6. Attard G, Reid AH, A'Hern R, et al: Selective inhibition of CYP17 with abiraterone acetate is highly active in the treatment of castration-resistant prostate cancer. J Clin Oncol 27:3742-8, 2009

7. Attard G, Reid AH, Yap TA, et al: Phase I clinical trial of a selective inhibitor of CYP17, abiraterone acetate, confirms that castration-resistant prostate cancer commonly remains hormone driven. J Clin Oncol 26:4563-71, 2008

8. Danila DC, Morris MJ, de Bono JS, et al: Phase II multicenter study of abiraterone acetate plus prednisone therapy in patients with docetaxel-treated castration-resistant prostate cancer. J Clin Oncol 28:1496-501, 2010

9. O'Donnell A, Judson I, Dowsett M, et al: Hormonal impact of the 17alpha-hydroxylase/C(17,20)-lyase inhibitor abiraterone acetate (CB7630) in patients with prostate cancer. Br J Cancer 90:2317-25, 2004

10. Reid AH, Attard G, Danila DC, et al: Significant and sustained antitumor activity in post-docetaxel, castration-resistant prostate cancer with the CYP17 inhibitor abiraterone acetate. J Clin Oncol 28:1489-95, 2010

11. Ryan CJ, Shah S, Efstathiou E, et al: Phase II study of abiraterone acetate in chemotherapy-naive metastatic castration-resistant prostate cancer displaying bone flare discordant with serologic response. Clin Cancer Res 17:4854-61, 2011

12. Ryan CJ, Smith MR, Fong L, et al: Phase I clinical trial of the CYP17 inhibitor abiraterone acetate demonstrating clinical activity in patients with castration-resistant prostate cancer who received prior ketoconazole therapy. J Clin Oncol 28:1481-8, 2010

13. de Bono JS, Logothetis CJ, Molina A, et al: Abiraterone and increased survival in metastatic prostate cancer. N Engl J Med 364:1995-2005, 2011

14. Fizazi K, Scher HI, Molina A, et al: Abiraterone acetate for treatment of metastatic castration-resistant prostate cancer: final overall survival analysis of the COU-AA-301 randomised, double-blind, placebo-controlled phase 3 study. Lancet Oncol 13:983-92, 2012

15. Ryan CJ, Smith MR, de Bono JS, et al: Abiraterone in metastatic prostate cancer without previous chemotherapy. N Engl J Med 368:138-48, 2013

16. Rathkopf DE, Morris MJ, Fox JJ, et al: Phase I study of ARN-509, a novel antiandrogen, in the treatment of castration-resistant prostate cancer. J Clin Oncol 31:3525-30, 2013

17. Clegg NJ, Wongvipat J, Joseph JD, et al: ARN-509: a novel antiandrogen for prostate cancer treatment. Cancer Res 72:1494-503, 2012

18. Rathkopf DE, Antonarakis ES, Shore ND, et al: ARN-509 in men with metastatic castration-resistant prostate cancer (mCRPC). J Clin Oncol 31:abstr 48, 2013

19. Efstathiou E, Titus MA, Wen S, et al: Enzalutamide (ENZA) in combination with abiraterone acetate (AA) in bone metastatic castration resistant prostate cancer (mCRPC). J Clin Oncol 32:abstr 5000, 2014

20. Tombal B, Borre M, Rathenborg P, et al: Enzalutamide monotherapy in hormone-naive prostate cancer: primary analysis of an open-label, single-arm, phase 2 study. Lancet Oncol 15:592-600, 2014

21. Posadas EM, Chi KN, De Wit R, et al: Phase 1b study of ARN-509 with abiraterone acetate (AA) and prednisone (P) in patients (pts) with metastatic castration-resistant prostate cancer (mCRPC). J Clin Oncol 33:abstr 5028, 2015

22. Sweeney CJ, Chen YH, Carducci M, et al: Chemohormonal Therapy in Metastatic Hormone-Sensitive Prostate Cancer. N Engl J Med 373:737-46, 2015
23. James ND, Sydes MR, Clarke NW, et al: Addition of docetaxel, zoledronic acid, or both to first-line long-term hormone therapy in prostate cancer (STAMPEDE): survival results from an adaptive, multiarm, multistage, platform randomised controlled trial. Lancet, 2015
24. Zhang LM, Jiang HW, Tong SJ, et al: Prostate-specific antigen kinetics under androgen deprivation therapy and prostate cancer prognosis. Urol Int 91:38-48, 2013
25. Hussain M, Tangen CM, Higano C, et al: Absolute prostate-specific antigen value after androgen deprivation is a strong independent predictor of survival in new metastatic prostate cancer: data from Southwest Oncology Group Trial 9346 (INT-0162). J Clin Oncol 24:3984-90, 2006
26. Eisenhauer EA, Therasse P, Bogaerts J, et al: New response evaluation criteria in solid tumours: revised RECIST guideline (version 1.1). Eur J Cancer 45:228-47, 2009
27. Chang D, Joseph DJ, Ebert MA, et al: Effect of androgen deprivation therapy on muscle attenuation in men with prostate cancer. J Med Imaging Radiat Oncol 58:223-8, 2014
28. Scher HI, Morris MJ, Stadler WM, et al: Trial design and objectives for castration-resistant prostate cancer: update recommendations from the prostate cancer clinical trials working group 3. J Clin Oncol 34: 1402-18, 2016
29. Lim L, Lau N, Garret-Engele P et al. Microarray analysis shows that some microRNAs downregulate large numbers of target mRNAs. Nature 2005;433:769-73.
30. Porkka K, Pfeiffer M, Waltering K, Vessella R, Tammela T, Visakorpi T. MicroRNA expression profiling in Prostate cancer. Cancer Res 2007;67:6130-6135.
31. Karantanos T, Corn PG, Thompson TC. Prostate cancer progression after androgen deprivation therapy: mechanisms of castrate resistance and novel therapeutic approaches. Oncogene. 2013 Dec 5;32(49):5501-11
