# Supplementary material for: Androgen Receptor Pathway Inhibitor Therapy for Advanced Prostate Cancer: Secondary Analysis of a Randomized Clinical Trial
Source: JAMA Netw Open. 2025 Jan 13;8(1):e2454253. doi: 10.1001/jamanetworkopen.2024.54253 (PMC11731179; doi:10.1001/jamanetworkopen.2024.54253)
Supplement: Supplement 2. — eFigure 1. Mean FACT-P (A) and FACT-G (B) Total Scores at Baseline and During Treatment in the 3 Arms eFigure 2. Mean FACT-P Subscale Scores at Baseline and During Treatment in the 3 Arms eTable 1. Baseline Characteristics eTable 2. Health-Related Quality of Life (HRQoL) Scores eTable 3. Treatment-Related Adverse Events eTable 4. Adjusted Mean Changes in HRQoL Scores From Baseline to Week 25 (MMRM and Sensitivity Analysis) eAppendix 1. Eligibility Criteria eFigure 3. Trial Flow Diagram eAppendix 2. Randomization Procedures [file jamanetwopen-e2454253-s002.pdf]

Supplementary Online Content

Bastos DA, Soares A, Schutz FAB, et al. Androgen receptor pathway inhibitor therapy for advanced prostate cancer: secondary analysis of a randomized clinical trial. *JAMA Netw Open.* 2025;8(1):e2454253. doi:10.1001/jamanetworkopen.2024.54253

eFigure 1. Mean FACT-P (A) and FACT-G (B) Total Scores at Baseline and During Treatment in the 3 Arms
eFigure 2. Mean FACT-P Subscale Scores at Baseline and During Treatment in the 3 Arms
eTable 1. Baseline Characteristics
eTable 2. Health-Related Quality of Life (HRQoL) Scores
eTable 3. Treatment-Related Adverse Events
eTable 4. Adjusted Mean Changes in HRQoL Scores From Baseline to Week 25 (MMRM and Sensitivity Analysis)
eAppendix 1. Eligibility Criteria
eFigure 3. Trial Flow Diagram
eAppendix 2. Randomization Procedures

This supplementary material has been provided by the authors to give readers additional information about their work.

A

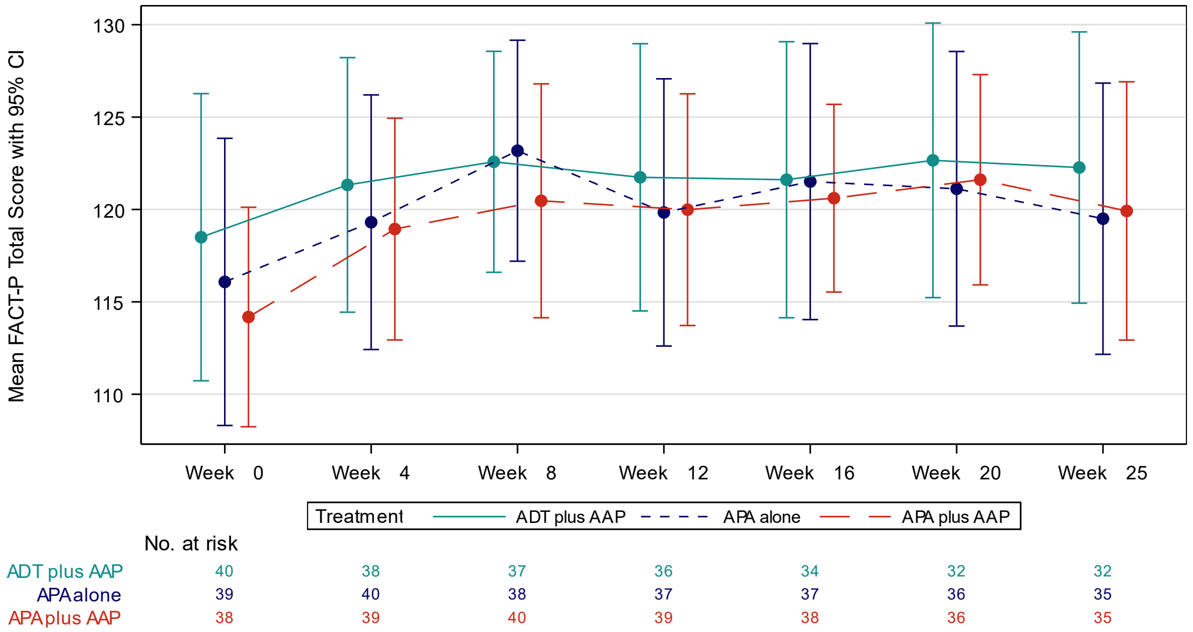

B

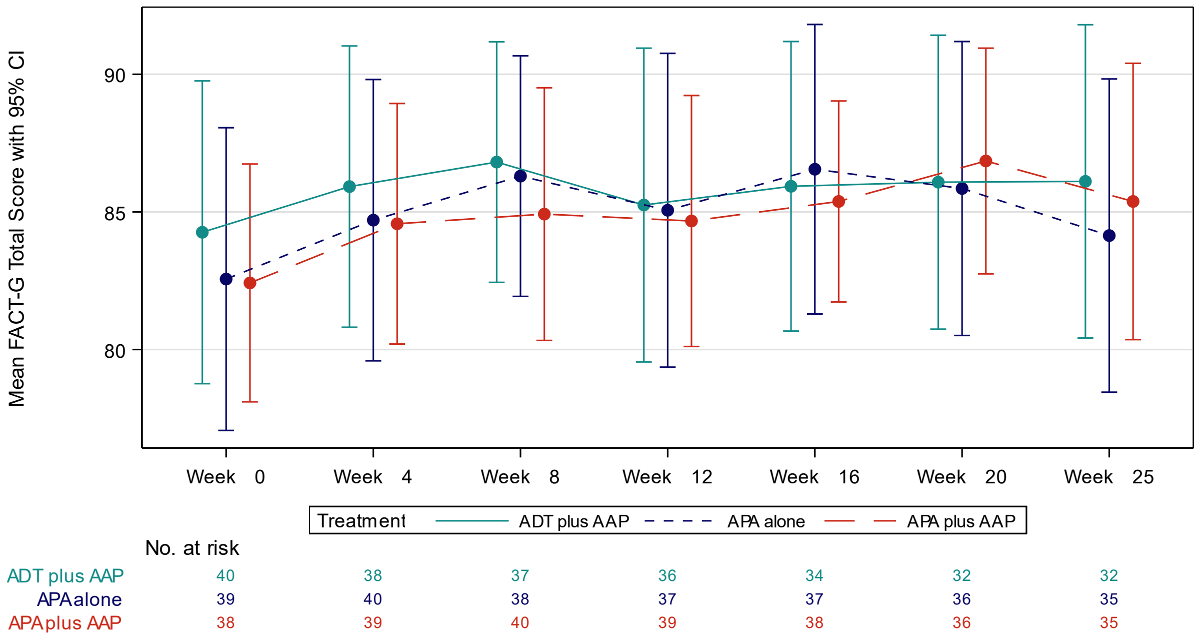

eFigure 1. Mean FACT-P (A) and FACT-G (B) Total Scores at Baseline and During Treatment in the 3 Arms

© 2025 Bastos DA et al. *JAMA Network Open.*

A

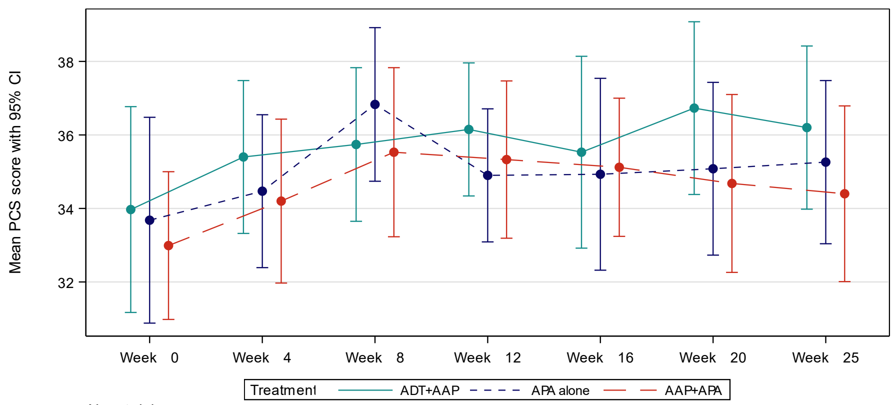

No. at risk							
ADT+AAP	40	38	37	36	34	32	32
APA alone	39	40	38	37	37	36	35
AAP+APA	38	39	40	39	38	36	35

B

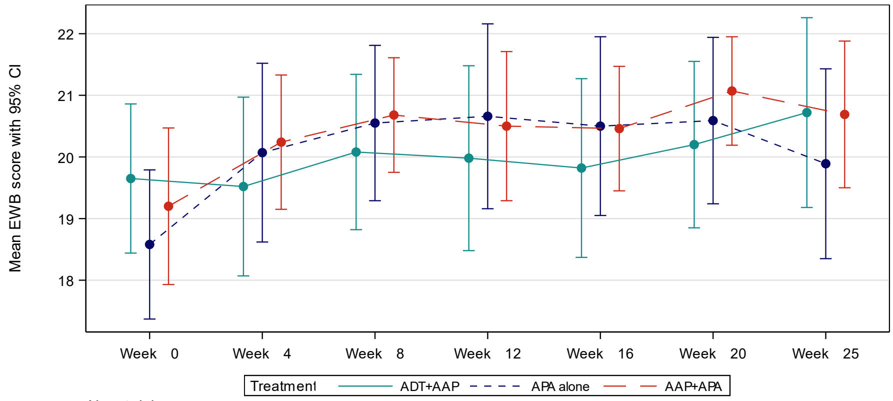

No. at risk							
ADT+AAP	40	38	37	36	34	32	32
APA alone	39	40	38	37	37	36	35
AAP+APA	38	39	40	39	38	36	35

C

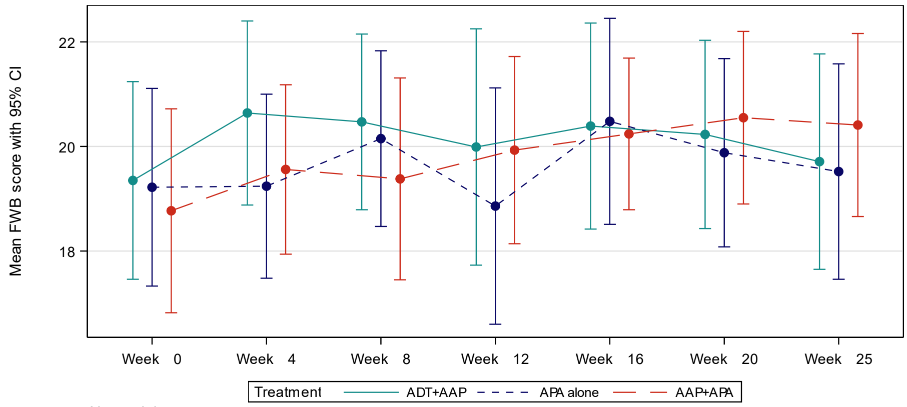

No. at risk							
ADT+AAP	40	38	37	36	34	32	32
APA alone	39	40	38	37	37	36	35
AAP+APA	38	39	40	39	38	36	35

© 2025 Bastos DA et al. *JAMA Network Open.*

D

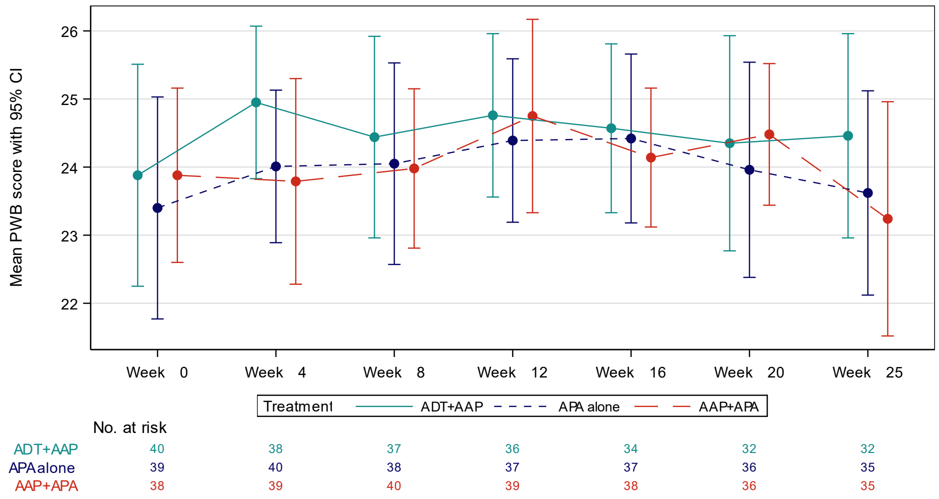

E

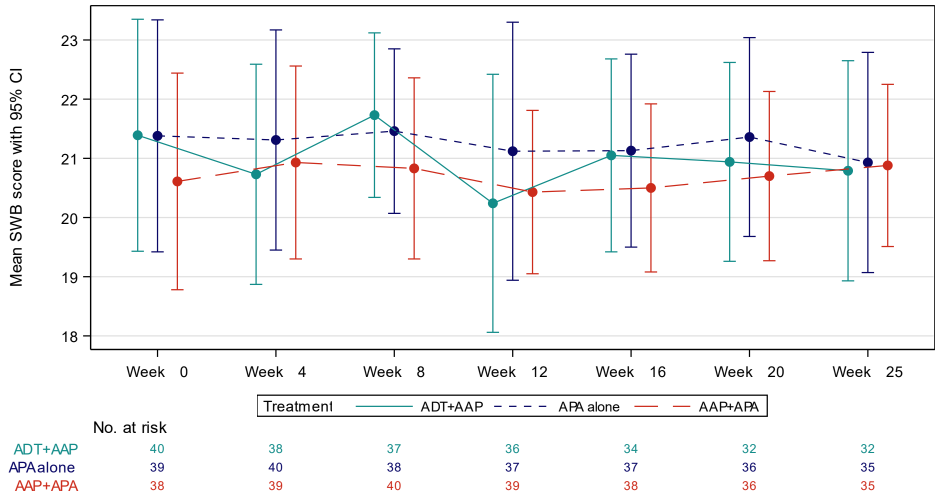

eFigure 2. Mean FACT-P Subscale Scores at Baseline and During Treatment in the 3 Arms. **A:** Prostate Cancer Subscale (PCS) score. **B:** Emotional well-being (EWB) subscale scores. **C:** Functional well-being (FWB) subscale scores. **D:** Physical well-being (PWB) subscale scores. **E:** Social well-being (SWB) subscale scores.

© 2025 Bastos DA et al. *JAMA Network Open.*

eTable 1. Baseline Characteristics

Characteristic	ADT plus AAP (N = 42), No. (%)	APA (N = 42), No. (%)	APA plus AAP (N = 44), No. (%)
**Median age, yr (IQR)**	69.8 (58.9-71.6)	69.5 (59.8-72.6)	71.0 (63.0-72.3)
**Median PSA, ng/mL((IQR)**	16.7 (6.4-50.0)	19.9 (7.2-68.5)	32.4 (7.1-141.5)
**Median testosterone, ng/dL (IQR)**	424.7 (331.0-469.1)	434.5 (360.0-532.9)	413 (312.4-518.0)
**ECOG 0-1**†	41 (97.6)	42 (100)	44 (100)
**Gleason score**			
≤6	3 (7.1)	2 (4.8)	5 (11.4)
7	18 (42.9)	17 (40.5)	10 (22.7)
≥8	21 (50.0)	23 (54.7)	29 (65.9)
**Disease status at study entry**			
Biochemical recurrence	7 (16.7)	8 (19.0)	7 (15.9)
Locally advanced disease	6 (14.3)	2 (4.8)	3 (6.8)
Metastatic disease	29 (69.0)	32 (76.2)	34 (77.3)

PSA= prostate-specific antigen.
HRQoL= health-related quality of life.
IQR= interquartile range.
†Scores for the Eastern Cooperative Oncology Group (ECOG) performance status are assessed on a 5-point scale.

© 2025 Bastos DA et al. *JAMA Network Open.*

eTable 2. Health-Related Quality of Life (HRQoL) Scores

HRQoL Scores	ADT + AAP	APA alone	AAP + APA	Δ mean change from baseline among treatment arms	*P*-value
**Adjusted mean changes from baseline at week 25 - MMRM analysis, adjusted least square means (95% CI)**					
Physical well-being	0.6 (1.0)	0.2 (0.9)		-0.4 (-2.7 to 1.9)	0.46
	0.6 (1.0)		-0.6 (0.9)	-1.2 (-3.3 to 0.9)	0.29
		0.2 (0.9)	-0.6 (0.9)	-0.8 (-2.9 to 1.4)	0.74
Functional well-being	0.4 (1.0)	0.3 (1.0)		-0.1 (-3.5 to 1.2)	0.88
	0.4 (1.0)		1.6 (1.0)	1.2 (1.1 to 3.6)	0.56
		0.3 (1.0)	1.6 (1.0)	1.3 (-1.0 to 3.6)	0.46
Social well-being	-0.1 (0.6)	-0.4 (0.7)		-0.3 (-2.4 to 1.8)	0.90
	-0.1 (0.6)		-0.1 (0.7)	0.0 (-2.1 to 2.1)	0.93
		-0.4 (0.7)	-0.1 (0.7)	0.3 (-1.8 to 2.5)	0.96
Emotional well-being	1.1 (0.6)	1.3 (0.6)		0.2 (-1.5 to 2.0)	0.35
	1.1 (0.6)		1.5 (0.5)	0.4 (-1.3 to 2.2)	0.98
		1.3 (0.6)	1.5 (0.5)	0.2 (-1.5 to 2.0)	0.36
Prostate cancer subscale	2.1 (1.2)	1.6 (1.2)		-0.5 (-3.4 to 2.4)	0.52
	2.1 (1.2)		1.4 (1.2)	-0.7 (-3.6 to 2.2)	0.22
		1.6 (1.2)	1.4 (1.2)	-0.2 (-2.9 to 2.5)	0.55
FACT-G total score	2.2 (1.8)	2.7 (1.7)		0.5 (-6.4 to 7.4)	0.57
	2.2 (1.8)		2.7 (2.0)	0.5 (-6.2 to 7.2)	0.83
		2.7 (1.7)	2.7 (2.0)	0.0 (-6.6 to 6.6)	0.72
FACT-P total score	4.8 (2.4)	5.0 (2.4)		0.2 (-8.8 to 9.2)	0.54
	4.8 (2.4)		4.2 (2.8)	-0.6 (-9.4 to 8.2)	0.61
		5.0 (2.4)	4.2 (2.8)	-0.8 (-9.6 to 8.0)	0.93

FACT-G= Functional Assessment of Cancer Therapy–General.
FACT-P= Functional Assessment of Cancer Therapy–Prostate.
HRQoL= health-related quality of life.
MMRM= mixed model for repeated measures.

© 2025 Bastos DA et al. *JAMA Network Open.*

eTable 3. Treatment-Related Adverse Events

Adverse Event	ADT plus AAP (N=42)		APA alone (N=42)		APA plus AAP (N=44)	
	**All grade**	**Grade 3-4**	**All grade**	**Grade 3-4**	**All grade**	**Grade 3-4**
	number of patients with event (percent)					
Breast pain	0 (0)	0	6 (14)	0	2 (5)	0
Erectile dysfunction	6 (14)	0	4 (10)	0	1 (2)	0
Fall	1 (2)	0	4 (10)	1 (2)	1 (2)	0
Fatigue	6 (14)	0	9 (21)	1 (2)	10 (23)	0
Gynecomastia	3 (7)	0	23 (55)	1 (2)	10 (23)	0
Hot flashes	15 (36)	0	2 (5)	0	12 (27)	0
Hyperglycemia	2 (5)	1 (2)	1 (2)	1 (2)	3 (7)	2 (5)
Hypertension arterial	8 (19)	3 (7)	1 (2)	0	8 (18)	4 (9)
Loss of libido	4 (10)	0	3 (7)	0	2 (5)	0
Nausea	2 (5)	0	2 (5)	0	5 (11)	0
Pruritus	1 (2)	0	6 (14)	1 (2)	3 (7)	2 (5)
Rash	0 (0)	0	8 (19)	5 (12)	9 (20)	3 (7)

All events occurring in ≥10% of patients in any arm.

© 2025 Bastos DA et al. *JAMA Network Open.*

eTable 4. Adjusted Mean Changes in HRQoL Scores From Baseline to Week 25 (MMRM and Sensitivity Analysis)

		Mixed model with repeated measures			PMM with control-based pattern imputation		
**HRQoL score**	**Arm**	**Adjusted Mean Difference**	**Standard Error**	**P-value**	**Adjusted Mean Difference**	**Standard Error**	**P-value**
Emotional well-being (EWB)	APA alone - ADT+AAP	0.24	0.9	0.3	0.3	0.7	0.8
	AAP+APA - ADT+AAP	0.4	0.9	1	0.4	0.7	0.5
	AAP+APA - APA alone	0.2	0.9	0.4	0.1	0.7	0.6
Functional well-being (FWB)	APA alone - ADT+AAP	-0.1	1.2	0.9	0.1	1.1	0.9
	AAP+APA - ADT+AAP	1.3	1.2	0.6	1.2	1.1	0.4
	AAP+APA - APA alone	1.3	1.2	0.5	1.2	1.1	0.5
Prostate cancer subscale (PCS)	APA alone - ADT+AAP	-0.5	1.5	0.5	-0.4	1.3	0.9
	AAP+APA - ADT+AAP	-0.7	1.5	0.2	-1.1	1.3	0.4
	AAP+APA - APA alone	-0.2	1.4	0.6	-0.7	1.3	0.7
Physical well-being (PWB)	APA alone - ADT+AAP	-0.4	1.1	0.5	-0.7	1.1	0.7
	AAP+APA - ADT+AAP	-1.2	1.1	0.3	-1.3	1.1	0.6
	AAP+APA - APA alone	-0.9	1.1	0.7	-0.6	1.1	0.9
Social well-being (SWB)	APA alone - ADT+AAP	-0.3	1.1	0.9	0.1	1.0	0.7
	AAP+APA - ADT+AAP	0.1	1.1	0.9	0.8	1.0	0.5
	AAP+APA - APA alone	0.4	1.1	1	0.8	1.0	0.7
FACT-P	APA alone - ADT+AAP	0.2	4.5	0.5	-0.6	3.5	0.7
	AAP+APA - ADT+AAP	-0.6	4.5	0.6	1.2	3.5	0.9
	AAP+APA - APA alone	-0. 8	4.5	0.9	1.7	3.5	0.8
FACT-G	APA alone - ADT+AAP	0.5	3.4	0.6	-0.7	2.7	0.9
	AAP+APA - ADT+AAP	0.4	3.4	0.8	0.8	2.7	0.8
	AAP+APA - APA alone	-0.1	3.4	0.7	1.5	2.6	0.7

© 2025 Bastos DA et al. *JAMA Network Open.*

eAppendix 1. Eligibility Criteria

Inclusion criteria:
Each potential subject must fulfill all of the following criteria to be enrolled in the study.

1. Histologically confirmed prostate adenocarcinoma;
2. Patients with indication to start treatment with ADT in one of the following settings:
   a. Biochemical relapse after definitive treatment (surgery and/or radiotherapy): PSA ≥ 4 ng/ml and doubling time less than 10 months, or PSA ≥ 20 ng/ml;
   b. Newly diagnosed Prostate Cancer: locally advanced – T_{any} N+ M0 (not candidate to definitive treatment with surgery or radiotherapy) or metastatic – T_{any} N_{any} M+ and PSA ≥ 2 ng/mL;
3. Patient is asymptomatic to moderately symptomatic regarding bone symptoms, i.e., no need for palliative radiation or radionuclide therapy;
4. Complete staging process (performed as per routine), meaning, thorax, abdomen and pelvis TC and bone scan, performed before consent and that do not exceed 8 weeks from the date of randomization;
5. Non-castration level of testosterone □ 230ng/dL (> 8 nmol/L);
6. ECOG performance status of 0 to 2;
7. Adequate hematologic, hepatic and renal function:

a. hemoglobin > 10 g/dL, neutrophils > 1.5×10^9 / L, platelets> 100×10^9 / L;
b. total bilirubin < 1.5x upper limit of normal (ULN); alanine (ALT) and aspartate (AST) aminotransferase < 2.5 x ULN;
c. serum creatitine < 1.5x ULN; potassium > 3.5 mM;

8. Written informed consent obtained prior to any study procedure;
9. Men age 18 years and older;
10. Agrees to use a condom and another effective method of birth control if he is having sex with a woman of childbearing potential or agrees to use a condom if he is having sex with a woman who is pregnant.

Exclusion criteria:
Any potential subject who meets any of the following criteria will be excluded from participating in the study.

1. Prostate adenocarcinoma with neuroendocrine differentiation or small cell histology;
2. Use of hormonal therapy or chemotherapy prior to randomization. Exception is courses of hormone therapy for localised disease must have been completed at least 12 months previously. It can have been given as adjuvant or neoadjuvant therapy.
3. Prior radiation therapy for a primary tumour within the 3 months before enrollment or for the treatment of metastases;
4. Known or suspected brain or skull metastases or leptomeningeal metastatic disease;

5.Any concurrent severe and/or uncontrolled medical conditions which could compromise participation

© 2025 Bastos DA et al. *JAMA Network Open.*

in the study;

6. Administration of an investigational therapeutic or invasive surgical procedure within 28 days of Cycle 1 Day 1 or currently enrolled in an investigational study;

7. Active or symptomatic viral hepatitis or chronic liver disease; ascites or bleeding disorders secondary to hepatic dysfunction;

8. Current or prior treatment with anti-epileptic medications for the treatment of seizures;

9. Impaired cardiac function, including any of the following:
   a. Uncontrolled hypertension (systolic blood pressure ≥160 mmHg or diastolic BP ≥95 mmHg);

   b. Clinically significant heart disease as evidenced by myocardial infarction, or arterial thrombotic events or history of cardiac failure in the past 6 months, severe or unstable angina, or New York Heart Association (NYHA) Class II-IV heart disease;

   c. Existing atrial fibrillation with or without pharmacotherapy. Other cardiac arrhythmia requiring pharmacotherapy;

10. History of seizure or condition that may predispose to seizure (including, but not limited to prior stroke, transient ischemic attack or loss of consciousness ≤1 year prior to randomization; brain arteriovenous malformation; or intracranial masses such as schwannomas and meningiomas that are causing edema or mass effect);
11. Specific underlying conditions for oral agents. For example: impairment of gastrointestinal (GI) function or GI disease that may significantly alter the absorption of abiraterone or APALUTAMIDE (e.g., ulcerative diseases, uncontrolled nausea, vomiting, diarrhea, malabsorption syndrome, or small bowel resection)
12. General excluded medications (e.g., relevant to cytochrome P450 interactions)
    a. Use of prescription drugs within 14 days prior to dosing or over-the-counter (OTC) medication within 7 days prior to dosing;
    b. Consumption of grapefruit product or St John’s wort within 7 days prior to dosing;
    c. G-CSF, GM-CSF, erythropoietin, etc;
    d. Coumadin;
    e. Drugs which may cause QT prolongation;
    f. Known sensitivity to drugs or metabolites from similar classes;
    g. Known or suspected contraindications or hypersensitivity to APALUTAMIDE, bicalutamide or GnRH agonists or any of the components of the formulations;
13. Any condition or situation which, in the opinion of the investigator, would put the subject at risk, may confound study results, or interfere with the subject’s participation in this study;
14. Surgical castration prior to study entry;
15. Had a prior malignancy. Adequately treated basal cell or squamous cell carcinoma of skin or superficial bladder cancer that has not spread behind the connective tissue layer (i.e., pTis, pTa, and pT1) is allowed, as well as any other cancer for which treatment has been completed 5 years before randomization and from which the subject has been disease-free.

© 2025 Bastos DA et al. *JAMA Network Open.*

eFigure 3. Trial Flow Diagram

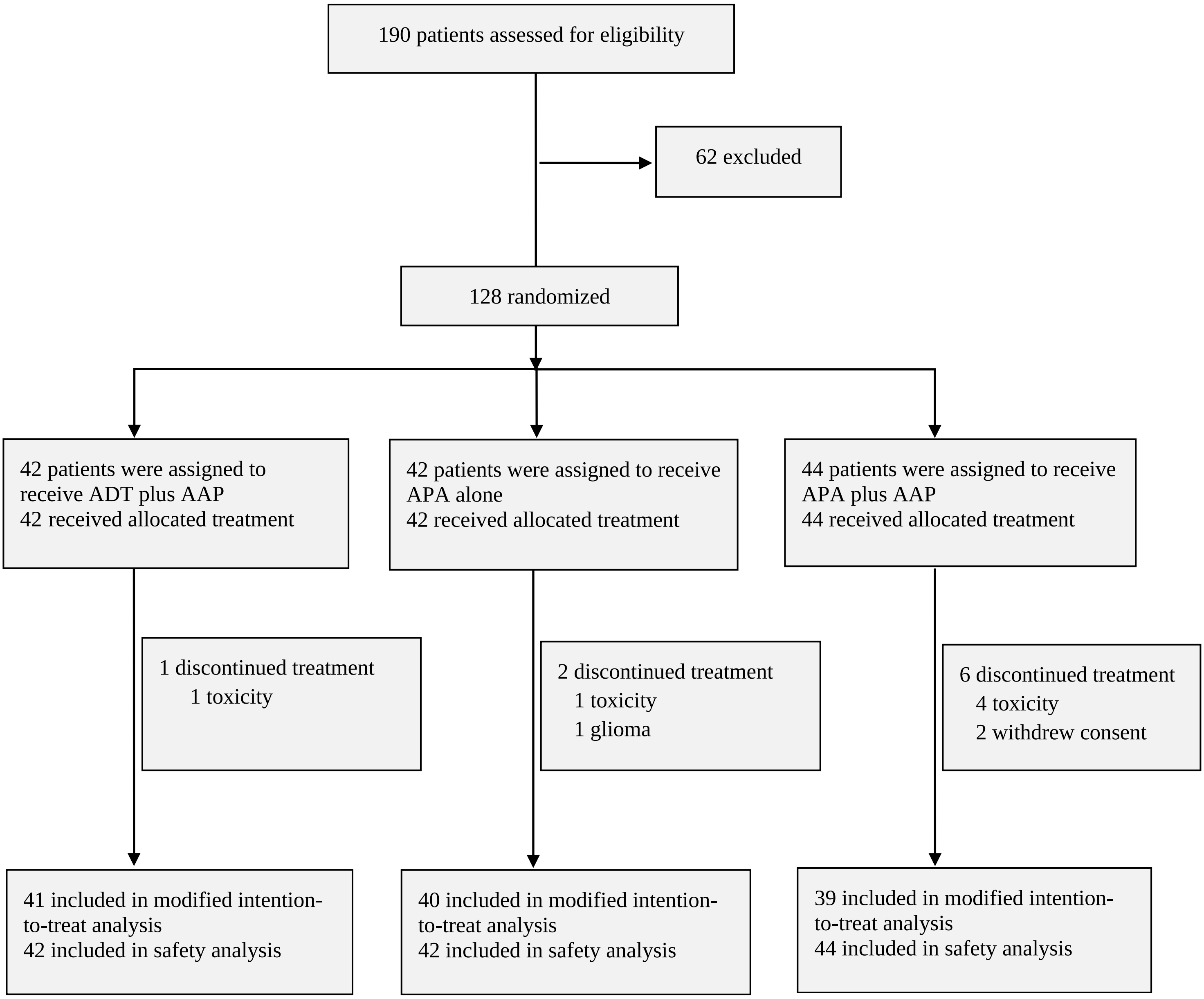

© 2025 Bastos DA et al. *JAMA Network Open.*

eAppendix 2. Randomization Procedures

Patients had all eligibility criteria reviewed, patients who fulfilled all eligibility criteria were randomized. Randomization was centrally performed by LACOG through a randomization system (http://www.randomize.net/), using a method to obtain adequate between-arm balance for performance status (ECOG 0-1 versus 2) and metastatic disease (yes vs. no). Randomization was balanced by using randomly permuted blocks. The investigators and patients were not blinded to treatment assignments. Patients are allowed to initiate the study treatment within 72 hours after randomization.

© 2025 Bastos DA et al. *JAMA Network Open.*
