# Supplementary material for: Androgen Receptor Pathway Inhibitor Therapy for Advanced Prostate Cancer: Secondary Analysis of a Randomized Clinical Trial
Source: JAMA Netw Open. 2025 Jan 13;8(1):e2454253. doi: 10.1001/jamanetworkopen.2024.54253 (PMC11731179; doi:10.1001/jamanetworkopen.2024.54253)
Supplement: Supplement 3. — Data Sharing Statement [file jamanetwopen-e2454253-s003.pdf]

Data Sharing Statement

Bastos. Androgen Receptor Pathway Inhibitor Therapy for Advanced Prostate Cancer. *JAMA Netw Open*. Published January 13, 2025. doi:10.1001/jamanetworkopen.2024.54253

Data
Additional Information: NCT02867020
Data available: Yes
Data types: Other (please specify)
Additional Information: Study protocol, data, including trial-level data (analysis datasets), as well as other information (e.g., clinical study reports, or analysis plans), are available for request.
How to access data: The study protocol was published online under the Digital Object Identifier (DOI) https://doi.org/10.1186/s12885-019-5709-y. Study protocol, data, including trial-level data (analysis datasets), as well as other information (e.g., clinical study reports, or analysis plans), are available upon request to the investigators. These clinical trial data can be requested by any qualified researchers who engage in rigorous, independent, scientific research and will be provided following review and approval of a research proposal, statistical analysis plan, and execution of a data sharing agreement. Data requests can be submitted to the corresponding author at any time after acceptance of this manuscript for publication.
When available: With publication

Supporting Documents
Document types: None

Additional Information
Who can access the data: These clinical trial data can be requested by any qualified researchers who engage in rigorous, independent, scientific research and will be provided following review and approval of a research proposal, statistical analysis plan, and execution of a data sharing agreement. Data requests can be submitted to the corresponding author at any time after acceptance of this manuscript for publication.
Types of analyses: Data will be made available for any type of analysis, contingent upon the review and approval of a research proposal, statistical analysis plan, and execution of a data sharing agreement.
Mechanisms of data availability: Data will be made available after approval of a proposal, or with a signed data access agreement.
